# Brain Volume Changes after COVID-19 Compared to Healthy Controls by Artificial Intelligence-Based MRI Volumetry

**DOI:** 10.3390/diagnostics13101716

**Published:** 2023-05-12

**Authors:** Zeynep Bendella, Catherine Nichols Widmann, Julian Philipp Layer, Yonah Lucas Layer, Robert Haase, Malte Sauer, Luzie Bieler, Nils Christian Lehnen, Daniel Paech, Michael T. Heneka, Alexander Radbruch, Frederic Carsten Schmeel

**Affiliations:** 1 Department of Neuroradiology, University Hospital Bonn, Rheinische Friedrich-Wilhelms-Universität Bonn, Venusberg-Campus 1, 53127 Bonn, Germany; zeynep.bendella@ukbonn.de (Z.B.);; 2 Department of Neurodegenerative Diseases and Geriatric Psychiatry, University Hospital Bonn, Rheinische Friedrich-Wilhelms-Universität Bonn, 53127 Bonn, Germany; 3 German Center for Neurodegenerative Diseases (DZNE), 53127 Bonn, Germany; 4 Department of Radiation Oncology, University Hospital Bonn, Rheinische Friedrich-Wilhelms-Universität Bonn, 53127 Bonn, Germany; 5 Institute of Experimental Oncology, University Hospital Bonn, Rheinische Friedrich-Wilhelms-Universität Bonn, 53127 Bonn, Germany; 6 Luxembourg Centre for Systems Biomedicine, University of Luxembourg, Esch-Belval, 4367 Luxembourg, Luxembourg

**Keywords:** SARS-CoV-2, COVID-19, magnetic resonance imaging, brain atrophy, artificial intelligence

## Abstract

Cohort studies that quantify volumetric brain data among individuals with different levels of COVID-19 severity are presently limited. It is still uncertain whether there exists a potential correlation between disease severity and the effects of COVID-19 on brain integrity. Our objective was to assess the potential impact of COVID-19 on measured brain volume in patients with asymptomatic/mild and severe disease after recovery from infection, compared with healthy controls, using artificial intelligence (AI)-based MRI volumetry. A total of 155 participants were prospectively enrolled in this IRB-approved analysis of three cohorts with a mild course of COVID-19 (n = 51, MILD), a severe hospitalised course (n = 48, SEV), and healthy controls (n = 56, CTL) all undergoing a standardised MRI protocol of the brain. Automated AI-based determination of various brain volumes in mL and calculation of normalised percentiles of brain volume was performed with mdbrain software, using a 3D T1-weighted magnetisation-prepared rapid gradient echo (MPRAGE) sequence. The automatically measured brain volumes and percentiles were analysed for differences between groups. The estimated influence of COVID-19 and demographic/clinical variables on brain volume was determined using multivariate analysis. There were statistically significant differences in measured brain volumes and percentiles of various brain regions among groups, even after the exclusion of patients undergoing intensive care, with significant volume reductions in COVID-19 patients, which increased with disease severity (SEV > MILD > CTL) and mainly affected the supratentorial grey matter, frontal and parietal lobes, and right thalamus. Severe COVID-19 infection, in addition to established demographic parameters such as age and sex, was a significant predictor of brain volume loss upon multivariate analysis. In conclusion, neocortical brain degeneration was detected in patients who had recovered from SARS-CoV-2 infection compared to healthy controls, worsening with greater initial COVID-19 severity and mainly affecting the fronto-parietal brain and right thalamus, regardless of ICU treatment. This suggests a direct link between COVID-19 infection and subsequent brain atrophy, which may have major implications for clinical management and future cognitive rehabilitation strategies.

## 1. Introduction

There is mounting evidence of brain-related pathology due to COVID-19, both during the acute phase of the disease and in the longer-term course [1]. In addition to the well-documented loss of taste and smell during the acute phase [2], individuals affected by the virus may also suffer long-term neurological, psychiatric, and neurocognitive impairments following COVID-19 infection, particularly cognitive deficits such as impaired concentration and memory, even after asymptomatic infection [3]. Persistent neurological complications have been reported in up to 25% of patients [4], although studies have demonstrated high variability in the prevalence and incidence of symptoms [5]. Given that acquired neural damage can increase the risk of initiating or exacerbating neurodegenerative processes [6], greater attention is now being paid to the long-term impact of COVID-19 on the central nervous system (CNS).

It has been postulated that patients severely ill with COVID-19 may also experience more severe CNS damage [7]. However, several case series have shown that neurological symptoms are not limited to severe cases. In fact, between 37% to 84% of patients with mild symptoms in intermediate care exhibit neurological impairment [7,8]. This implies that the severity of the initial infection may play a prominent role in long-term neurological trajectories. While most COVID-19 imaging studies to date have focused on acute and hospitalised cases with a fairly broad spectrum of gross cerebral abnormalities, such as white matter hyperintensities and cerebrovascular events, especially in the cerebrum [9], there have been fewer cohort studies that quantitatively compare volumetric brain data among subjects with different disease severity. As such, it remains unclear whether the effects of COVID-19 on the CNS can be quantitatively assessed even in milder cases, or whether they depend on the initial severity of the disease. Knowledge of such effects might reveal possible mechanisms for the spread and potential sequelae of the disease. To assess the potential impact of COVID-19 on the brain and its structural integrity, this magnetic resonance imaging (MRI) study was conducted to evaluate potential volumetric brain abnormalities among patients with asymptomatic/mild and severe cases of COVID-19 after remission of infection, in comparison to actively recruited healthy controls, using artificial intelligence (AI)-based volumetry. Utilising a volumetric approach may provide insights into possible cortical and subcortical alterations after COVID-19.

## 2. Materials and Methods

This monocentric longitudinal prospective cohort study was conducted at Bonn University Hospital and is one of three subprojects within a three-pronged research consortium known as “COVIMMUNE: Studies on immune system function and disease progression of COVID-19”, funded by the German Ministry of Health. In addition to clinical and neuropsychological examinations conducted by trained and qualified medical investigators at three timepoints (baseline, 6 and 12 months), standardised brain MR imaging was scheduled at two time points (baseline and after 12 months) for all participant groups. In this paper, we present the baseline MR imaging findings (i.e., after study enrolment) from the radiological project arm. MRI follow-up at 12 months is planned and currently pending, and therefore not yet part of the analysis below. The local Internal Review Board (the Medical Ethics Review Board of the University Hospital Bonn, ID 511/20) reviewed the study protocol, and final approval was obtained on 10 March 2021. All participants provided written informed consent before taking part in any study-specific procedures.

This study has been preregistered at the German Clinical Trials Registry (primary registry trial identifier: DRKS00023806; registration date: 16 March 2021, and cross-referenced with the World Health Organization’s International Clinical Trials Registry Platform [ICTRP]).

### 2.1. Study Population

A total of 172 participants with similar age and sex distribution were prospectively enrolled using frequency matching (as of 10 March 2022), of whom 10 failed screening and 7 were lost during the scheduled MRI examination stage due to claustrophobia (n = 4) or metallic implants (n = 3). The remaining 155 participants were categorized into three groups based on their health status: healthy control subjects (n = 56), patients with an asymptomatic/mild course (n = 51), and patients with a severe course of COVID-19 (n = 48), as explained below.

The entire study protocol was recently described elsewhere [10] and can be summarised as follows regarding the radiological project arm:

General inclusion criteria:

- aged 25 to 75 years

Cohort-specific inclusion criteria:

- Cohort I: asymptomatic course (MILD) of COVID-19 (SARS-CoV-2–positive) or mild course (i.e., declaration of no symptoms other than anosmia or ageusia)
- Cohort II: severely affected course (SEV) of COVID-19 (SARS-CoV-2–positive) according to simplified WHO classification, defined as having been admitted to hospital (any ward type) for at least 24 h due to SARS-CoV-2 infection at any timepoint during the course of the disease
- Cohort III: healthy controls (CTL) will only be included in the study if they also meet all of the following criteria:
  - must perform > –1.0 SD on the Hopkins Verbal Learning Test
  - no substance abuse
  - no known history of or current diagnosed psychiatric illness
  - negative nCoV IgG/IgM Rapid Test before inclusion, indicative of no recent COVID-19 infection

General exclusion criteria:

- general contraindication for MRI
- severe or unstable medical condition
- current major depressive episode
- psychotic disorder, bipolar disorder, substance abuse at present or in the past
- known neurodegenerative disorder (Alzheimer’s disease, Parkinson’s disease, frontotemporal dementia, Huntington’s disease, amyotrophic lateral sclerosis)
- vascular dementia or history of stroke
- history of malignant disease

### 2.2. Magnetic Resonance Imaging

All participants underwent standardised brain MRI at baseline following enrolment in the study. MR imaging was performed on a clinical whole-body 3 T MRI system (Achieva TX, Philips Healthcare, Best, The Netherlands) equipped with an 8-channel head coil with identical scanning protocols. Morphological brain imaging included three-dimensional (3D) T1-weighted magnetisation-prepared rapid acquisition with gradient echo (3D MPRAGE), 3D fluid-attenuated inversion recovery (3D-FLAIR), diffusion-weighted imaging (DWI), susceptibility-weighted imaging (SWI) and T2-weighted imaging (T2W). Details of the MRI scanning parameters are summarised in Table 1.

### 2.3. Image Analysis

Board-certified radiologists with several years of neuroradiological experience visually examined the MRI datasets of all subjects for acute cerebral pathology and possible exclusion criteria.

### 2.4. Post-Processing and Artificial Intelligence (AI)-Based Volumetry

Automated AI-based software was used to determine quantitative analyses of the volume of different brain areas in mL and age- and sex-adjusted percentiles (based on an internal reference collective of age- and sex-adjusted healthy controls from the general population embedded in the software). This commercially licensed MRI post-processing software, named “mdbrain”, is provided by mediaire GmbH, Berlin, Germany, an approved medical device manufacturer according to the European Medical Device Directive 93/42/EEC, and is certified according to DIN EN ISO 13485:2016. The “mdbrain” software is approved as a CE-marked medical device. It performs automatic brain volumetry of different brain regions using native 3D T1-weighted sequences to allow quantitative statements based on an extensive population-based normative database. The algorithm and embedded normative database are trained nationwide, not limited to the experience of a single centre, and have been validated for accuracy [11].

The 3D T1w MPRAGE sequence was manually transferred from the clinical PACS to the mdbrain software, v4.4.1 or higher, for automatic volumetrization and percentile calculation. The volumes and percentiles of all evaluated structures were automatically computed, saved and checked for plausibility. The volumetrized structures were the whole brain, whole white matter, whole grey matter, cerebral cortex, cerebellar cortex, frontal lobe, parietal lobe, precuneus, occipital lobe, temporal lobe, hippocampus, parahippocampal gyrus, entorhinal cortex, caudate nucleus, putamen, globus pallidum, thalamus, brainstem, mesencephalon, pons, lateral ventricle, third ventricle, and fourth ventricle. Volumes were determined separately for each of the paired structures.

### 2.5. Statistical Analysis

Statistical analyses were conducted using SPSS statistical software (v27 and above, IBM Corp., Armonk, NY, USA) and R (v4.2.2, R core Team, R Foundation for Statistical Computing, Vienna, Austria, URL https://www.R-project.org/ accessed on 15 March 2023) with the jtools package (v2.2.0, URL https://cran.r-project.org/package=jtools accessed on 15 March 2023). All applicable demographic and imaging data are given as mean ± standard deviation, unless otherwise specified. The statistical significance level was set at *p* < 0.05. A priori statistical power analyses were performed and recently described elsewhere [10], yielding a required total sample size of 126 at an estimated actual power of 80%. Mann–Whitney U and Kruskal–Wallis tests were used for pairwise and multiple group comparisons of independent clinical and imaging data, and a one-way ANOVA of variance with post hoc testing after Bonferroni correction was used for pairwise inter-class comparisons. A multivariate regression model was then used to analyse the independent variables further and estimate the relative contribution of demographic and clinical parameters to the observed differences between measured brain volumes. The predictors included in the model were age, sex, height, body mass index (BMI), asymptomatic/mild course (MILD) and severe course (SEV) of COVID-19. The obtained model provided a coefficient (estimate) for each predictor, allowing for the estimation of the magnitude and direction of the relationship between the dependent variable and each independent variable. This coefficient represents the amount by which the dependent variable (volume in mL) changes when the independent variable increases by one unit (years for age, cm for height and kg/m^2^ for BMI, the severity scale MILD or SEV for COVID-19), while keeping all other parameters unchanged. Further parameters included the corresponding standard error, t-value, *p*-value for the respective coefficient, *p*-values and R-squared values for the multivariate model. 

## 3. Results

Table 2 summarised the general demographic and clinical characteristics of the cohort. There were no significant differences between the three sub-cohorts in terms of age, gender distribution, weight, height and BMI. However, on average, the SEV group was older and somewhat heavier than those in the CTL or MILD groups. The time from infection to study inclusion differed from 8.7 ± 4.8 months for ASY to 10.7 ± 5 months for SEV at the time of assessment. Most patients in the SEV group had presented to a normal ward or monitoring unit, whereas 9 out of 48 patients (19%) from the SEV group were admitted to the intensive care unit (ICU).

### Volumetric Brain Analysis

**Figure 1:**
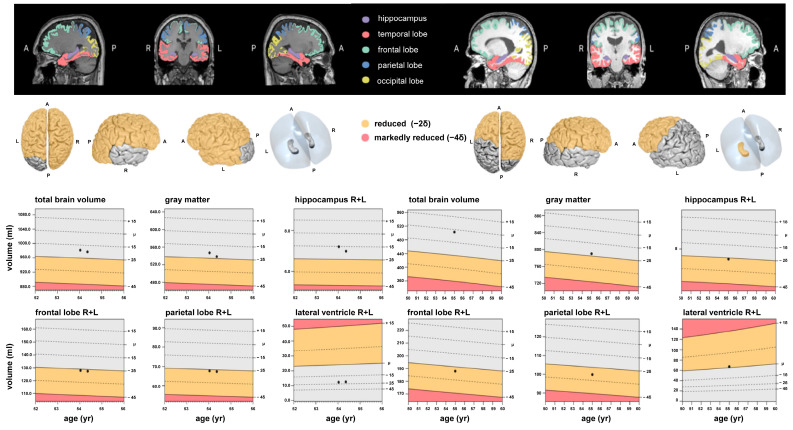
Examples of fully automated, artificial intelligence (AI)-based brain volumetry in patients with severe (**left**) and mild (**right**) cases of COVID-19, showing various brain volumes, along with the deviations of all volumes from a normative collective. These deviations are reported as either 2 or 4 standard deviations.

No imaging findings in the visual clinical assessment led to exclusion of subjects according to the aforementioned exclusion criteria. The volumetry software successfully processed all MR imaging studies, and the measurement results of brain area and ventricle volumes in mL and the corresponding percentiles for the three participant groups CTL, MILD and SEV are shown in Table 3 and Table 4. The mean measured brain volumes of the CTL and MILD groups differed greatly from those of the SEV group. However, the mean measured volumes of the MILD group were of a similar magnitude or slightly higher than those of the CTL group. Exemplary MRI volumetry results are shown in Figure 1.

Comparing all COVID-19 recovered patients (MILD + SEV) with healthy controls, statistically significant smaller volumes in COVID-19 recovered patients were identified for the brainstem volume (74.22 mL vs. 84.68 mL, *p* = 0.046). Smaller percentiles were detected in the supratentorial grey matter (*p* = 0.042), frontal lobe right (*p* = 0.013) and the parietal lobe right (*p* = 0.03) and left (*p* = 0.023).

One-way ANOVA analyses across the three groups (CTL, MILD, SEV) revealed statistically significant differences in several brain volumes (see also Supplementary Table S1): whole brain, whole brain grey matter, supratentorial grey matter, frontal lobe right and left, parietal lobe right and left, precuneus right and left, occipital lobe left, temporal lobe right and left, caudate nucleus right and left, putamen right and left, pallidum right and left, thalamus right and left and cerebellar grey matter. Statistically significant differences in brain percentiles were detected in whole brain grey matter, frontal lobe right, caudate nucleus right and left and thalamus right and left.

Post hoc pairwise analyses showed statistically significant differences with increasing volume decline with disease severity (SEV > MILD > CTL) in the following brain areas, as additionally outlined in Supplementary Table S1: whole brain grey matter, supratentorial grey matter, both frontal lobes, both parietal lobes and thalamus right. Further statistically significant pairwise differences in brain volume were found between SEV and MILD, but not between SEV and CTL, in the following areas: whole brain, precuneus right and left, both temporal lobes, parahippocampal gyrus left, both caudate nuclei, putamen right and left, pallidum right and left, and cerebellar grey matter. Arithmetic differences in these brain volumes were also found between the SEV and CTL groups. The SEV group generally had smaller brain subvolumes than the CTL group, but this was not statistically significant in these areas. Regarding brain percentiles, statistically significant differences were detected between the SEV and CTL group in the whole brain, whole brain grey matter, frontal lobe right, caudate nucleus left and thalamus left. Statistically significant differences were observed in brain area percentiles between the SEV and MILD groups in the whole brain white matter, caudate nucleus left and third ventricle.

After excluding the ICU-treated patients (n = 9) from the data analysis, additional pairwise post hoc comparison between the three groups revealed statistically significant decreases in brain volumes that corresponded with increasing severity of disease (SEV > MILD > CTL). This is summarised in Supplementary Table S2, showing the whole brain grey matter, supratentorial grey matter, frontal lobe right, both parietal lobes and thalamus right.

**Figure 2:**
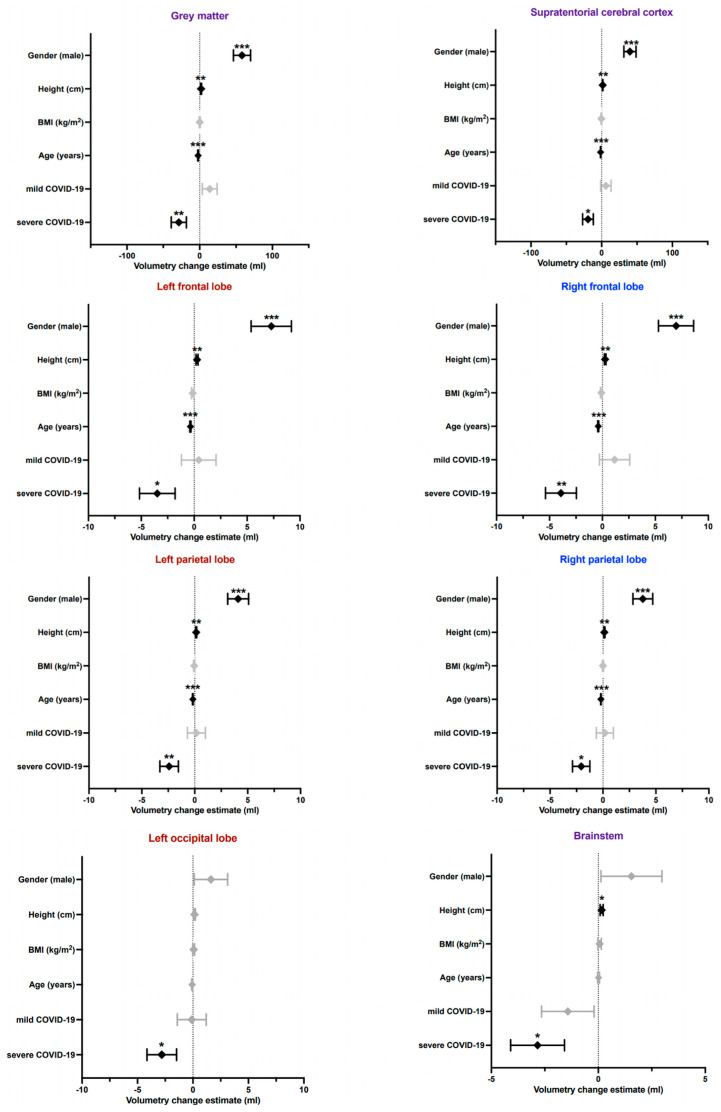
Graphically illustrated results of multivariate modelling in selected brain areas showing the amount by which the dependent variable (volume in mL of a certain brain area) changes when the independent variable increases by one unit (years for age, cm for height and kg/m^2^ for BMI, severity MILD or SEV for COVID-19). Unilateral or whole brain structures are labelled purple, whereas areas on the left are red, and those on the right are blue. *= *p* ≤ 0.05, **= *p* ≤ 0.01, ***= *p* ≤ 0.001.

In addition to established determinants of brain volume such as age and sex, the BMI, height and COVID-19 severity (MILD and SEV) were co-analysed as variables in a multivariate model, yielding statistically significant effects of COVID-19 on brain volume decline in the following areas (Table 5; a complete list of results are shown in Supplementary Table S3): whole brain grey matter, supratentorial grey matter, both frontal lobes, both parietal lobes, precuneus right, occipital lobe left, thalamus right and brainstem. The estimated impact of the respective variables on the selected brain volumes is graphically illustrated in Figure 2. Regarding brain percentiles, a statistically significant contribution of severe COVID-19 to the whole brain volume could be detected.

## 4. Discussion

This MR imaging study evaluated the potential impact of COVID-19 on brain volume in patients after recovery from asymptomatic/mild and severe SARS-CoV-2 infection using automated AI-based volumetry. To date, this study includes the largest number of severely affected COVID-19 patients compared to previously published imaging studies, to the best of our knowledge. Our volumetric analyses revealed small but statistically significant differences in measured brain volumes according to COVID-19 severity. These atrophy patterns primarily affected the total and supratentorial grey matter, both frontal and parietal lobes, and the right thalamus. These findings were further supported by reduced percentiles normalised across the general population in the corresponding brain areas. Notably, the observed group differences were significant even after excluding 9 ICU-treated patients (except for the left frontal lobe). This implies that the potential influence of relaxation, mechanical ventilation or intensified drug therapy on the overall outcome appears largely negligible [12,13]. Moreover, multivariate modelling showed that the severity of COVID-19 had a modest yet statistically significant impact on the measured brain volumes, along with established demographic factors such as age and sex, even after adjustment for ICU admission. Our data, therefore, highlight possible neocortical damage as a sequelae of COVID-19 that could be related to initial disease severity. However, these results likely reflect a cross-sectional effect on COVID-19 recovered individuals, and may not be generalisable, as not all participants exhibited brain changes in the post hoc setting examined here; this is also unlikely to be the case in our upcoming longitudinal studies. Therefore, future studies need to specify the underlying pathologic conditions that may cause severe brain involvement. Our findings may nevertheless be of significant rehabilitative and socioeconomic importance, given the association of brain atrophy with neurodegenerative diseases.

Previous imaging studies have mainly focused on gross CNS abnormalities in acute and hospitalised COVID-19 patients that could only be interpreted by visual assessment [9]. However, most of these studies showed no specific imaging findings or typical spatial distribution in most patients, except for some case series with clusters of white matter lesions or microbleeds within the middle and posterior cerebral artery territory and basal ganglia [14]. Therefore, it is conceivable that COVID-19 mainly causes microstructural damage, as suggested by the fact that macroscopic changes were much less common than microscopic changes in neuropathological case reports [14]. In contrast, our study relied on a fully automated, quantitative, and objective assessment of spatial clusters of brain volume abnormalities. This approach detected visually inconspicuous findings and potentially demonstrated the impact of COVID-19 on brain integrity, unlike most previous imaging studies. To date, only one prospective longitudinal imaging study by Douaud et al. has examined 401 subjects with a mainly mild course, both before and after SARS-CoV-2 infection, and compared them to matched controls using quantitative imaging biomarkers [15]. In a hypothesis-driven and exploratory approach, atrophy patterns were identified in the olfactory and gustatory cortical systems, with longitudinal reductions in grey matter thickness in the left parahippocampal gyrus, left superior (dorsal) insula, and left lateral orbitofrontal cortex, and marked widespread differences in fronto-parietal areas, particularly in the left hemisphere. Although pronounced atrophy was restricted to a few limbic areas, an increase in cerebrospinal fluid volume and a decrease in total brain volume indicated additional diffuse grey matter loss superimposed on the more regional effects observed in olfactory areas. Even though our automated volume measurements were performed on partially larger brain substructures and thus incorporated averaging effects, the results obtained by Douaud et al. are consistent with the atrophy pattern of the fronto-parietal brain observed in our study, and the volume decrease in the temporal lobes, including the gyri hippocampales, accentuated in severely affected patients. Therefore, we can confirm and extend those previous findings given our relatively large number of severely affected patients (n = 48); in severe cases, there may be increased brain damage in the form of atrophy of the whole-brain grey matter. This pattern of grey matter loss is also consistent with findings from two recent ^18^F-FDG-PET studies, which reported a decrease in glucose uptake in the bilateral fronto-parietal regions of hospitalised patients during the subacute stage of COVID-19 [16]. Additionally, bilateral hypometabolism in the orbital gyrus rectus and right medial temporal lobe was observed in patients who had recovered from COVID-19 [17]. A recent post hoc CT-based volumetric study found no significant differences between acutely hospitalised COVID-19 patients and control subjects, but did identify reduced grey matter volume in the frontal regions [18]. Similarly, a post-infection MR imaging study of 51 previously hospitalised COVID-19 patients revealed subtle abnormalities in terms of prolonged thalamic T2* relaxation times compared with matched controls, especially in the right thalamus, and more commonly in those with milder courses [19]. While we also observed thalamic atrophy in mild and severe COVID-19 courses, especially in the right thalamus, interpretation of the possible correlations of atrophy and T2* signal would be rather speculative. However, it is important to note that changes in microvascularity can alter T2* signalling, and microvascular injury and resulting inflammation may have altered T2* in the thalamus. A dysregulated inflammatory response in COVID-19 patients may be related to subsequent atrophy in the dependent volume [20]. Another recent study of 33 hospitalized COVID-19 cases with neurological impairment [21] also found lower cortical volume in the orbitofrontal, frontal, and cingulate areas in COVID-19 patients compared with healthy subjects, similar to our observations. At the beginning of the pandemic, MRI studies of ICU patients revealed signal abnormalities in the grey matter, particularly the hippocampus, frontal lobe, and insula [22], which align with our findings of lower grey matter volume, particularly in the orbitofrontal cortex, compared to controls. This is not surprising, given that this cortical area serves as a secondary olfactory cortex and may provide a potential direct pathway for SARS-CoV-2 to invade the CNS [23]. 

Many COVID-19 patients requiring hospitalisation present with mild to moderate neurocognitive deficits [24]. Dedicated neuropsychological testing has shown that hospitalised COVID-19 patients tend to experience the most severe deficits in memory and executive functions. In contrast, their language skills, orientation, general attention, and processing speed are typically mildly affected [10,16]. These specific patterns suggest that general deterioration or fatigue cannot be the plausible cause of these abnormalities. This is particularly evident given that these findings differ from those in post-septic patients, who typically experience impaired attention and processing speed [25,26]. Instead, our findings suggest the involvement of the fronto-parietal cortex, which aligns with the atrophy pattern in the fronto-parietal brain identified in our study by functional neurocognitive results [27]. In particular, the orbitofrontal and cingulate cortex play essential roles in several cognitive functions such as attention, motivation, decision making, and conflict-error monitoring, which are impaired in COVID-19 patients [28,29] and have been altered in our and other recent studies [15,21]. Moreover, some studies suggest that atrophy of the right thalamus, as observed in our study, is associated with cognitive deficits, including impairments in memory and attention [30].

The pathomechanisms of the acute and long-term neurologic damage resulting from COVID-19 remain largely unclear. However, secondary immune-mediated inflammatory complications [31] are discussed as triggers of neurologic and neurocognitive dysfunction in addition to direct viral invasion [32], given the known neurotropism of the coronavirus [33,34]. Functional PET studies have shown that neocortical damage might not result from persistent encephalitis or systemically triggered local inflammation [16,17]. One hypothesis is that SARS-CoV-2 enters the central nervous system via the olfactory mucosa and directly affects neurons [35]. The symptoms of hyposmia and hypogeusia often precede the full onset of the disease [8], and the neurons in the olfactory and gustatory networks show volumetrically pronounced atrophy [15,17,18,19], providing support for this idea. A study of olfactory loss, both congenital and acquired, found a positive correlation between grey matter volume in the orbitofrontal cortex and olfactory function [36]. Another hypothesis is that hypoxic brain injury may be responsible for the observed brain changes [14]. This is consistent with the finding that chronic under-supply of oxygen, as seen in patients with advanced chronic obstructive pulmonary diseases, causes a reduction in grey matter in widespread regions such as the frontal cortex, cingulate cortex, and other subcortical regions [37]. However, recent studies have identified structural brain changes in asymptomatic COVID-19 patients, as described in our own and other studies [10,15,19]. This makes a hypoxic aetiology of the observed changes less plausible, despite reports of individual cases of restitution after hypoxic brain damage [38].

Several limitations of this exploratory study must be acknowledged. Cross-sectional comparisons were made between healthy controls and COVID-19 patients who had already recovered. Accordingly, it cannot be determined with certainty whether relevant brain changes in COVID-19 patients existed before SARS-CoV-2 infection. The observed effects could ultimately be related to a pre-existing increased susceptibility of the brain to the effects of COVID-19, or therapeutic procedures. However, a recent large-scale pre-post imaging study supports our findings through the substantial overlap with the longitudinally assessed brain atrophies [15]. Furthermore, because our study was observational and not a controlled interventional study, the causality of the observed brain volume changes, in general, cannot be attributed with certainty to COVID-19. The cohort acquisition took place during the ongoing coronavirus pandemic, which saw the emergence of other viral variants such as Delta and Omicron in addition to the wild-type coronavirus. This raises the question whether the observed brain changes could be an expression of a specific strain of the virus. It is well known that patients admitted to the ICU and survivors of critical illness often exhibit neuropsychological and brain changes, including atrophies particularly of the basal ganglia and hippocampi [12,13,26,39]. Nevertheless, our results indicate that these changes do not merely reflect post-ICU effects, as we observed differences in non-hospitalised patients, and even after excluding ICU-patients. Finally, the use of higher resolution volumetry (i.e., segmentation and measurement of finer brain substructures) could potentially reveal further or even greater differences between subcohorts. However, this was not possible with our CE-marked commercial software solution. The use of an in-house built software solution with refined segmentation would have the major disadvantage of complicating comparisons with respect to multicentre research and clinical follow-up.

## 5. Conclusions

We identified a consistent spatial pattern of grey matter loss and focal atrophy in COVID-19 recovered patients, particularly in the frontal and parietal lobes and the right thalamus. These structural changes in the brain are broadly consistent with preliminary volumetric observations of grey matter loss, and were more pronounced in patients with greater disease severity of SARS-CoV-2 infection, regardless of prior ICU treatment. This strongly suggests a causal relationship between COVID-19 and the observed brain changes. Notably, our volumetric results were additionally substantiated by a concomitant decline in percentiles in the corresponding brain areas, setting our study apart from most previous imaging studies. This means that the measured brain volume differences of post-COVID-19 subjects not only exist in absolute numbers compared to our matched control and patient groups, but that the differences also increase with respect to the age- and sex-adjusted general population, with increasing severity of the initial disease. Whether these abnormal changes are due to the spread of the disease or the virus itself, which may result in future vulnerability to neurocognitive deficits or exacerbation of pre-existing neurodegenerative conditions in these patients, remains to be investigated. Our ongoing prospective re-imaging study promises to provide further insight into the cerebral effects of COVID-19, pending completion of the clinical and imaging follow-ups. To this end, our longitudinal investigation will specifically pursue two goals in the near future. Firstly, we aim to correlate the volumetric data with clinical neurologic, pneumonologic, and neuropsychologic findings to verify the potential clinical significance of the observed brain changes. Secondly, pending follow-ups may reveal whether there is a dynamic of brain atrophy over time, which might indicate the triggering of a chronic neurodegenerative process.

## Figures and Tables

**Table 1:** Sequence parameters.

Sequence	Pulse Type	Orientation	TR (ms)	TE (ms)	Reconstructed Voxel Size (mm)	Matrix (mm)	Slices
T2w	Turbo spin echo	axial	13.257	90	0.94 × 0.94 × 1	240 × 174	140
SWI	3D fast field echo	axial	31	0	0.6 × 0.6 × 2	384 × 316	145
DWI	*b* values (0, 500, 1000 s/mm^2^)	axial	2725	41	1 × 1 × 5	128 × 127	24
T1w	MPRAGE	sagittal	7.3	3.9	1 × 1 × 1	256 × 256	180
FLAIR	3D gradient echo	sagittal	4800	275	1.12 × 1.12 × 1.12	240 × 240	321

DWI: diffusion-weighted imaging, MPRAGE: magnetisation prepared-rapid gradient echo, SWI: susceptibility-weighted imaging, T1W: T1-weighted, T2W: T2-weighted, TE: echo time, TR: repetition time.

**Table 2:** Patient demographics and characteristics.

Characteristics	Healthy Control Subjects (CTL)	Mild COVID-19 Course (MILD)	Severe COVID-19 Course (SEV)	Total	*p*-Value ^a^
n	56	51	48	155	
Age (years)	47.0 ± 13.3	45.7 ± 12.4	50.6 ± 12.0	47.7 ± 12.7	0.612
Gender (m:f)	26:25	28:28	25:23	78:76	0.775
Height (mm)	175.0 ± 10.5	173.6 ± 10.7	172.8 ± 10.0	173.8 ± 9.9	0.316
Weight (kg)	79.6 ± 16.2	81.8 ± 23.5	84.4 ± 20.7	81.8 ± 20.2	0.315
BMI	25.9 ± 4.3	27.2 ± 9.1	27.9 ± 6.1	27 ± 6.7	0.154

^a^ calculated with Kruskal–Wallis test; statistical significance set at *p* ≤ 0.05.

**Table 3:** Absolute brain area and ventricle volumes (mL).

Brain Region	CTL		MILD		SEV		
	Mean	±SD	Mean	±SD	Mean	±SD	*p*-Value ^a^
Whole brain	1264.18	117.28	1298.19	143.63	1210.70	113.49	0.003
Whole brain white substance	554.24	61.31	571.79	74.26	537.20	57.18	0.32
Whole brain grey substance	709.83	64.35	726.87	79.03	670.34	71.03	≤0.001
Supratentorial gross cerebral cortex	483.68	46.01	491.17	58.25	455.79	48.26	0.002
Frontal right	91.28	9.72	92.81	11.20	85.32	9.37	0.001
Frontal left	87.52	9.05	88.22	13.73	82.05	9.23	0.01
Parietal right	48.09	5.12	48.53	5.87	45.10	5.40	0.004
Parietal left	50.29	5.44	50.63	6.01	46.91	5.52	0.002
Precuneus right	11.44	1.45	11.66	1.60	10.77	1.36	0.008
Precuneus left	11.91	2.05	12.16	1.76	11.21	1.72	0.032
Occipital right	31.52	3.38	33.27	9.40	30.26	5.51	0.073
Occipital left	37.83	8.09	37.79	7.32	34.61	4.67	0.031
Temporal right	71.60	7.20	73.83	9.20	68.49	7.44	0.005
Temporal left	66.46	8.31	68.20	9.64	63.99	6.73	0.045
Mesiotemporal right	23.86	11.00	26.28	10.79	23.91	10.24	0.423
Mesiotemporal left	22.44	10.23	24.67	9.77	22.40	9.34	0.402
Hippocampus right	5.02	5.87	4.35	0.53	4.17	0.42	0.43
Hippocampus left	5.54	10.69	4.87	3.79	4.12	0.58	0.569
Gyrus parahippocampalis right	3.27	0.37	3.40	0.44	3.22	0.33	0.053
Gyrus parahippocampalis left	3.41	0.33	3.55	0.37	3.32	0.33	0.005
Regio entorhinalis right	2.51	0.31	2.60	0.32	2.47	0.24	0.097
Regio entorhinalis left	2.45	0.28	2.52	0.31	2.42	0.30	0.27
Nucleus caudatus right	3.30	0.43	3.49	0.40	3.19	0.43	0.002
Nucleus caudatus left	2.93	0.38	3.15	0.35	2.84	0.40	≤0.001
Putamen right	4.19	0.46	4.33	0.51	4.02	0.48	0.006
Putamen left	4.31	0.47	4.45	0.55	4.11	0.46	0.004
Pallidum right	1.43	0.17	1.47	0.15	1.38	0.14	0.014
Pallidum left	1.38	0.15	1.44	0.16	1.34	0.15	0.005
Thalamus right	8.13	0.76	8.35	0.81	7.66	0.77	≤0.001
Thalamus left	8.46	0.81	8.46	1.35	7.99	0.78	0.031
Brainstem	27.87	10.31	26.39	2.92	24.88	2.62	0.072
Mesencephalon	8.91	12.29	7.23	0.90	6.81	0.84	0.307
Pons	15.86	11.18	14.36	1.77	13.59	1.47	0.228
Cerebellar grey matter	109.62	25.60	112.52	11.18	105.98	11.48	0.017
Left ventricle	10.09	7.46	8.51	5.92	8.83	5.14	0.389
Right ventricle	9.91	6.21	9.01	5.55	9.56	5.44	0.723
Third ventricle	0.69	0.41	0.76	0.39	0.78	0.35	0.423
Fourth ventricle	1.16	0.37	1.20	0.42	1.10	0.36	0.378

^a^ calculated with one-way analysis of variance; statistical significance set at *p* ≤ 0.05.

**Table 4:** Percentiles of brain areas and ventricle volumes.

Brain Region	CTL		MILD		SEV		
	Mean	±SD	Mean	±SD	Mean	±SD	*p*-Value ^a^
Whole brain	82.09	17.40	80.56	21.51	71.09	27.13	0.029
Whole brain white substance	85.82	16.02	85.84	16.67	82.55	24.63	0.619
Whole brain grey substance	56.14	24.23	53.18	26.60	43.65	26.24	0.041
Supratentorial gross cerebral cortex	46.49	25.98	40.35	26.02	35.05	26.77	0.088
Frontal right	55.31	26.81	46.84	27.67	39.85	27.97	0.018
Frontal left	48.41	25.53	43.48	27.30	35.83	27.84	0.061
Parietal right	34.16	21.89	26.27	21.09	28.87	24.10	0.178
Parietal left	46.44	25.26	36.91	23.87	36.26	26.09	0.066
Precuneus right	47.78	29.91	45.68	26.00	42.65	26.69	0.641
Precuneus left	70.30	23.34	64.93	25.74	60.31	31.78	0.171
Occipital right	23.70	24.08	21.92	23.72	19.84	23.36	0.711
Occipital left	40.51	27.27	38.06	28.98	34.79	28.45	0.589
Temporal right	56.31	28.07	57.84	27.54	51.79	28.19	0.536
Temporal left	56.83	30.13	59.44	27.70	49.30	28.46	0.197
Mesiotemporal right	48.95	32.89	53.54	28.83	48.34	32.17	0.66
Mesiotemporal left	48.21	33.66	52.61	28.19	48.02	31.52	0.703
Hippocampus right	56.14	26.79	57.43	28.65	56.09	26.77	0.962
Hippocampus left	58.86	26.41	59.19	27.21	58.84	24.98	0.997
Gyrus parahippocampalis right	62.83	28.41	69.88	28.61	67.68	22.35	0.378
Gyrus parahippocampalis left	60.66	27.73	71.51	23.27	60.91	26.97	0.058
Regio entorhinalis right	70.46	26.90	75.37	21.56	72.13	19.83	0.543
Regio entorhinalis left	59.11	26.74	63.99	26.23	58.62	26.94	0.532
Nucleus caudatus right	40.50	25.82	52.19	24.97	40.37	27.25	0.033
Nucleus caudatus left	27.37	22.08	39.51	23.31	27.90	24.42	≤0.001
Putamen right	27.69	22.36	31.95	24.28	25.74	25.12	0.415
Putamen left	29.71	24.42	33.84	25.37	25.11	22.97	0.207
Pallidum right	36.65	27.64	40.73	25.44	36.08	29.20	0.648
Pallidum left	27.09	23.85	28.51	22.19	25.08	25.64	0.773
Thalamus right	44.43	30.43	44.14	28.17	31.93	27.93	0.052
Thalamus left	56.73	29.84	54.33	26.68	42.83	29.39	0.037
Brainstem	55.91	24.97	56.35	28.32	46.25	26.65	0.106
Mesencephalon	49.42	27.37	49.82	28.56	40.90	27.23	0.198
Pons	54.01	27.01	51.15	29.24	44.37	27.23	0.204
Cerebellar grey matter	68.01	24.56	70.45	25.60	60.24	27.65	0.127
Left ventricle	54.37	31.06	48.48	29.31	56.07	27.71	0.401
Right ventricle	54.67	31.64	51.08	29.46	56.61	29.85	0.723
Third ventricle	48.64	29.01	43.47	29.44	59.20	27.78	0.024
Fourth ventricle	48.34	28.67	54.38	27.16	48.82	30.70	0.498

^a^ calculated with one-way analysis of variance; statistical significance set at *p* ≤ 0.05.

**Table 5:** Multivariate modelling in selected brain regions with volumetric changes following severe COVID-19 infection.

Brain Region		Estimate	Standard Error	t-Value	*p*-Value
Whole brain	Age (years)	−2.4107	0.6102	−3.951	<0.001
	Gender (male)	97.1842	21.3302	4.556	<0.001
	COVID-19 Mild	32.6959	18.3726	1.780	0.077195
	COVID-19 Severe	−36.1273	18.8432	−1.917	0.057131
	Height	4.0755	1.0574	3.854	<0.001
	BMI	−0.9211	1.1922	−0.773	0.441023
	*Multiple R-squared*	0.4932		*p-value*	<0.001
Grey matter	Age (years)	−2.20669	0.33652	−6.557	<0.001
	Gender (male)	58.22570	11.76448	4.949	<0.001
	COVID-19 Mild	13.84293	10.13324	1.366	0.17398
	COVID-19 Severe	−28.64409	10.39277	−2.756	0.00658
	Height	1.92446	0.58321	3.300	0.00121
	BMI	0.05646	0.65757	0.086	0.93169
	*Multiple R-squared*	0.5367		*p-value*	<0.001
Supratentorial	Age (years)	−1.53934	0.24475	−6.289	<0.001
cerebral	Gender (male)	39.97339	8.55623	4.671	<0.001
cortex	COVID-19 Mild	5.92185	7.36984	0.803	0.42295
	COVID-19 Severe	−19.36262	7.55859	−2.561	0.01141
	Height	1.37947	0.42416	3.252	0.00141
	BMI	−0.41309	0.47824	−0.863	0.38911
	*Multiple R-squared*	0.5097		*p-value*	<0.001
Frontal lobe	Age (years)	−0.39890	0.04735	−8.425	<0.001
right	Gender (male)	6.96303	1.65526	4.207	<0.001
	COVID-19 Mild	1.15013	1.42575	0.807	0.42114
	COVID-19 Severe	−3.92092	1.46226	−2.681	0.00816
	Height	0.25741	0.08206	3.137	0.00206
	BMI	−0.09647	0.09252	−1.043	0.29881
	*Multiple R-squared*	0.5397		*p-value*	<0.001
Frontal lobe	Age (years)	−0.35550	0.05445	−6.529	<0.001
left	Gender (male)	7.28431	1.90364	3.827	<0.001
	COVID-19 Mild	0.42527	1.63969	0.259	0.795716
	COVID-19 Severe	−3.48425	1.68168	−2.072	0.040011
	Height	0.27121	0.09437	2.874	0.004651
	BMI	−0.13552	0.10640	−1.274	0.204797
	*Multiple R-squared*	0.4524		*p-value*	<0.001
Parietal lobe	Age (years)	−0.19783	0.02668	−7.414	<0.001
right	Gender (male)	3.76298	0.93281	4.034	<0.001
	COVID-19 Mild	0.17747	0.80347	0.221	0.8255
	COVID-19 Severe	−2.05789	0.82405	−2.497	0.0136
	Height	0.13298	0.04624	2.876	0.0046
	BMI	−0.00355	0.05214	−0.068	0.9458
	*Multiple R-squared*	0.4866		*p-value*	<0.001
Parietal lobe	Age (years)	−0.18253	0.02821	−6.47	<0.001
left	Gender (male)	4.09906	0.98632	4.156	<0.001
	COVID-19 Mild	0.15045	0.84956	0.177	0.8597
	COVID-19 Severe	−2.4184	0.87132	−2.776	0.0062
	Height	0.13495	0.0489	2.76	0.0065
	BMI	−0.048	0.05513	−0.871	0.3853
	*Multiple R-squared*	0.4701		*p-value*	<0.001
Precuneus	Age (years)	−0.0379	0.00757	−5.01	<0.001
right	Gender (male)	1.1321	0.26447	4.281	<0.001
	COVID-19 Mild	0.16744	0.2278	0.735	0.4635
	COVID-19 Severe	−0.4825	0.23363	−2.065	0.0406
	Height	0.03398	0.01311	2.592	0.0105
	BMI	0.00059	0.01478	0.04	0.9682
	*Multiple R-squared*	0.4255		*p-value*	<0.001
Occipital lobe	Age (years)	−0.07167	0.04324	−1.658	0.0995
left	Gender (male)	1.62171	1.51154	1.073	0.2851
	COVID-19 Mild	−0.10926	1.30195	−0.084	0.9332
	COVID-19 Severe	−2.81361	1.3353	−2.107	0.0368
	Height	0.14092	0.07493	1.881	0.062
	BMI	0.05991	0.08449	0.709	0.4794
	*Multiple R-squared*	0.1398		*p-value*	<0.001
Thalamus	Age (years)	−0.02283	0.00425	−5.375	<0.001
right	Gender (male)	0.3445	0.14851	2.32	0.0217
	COVID-19 Mild	0.20392	0.12792	1.594	0.113
	COVID-19 Severe	−0.33729	0.13119	−2.571	0.0111
	Height	0.0214	0.00736	2.906	0.0042
	BMI	−0.00289	0.0083	−0.348	0.7284
	*Multiple R-squared*	0.3903		*p-value*	<0.001
Brainstem	Age (years)	0.0099	0.04076	0.243	0.8083
	Gender (male)	1.54783	1.4248	1.086	0.2791
	COVID-19 Mild	−1.4237	1.22724	−1.16	0.2479
	COVID-19 Severe	−2.84074	1.25867	−2.257	0.0255
	Height	0.15566	0.07063	2.204	0.0291
	BMI	0.05938	0.07964	0.746	0.4571
	*Multiple R-squared*	0.1434		*p-value*	<0.001

## Data Availability

All relevant scientific data are included in the manuscript or the Supplementary Materials.

## Supplementary Materials

The following supporting information can be downloaded at: https://www.mdpi.com/article/10.3390/diagnostics13101716/s1, Table S1: pairwise analyses; Table S2: pairwise analyses ex ICU; Table S3: multivariate analysis.
