# Supplementary material for: Brain Volume Changes after COVID-19 Compared to Healthy Controls by Artificial Intelligence-Based MRI Volumetry
Source: Diagnostics (Basel). 2023 May 12;13(10):1716. doi: 10.3390/diagnostics13101716 (PMC10216908; doi:10.3390/diagnostics13101716)
Supplement: Supplementary file 1 [file diagnostics-13-01716-s001.zip › Supplemental Table S1.doc]

Table S1. Pairwise analyses


Descriptives	
	N	Mean	Std. Deviation	Std. Error	95% Confidence Interval for Mean	Minimum	Maximum	
					Lower Bound	Upper Bound			
Whole_brain_volume	Control Group	56	1264,1768	117,28260	15,67255	1232,7683	1295,5853	1007,00	1551,20	
	Asymptomatic non-hospitalised COVID-19	51	1298,1882	143,62517	20,11155	1257,7930	1338,5835	1018,00	1626,60	
	Severe hospitalised COVID-19	48	1210,7042	113,48581	16,38027	1177,7513	1243,6570	952,00	1452,00	
	Total	155	1258,8084	129,56061	10,40656	1238,2504	1279,3664	952,00	1626,60	
Whole_brain_precentile	Control Group	56	82,0929	17,40070	2,32527	77,4329	86,7528	27,30	99,40	
	Asymptomatic non-hospitalised COVID-19	51	80,5608	21,50755	3,01166	74,5117	86,6099	16,00	100,00	
	Severe hospitalised COVID-19	48	71,0896	27,12946	3,91580	63,2120	78,9672	6,60	100,00	
	Total	155	78,1813	22,49587	1,80691	74,6118	81,7508	6,60	100,00	
Whole_brain_white_substance	Control Group	56	554,2429	61,30981	8,19287	537,8240	570,6617	400,00	729,90	
	Asymptomatic non-hospitalised COVID-19	51	571,7882	74,26375	10,39900	550,9012	592,6753	440,00	773,30	
	Severe hospitalised COVID-19	48	537,1958	57,17576	8,25261	520,5937	553,7979	382,00	649,00	
	Total	155	554,7368	65,74999	5,28117	544,3039	565,1697	382,00	773,30	
Whole_brain_white_substance_percentile	Control Group	56	85,8214	16,02419	2,14132	81,5301	90,1127	33,00	100,00	
	Asymptomatic non-hospitalised COVID-19	51	85,8431	16,67326	2,33472	81,1537	90,5326	36,40	100,00	
	Severe hospitalised COVID-19	48	82,5479	24,62661	3,55454	75,3971	89,6987	10,50	100,00	
	Total	155	84,8148	19,21916	1,54372	81,7652	87,8644	10,50	100,00	
Whole_brain_grey_substance	Control Group	56	709,8250	64,35361	8,59961	692,5910	727,0590	603,20	888,00	
	Asymptomatic non-hospitalised COVID-19	51	726,8706	79,02982	11,06639	704,6431	749,0981	579,00	896,00	
	Severe hospitalised COVID-19	48	670,3396	71,03381	10,25285	649,7135	690,9657	450,00	803,00	
	Total	155	703,2058	74,73235	6,00265	691,3476	715,0640	450,00	896,00	
Whole_brain_grey_substance_percentile	Control Group	56	56,1375	24,22742	3,23753	49,6494	62,6256	3,00	98,00	
	Asymptomatic non-hospitalised COVID-19	51	53,1824	26,60188	3,72501	45,7005	60,6643	4,00	99,40	
	Severe hospitalised COVID-19	48	43,6479	26,24403	3,78800	36,0274	51,2684	3,00	94,00	
	Total	155	51,2974	26,02936	2,09073	47,1672	55,4276	3,00	99,40	
Supratentorial_gross_cerebral_cortex_volume	Control Group	56	483,6821	46,01472	6,14898	471,3593	496,0050	395,00	604,00	
	Asymptomatic non-hospitalised COVID-19	51	491,1745	58,25246	8,15698	474,7907	507,5583	381,00	611,00	
	Severe hospitalised COVID-19	48	455,7854	48,26263	6,96611	441,7714	469,7994	345,50	556,90	
	Total	155	477,5084	52,83570	4,24387	469,1247	485,8921	345,50	611,00	
Supratentorial_cerebral_cortex_percentile	Control Group	56	46,4929	25,97637	3,47124	39,5363	53,4494	3,00	99,00	
	Asymptomatic non-hospitalised COVID-19	51	40,3471	26,02458	3,64417	33,0275	47,6666	1,60	99,70	
	Severe hospitalised COVID-19	48	35,0521	26,76571	3,86330	27,2801	42,8240	,60	87,00	
	Total	155	40,9277	26,48891	2,12764	36,7246	45,1309	,60	99,70	
Frontal_right_volume	Control Group	56	91,2768	9,71660	1,29844	88,6747	93,8789	69,30	122,00	
	Asymptomatic non-hospitalised COVID-19	51	92,8137	11,20030	1,56836	89,6636	95,9639	69,00	117,00	
	Severe hospitalised COVID-19	48	85,3167	9,36979	1,35241	82,5960	88,0374	65,00	112,00	
	Total	155	89,9368	10,54937	,84735	88,2629	91,6107	65,00	122,00	
Frontal_right_percentile	Control Group	56	55,3071	26,81456	3,58325	48,1262	62,4881	3,00	98,00	
	Asymptomatic non-hospitalised COVID-19	51	46,8412	27,66534	3,87392	39,0602	54,6222	,10	99,80	
	Severe hospitalised COVID-19	48	39,8521	27,96578	4,03651	31,7317	47,9725	2,00	99,10	
	Total	155	47,7355	28,00824	2,24968	43,2913	52,1797	,10	99,80	
Frontal_left_volume	Control Group	56	87,5196	9,04657	1,20890	85,0970	89,9423	70,60	116,00	
	Asymptomatic non-hospitalised COVID-19	51	88,2235	13,72644	1,92209	84,3629	92,0842	31,00	115,00	
	Severe hospitalised COVID-19	48	82,0521	9,22838	1,33200	79,3724	84,7317	62,00	104,40	
	Total	155	86,0581	11,12319	,89344	84,2931	87,8230	31,00	116,00	
Frontal_left_percentile	Control Group	56	48,4071	25,52765	3,41128	41,5708	55,2435	1,00	97,00	
	Asymptomatic non-hospitalised COVID-19	51	43,4804	27,30055	3,82284	35,8020	51,1588	,10	99,30	
	Severe hospitalised COVID-19	48	35,8333	27,84098	4,01850	27,7492	43,9175	1,00	95,00	
	Total	155	42,8923	27,16599	2,18202	38,5817	47,2028	,10	99,30	
Parietal_right_volume	Control Group	56	48,0929	5,12388	,68471	46,7207	49,4650	39,00	60,00	
	Asymptomatic non-hospitalised COVID-19	51	48,5275	5,87156	,82218	46,8760	50,1789	38,00	62,40	
	Severe hospitalised COVID-19	48	45,1042	5,39625	,77888	43,5373	46,6711	33,00	57,00	
	Total	155	47,3103	5,62914	,45214	46,4171	48,2035	33,00	62,40	
Parietal_right_percentile	Control Group	56	34,1643	21,88769	2,92487	28,3027	40,0258	3,00	96,00	
	Asymptomatic non-hospitalised COVID-19	51	26,2667	21,08900	2,95305	20,3353	32,1980	,10	88,40	
	Severe hospitalised COVID-19	48	28,8708	24,09915	3,47841	21,8732	35,8685	,30	89,00	
	Total	155	29,9265	22,45119	1,80332	26,3640	33,4889	,10	96,00	
Parietal_left_volume	Control Group	56	50,2875	5,43765	,72664	48,8313	51,7437	38,00	61,20	
	Asymptomatic non-hospitalised COVID-19	51	50,6314	6,01184	,84183	48,9405	52,3222	39,80	63,00	
	Severe hospitalised COVID-19	48	46,9083	5,52364	,79727	45,3044	48,5122	34,00	59,00	
	Total	155	49,3542	5,85898	,47060	48,4245	50,2839	34,00	63,00	
Parietal_left_percentile	Control Group	56	46,4357	25,25666	3,37506	39,6719	53,1995	5,00	99,00	
	Asymptomatic non-hospitalised COVID-19	51	36,9137	23,87354	3,34296	30,1992	43,6283	,20	89,20	
	Severe hospitalised COVID-19	48	36,2563	26,09294	3,76619	28,6796	43,8329	,60	87,00	
	Total	155	40,1503	25,36177	2,03711	36,1260	44,1746	,20	99,00	
Precuneus_right_volume	Control Group	56	11,4357	1,44555	,19317	11,0486	11,8228	8,40	14,10	
	Asymptomatic non-hospitalised COVID-19	51	11,6627	1,59762	,22371	11,2134	12,1121	8,90	14,80	
	Severe hospitalised COVID-19	48	10,7667	1,35777	,19598	10,3724	11,1609	7,90	13,00	
	Total	155	11,3032	1,50882	,12119	11,0638	11,5426	7,90	14,80	
Precuneus_right_percentile	Control Group	56	47,7804	29,91054	3,99696	39,7703	55,7905	3,00	95,60	
	Asymptomatic non-hospitalised COVID-19	51	45,6784	26,00076	3,64084	38,3656	52,9913	,10	98,50	
	Severe hospitalised COVID-19	48	42,6479	26,68875	3,85219	34,8983	50,3975	,80	90,50	
	Total	155	45,4994	27,58318	2,21553	41,1226	49,8761	,10	98,50	
Precuneus_left_volume	Control Group	56	11,9130	2,04552	,27334	11,3652	12,4608	1,03	14,60	
	Asymptomatic non-hospitalised COVID-19	51	12,1647	1,76009	,24646	11,6697	12,6597	8,90	15,80	
	Severe hospitalised COVID-19	48	11,2083	1,72044	,24832	10,7088	11,7079	7,80	14,70	
	Total	155	11,7776	1,88701	,15157	11,4782	12,0770	1,03	15,80	
Precuneus_left_percentile	Control Group	56	70,2964	23,33737	3,11859	64,0466	76,5462	11,00	99,90	
	Asymptomatic non-hospitalised COVID-19	51	64,9333	25,73612	3,60378	57,6949	72,1717	9,20	99,50	
	Severe hospitalised COVID-19	48	60,3125	31,78197	4,58733	51,0840	69,5410	1,10	99,70	
	Total	155	65,4400	27,10518	2,17714	61,1391	69,7409	1,10	99,90	
Occipital_right_volume	Control Group	56	31,5232	3,38005	,45168	30,6180	32,4284	23,00	39,60	
	Asymptomatic non-hospitalised COVID-19	51	33,2706	9,39728	1,31588	30,6276	35,9136	21,00	74,00	
	Severe hospitalised COVID-19	48	30,2646	5,51217	,79561	28,6640	31,8652	22,00	54,70	
	Total	155	31,7084	6,59511	,52973	30,6619	32,7549	21,00	74,00	
Occipital_right_percentlile	Control Group	56	23,7018	24,08260	3,21817	17,2524	30,1511	,70	92,50	
	Asymptomatic non-hospitalised COVID-19	51	21,9235	23,72335	3,32193	15,2512	28,5958	,00	85,60	
	Severe hospitalised COVID-19	48	19,8417	23,35770	3,37139	13,0593	26,6240	,00	92,20	
	Total	155	21,9213	23,64041	1,89884	18,1701	25,6724	,00	92,50	
Occipital_left_volume	Control Group	56	37,8321	8,09296	1,08147	35,6648	39,9995	26,00	90,00	
	Asymptomatic non-hospitalised COVID-19	51	37,7902	7,32372	1,02553	35,7304	39,8500	25,00	72,00	
	Severe hospitalised COVID-19	48	34,6083	4,66658	,67356	33,2533	35,9634	25,00	45,40	
	Total	155	36,8200	7,04706	,56603	35,7018	37,9382	25,00	90,00	
Occipital_left_percentile	Control Group	56	40,5107	27,27471	3,64474	33,2065	47,8149	2,20	94,60	
	Asymptomatic non-hospitalised COVID-19	51	38,0569	28,97574	4,05741	29,9073	46,2064	,80	95,60	
	Severe hospitalised COVID-19	48	34,7917	28,44889	4,10624	26,5310	43,0524	,80	97,90	
	Total	155	37,9323	28,12082	2,25872	33,4702	42,3943	,80	97,90	
Temporal_right_volume	Control Group	56	71,5964	7,20136	,96232	69,6679	73,5250	57,00	87,00	
	Asymptomatic non-hospitalised COVID-19	51	73,8333	9,19973	1,28822	71,2459	76,4208	59,00	93,00	
	Severe hospitalised COVID-19	48	68,4917	7,44440	1,07451	66,3300	70,6533	49,80	84,00	
	Total	155	71,3710	8,21732	,66003	70,0671	72,6749	49,80	93,00	
Temporal_right_percentile	Control Group	56	56,3125	28,07251	3,75135	48,7946	63,8304	4,00	99,00	
	Asymptomatic non-hospitalised COVID-19	51	57,8431	27,53560	3,85576	50,0986	65,5876	7,30	99,90	
	Severe hospitalised COVID-19	48	51,7854	28,19451	4,06953	43,5986	59,9723	4,00	99,20	
	Total	155	55,4142	27,86726	2,23835	50,9924	59,8360	4,00	99,90	
Temporal_left_volume	Control Group	56	66,4643	8,30854	1,11027	64,2392	68,6893	30,00	83,00	
	Asymptomatic non-hospitalised COVID-19	51	68,1980	9,64155	1,35009	65,4863	70,9098	31,00	87,00	
	Severe hospitalised COVID-19	48	63,9896	6,73436	,97202	62,0341	65,9450	48,50	77,10	
	Total	155	66,2684	8,45828	,67939	64,9263	67,6105	30,00	87,00	
Temporal_left_percentlile	Control Group	56	56,8250	30,13031	4,02633	48,7561	64,8939	3,10	99,00	
	Asymptomatic non-hospitalised COVID-19	51	59,4373	27,70386	3,87932	51,6454	67,2291	7,70	99,90	
	Severe hospitalised COVID-19	48	49,3042	28,46464	4,10852	41,0389	57,5694	2,00	97,80	
	Total	155	55,3555	28,95535	2,32575	50,7610	59,9500	2,00	99,90	
Mesiotemporal_right_volume	Control Group	56	23,8571	11,00096	1,47006	20,9111	26,8032	5,00	38,00	
	Asymptomatic non-hospitalised COVID-19	51	26,2843	10,79100	1,51104	23,2493	29,3193	7,10	40,00	
	Severe hospitalised COVID-19	48	23,9063	10,23736	1,47764	20,9336	26,8789	4,00	38,00	
	Total	155	24,6710	10,69109	,85873	22,9746	26,3674	4,00	40,00	
Mesiotemporal_right_percentile	Control Group	56	48,9482	32,89097	4,39524	40,1400	57,7565	8,00	99,00	
	Asymptomatic non-hospitalised COVID-19	51	53,5353	28,83399	4,03757	45,4256	61,6450	12,00	99,00	
	Severe hospitalised COVID-19	48	48,3417	32,17312	4,64379	38,9996	57,6838	8,00	100,00	
	Total	155	50,2697	31,26552	2,51131	45,3086	55,2307	8,00	100,00	
Mesiotemproal_left_volume	Control Group	56	22,4357	10,23394	1,36757	19,6950	25,1764	5,00	36,00	
	Asymptomatic non-hospitalised COVID-19	51	24,6941	9,79190	1,37114	21,9401	27,4481	8,60	39,00	
	Severe hospitalised COVID-19	48	22,4042	9,33837	1,34788	19,6926	25,1157	4,00	35,10	
	Total	155	23,1690	9,81315	,78821	21,6119	24,7261	4,00	39,00	
Mesiotemporal_left_percentile	Control Group	56	48,2054	33,65593	4,49746	39,1922	57,2185	5,00	99,00	
	Asymptomatic non-hospitalised COVID-19	51	52,6118	28,19284	3,94779	44,6824	60,5411	16,00	99,90	
	Severe hospitalised COVID-19	48	48,0208	31,51513	4,54882	38,8698	57,1719	6,00	99,70	
	Total	155	49,5981	31,14829	2,50189	44,6556	54,5405	5,00	99,90	
Hippocampus_right_volume	Control Group	56	5,0214	5,86576	,78385	3,4506	6,5923	3,50	48,00	
	Asymptomatic non-hospitalised COVID-19	51	4,3529	,52853	,07401	4,2043	4,5016	3,60	6,00	
	Severe hospitalised COVID-19	48	4,1688	,42233	,06096	4,0461	4,2914	3,40	5,00	
	Total	155	4,5374	3,54573	,28480	3,9748	5,1000	3,40	48,00	
Hippocampus_right_percentile	Control Group	56	56,1393	26,79240	3,58028	48,9642	63,3143	1,00	98,60	
	Asymptomatic non-hospitalised COVID-19	51	57,4255	28,65053	4,01188	49,3674	65,4836	,30	99,00	
	Severe hospitalised COVID-19	48	56,0854	26,77043	3,86398	48,3121	63,8587	7,00	95,00	
	Total	155	56,5458	27,23934	2,18792	52,2236	60,8680	,30	99,00	
Hippocampus_left_volume	Control Group	56	5,5429	10,69430	1,42909	2,6789	8,4068	3,20	84,00	
	Asymptomatic non-hospitalised COVID-19	51	4,8686	3,78690	,53027	3,8035	5,9337	3,40	31,00	
	Severe hospitalised COVID-19	48	4,1208	,57574	,08310	3,9537	4,2880	3,10	5,00	
	Total	155	4,8806	6,77808	,54443	3,8051	5,9562	3,10	84,00	
Hippocampus_left_percentile	Control Group	56	58,8643	26,40656	3,52872	51,7926	65,9360	6,80	99,80	
	Asymptomatic non-hospitalised COVID-19	51	59,1882	27,21394	3,81071	51,5342	66,8423	8,30	99,00	
	Severe hospitalised COVID-19	48	58,8375	24,98414	3,60615	51,5829	66,0921	6,70	95,00	
	Total	155	58,9626	26,07723	2,09457	54,8248	63,1004	6,70	99,80	
Gyrus_parahippocampalis_right_volume	Control Group	56	3,2732	,37440	,05003	3,1729	3,3735	2,50	4,10	
	Asymptomatic non-hospitalised COVID-19	51	3,4000	,43726	,06123	3,2770	3,5230	2,50	4,30	
	Severe hospitalised COVID-19	48	3,2167	,33091	,04776	3,1206	3,3128	2,50	4,10	
	Total	155	3,2974	,38896	,03124	3,2357	3,3591	2,50	4,30	
Gyrus_parahippocampalis_right_percentile	Control Group	56	62,8321	28,40811	3,79619	55,2244	70,4399	3,30	99,90	
	Asymptomatic non-hospitalised COVID-19	51	69,8824	28,60626	4,00568	61,8367	77,9280	1,40	99,90	
	Severe hospitalised COVID-19	48	67,6813	22,35340	3,22643	61,1905	74,1720	22,40	99,90	
	Total	155	66,6535	26,74882	2,14852	62,4092	70,8979	1,40	99,90	
Gyrus_parahippocampalis_left_volume	Control Group	56	3,4089	,33044	,04416	3,3204	3,4974	2,60	4,20	
	Asymptomatic non-hospitalised COVID-19	51	3,5490	,36790	,05152	3,4455	3,6525	2,80	4,30	
	Severe hospitalised COVID-19	48	3,3208	,33387	,04819	3,2239	3,4178	2,60	4,10	
	Total	155	3,4277	,35429	,02846	3,3715	3,4840	2,60	4,30	
Gyrus_parahippocampalis_left_percentile	Control Group	56	60,6571	27,73297	3,70597	53,2302	68,0841	6,10	99,80	
	Asymptomatic non-hospitalised COVID-19	51	71,5098	23,26589	3,25788	64,9662	78,0534	18,80	99,90	
	Severe hospitalised COVID-19	48	60,9062	26,96947	3,89271	53,0751	68,7374	3,80	98,70	
	Total	155	64,3052	26,42056	2,12215	60,1129	68,4974	3,80	99,90	
Regio_entorhinalis_right_volume	Control Group	56	2,5054	,31008	,04144	2,4223	2,5884	1,90	3,20	
	Asymptomatic non-hospitalised COVID-19	51	2,5961	,32060	,04489	2,5059	2,6862	2,10	3,30	
	Severe hospitalised COVID-19	48	2,4729	,24122	,03482	2,4029	2,5430	2,00	3,20	
	Total	155	2,5252	,29687	,02384	2,4781	2,5723	1,90	3,30	
Regio_entorhinalis_right_percentile	Control Group	56	70,4643	26,89721	3,59429	63,2612	77,6674	3,90	99,70	
	Asymptomatic non-hospitalised COVID-19	51	75,3706	21,56478	3,01967	69,3054	81,4358	20,50	99,90	
	Severe hospitalised COVID-19	48	72,1333	19,82569	2,86159	66,3766	77,8901	29,00	99,80	
	Total	155	72,5955	23,09964	1,85541	68,9301	76,2608	3,90	99,90	
Regio_entorhinalis_left_volume	Control Group	56	2,4536	,27632	,03692	2,3796	2,5276	1,90	3,30	
	Asymptomatic non-hospitalised COVID-19	51	2,5176	,31222	,04372	2,4298	2,6055	1,60	3,10	
	Severe hospitalised COVID-19	48	2,4229	,30474	,04399	2,3344	2,5114	1,80	3,40	
	Total	155	2,4652	,29796	,02393	2,4179	2,5124	1,60	3,40	
Regio_entorhinalis_left_percentile	Control Group	56	59,1143	26,74015	3,57330	51,9532	66,2753	2,50	99,90	
	Asymptomatic non-hospitalised COVID-19	51	63,9922	26,22516	3,67226	56,6162	71,3681	2,50	97,70	
	Severe hospitalised COVID-19	48	58,6167	26,93636	3,88793	50,7952	66,4382	3,80	99,90	
	Total	155	60,5652	26,56964	2,13413	56,3492	64,7811	2,50	99,90	
Nucleus_caudatus_right_volume	Control Group	56	3,3000	,42725	,05709	3,1856	3,4144	2,40	4,50	
	Asymptomatic non-hospitalised COVID-19	51	3,4882	,39982	,05599	3,3758	3,6007	2,70	4,50	
	Severe hospitalised COVID-19	48	3,1875	,43448	,06271	3,0613	3,3137	2,40	4,20	
	Total	155	3,3271	,43549	,03498	3,2580	3,3962	2,40	4,50	
Nucleus_caudatus_right_percentile	Control Group	56	40,4964	25,81606	3,44982	33,5828	47,4100	2,20	99,40	
	Asymptomatic non-hospitalised COVID-19	51	52,1902	24,97050	3,49657	45,1671	59,2133	4,50	96,60	
	Severe hospitalised COVID-19	48	40,3667	27,25060	3,93329	32,4539	48,2794	3,20	96,20	
	Total	155	44,3039	26,41598	2,12178	40,1123	48,4954	2,20	99,40	
Nucleus_caudatus_left_volume	Control Group	56	2,9268	,37537	,05016	2,8263	3,0273	2,10	4,10	
	Asymptomatic non-hospitalised COVID-19	51	3,1490	,35178	,04926	3,0501	3,2480	2,40	3,80	
	Severe hospitalised COVID-19	48	2,8354	,40132	,05793	2,7189	2,9519	2,00	3,60	
	Total	155	2,9716	,39572	,03178	2,9088	3,0344	2,00	4,10	
Nucleus_caudatus_left_percentile	Control Group	56	27,3679	22,08103	2,95070	21,4545	33,2812	1,00	86,40	
	Asymptomatic non-hospitalised COVID-19	51	39,5078	23,30756	3,26371	32,9525	46,0632	,20	93,50	
	Severe hospitalised COVID-19	48	27,8979	24,42076	3,52483	20,8069	34,9890	,50	84,80	
	Total	155	31,5265	23,74888	1,90756	27,7581	35,2948	,20	93,50	
Putamen_right_volume	Control Group	56	4,1893	,45793	,06119	4,0667	4,3119	3,30	5,30	
	Asymptomatic non-hospitalised COVID-19	51	4,3294	,50884	,07125	4,1863	4,4725	3,10	5,80	
	Severe hospitalised COVID-19	48	4,0167	,47639	,06876	3,8783	4,1550	3,00	5,40	
	Total	155	4,1819	,49392	,03967	4,1036	4,2603	3,00	5,80	
Putamen_right_percentile	Control Group	56	27,6875	22,35835	2,98776	21,6999	33,6751	,10	92,30	
	Asymptomatic non-hospitalised COVID-19	51	31,9510	24,27669	3,39942	25,1231	38,7789	,20	91,80	
	Severe hospitalised COVID-19	48	25,7417	25,12059	3,62585	18,4474	33,0359	,10	95,80	
	Total	155	28,4877	23,85437	1,91603	24,7026	32,2728	,10	95,80	
Putamen_left_volume	Control Group	56	4,3071	,47246	,06314	4,1806	4,4337	3,40	5,50	
	Asymptomatic non-hospitalised COVID-19	51	4,4510	,55113	,07717	4,2960	4,6060	3,20	6,10	
	Severe hospitalised COVID-19	48	4,1146	,46356	,06691	3,9800	4,2492	3,20	5,40	
	Total	155	4,2948	,51204	,04113	4,2136	4,3761	3,20	6,10	
Putamen_left_percentile	Control Group	56	29,7125	24,41752	3,26293	23,1734	36,2516	,10	81,70	
	Asymptomatic non-hospitalised COVID-19	51	33,8353	25,36712	3,55211	26,7007	40,9699	,10	96,60	
	Severe hospitalised COVID-19	48	25,1146	22,96844	3,31521	18,4452	31,7839	,10	92,70	
	Total	155	29,6452	24,39424	1,95939	25,7744	33,5159	,10	96,60	
Pallidum_right_volume	Control Group	56	1,4268	,17108	,02286	1,3810	1,4726	1,10	2,00	
	Asymptomatic non-hospitalised COVID-19	51	1,4725	,15110	,02116	1,4301	1,5150	1,10	1,80	
	Severe hospitalised COVID-19	48	1,3792	,14286	,02062	1,3377	1,4206	1,00	1,70	
	Total	155	1,4271	,15967	,01283	1,4018	1,4524	1,00	2,00	
Pallidum_right_percentile	Control Group	56	36,6536	27,64327	3,69399	29,2507	44,0565	1,30	98,10	
	Asymptomatic non-hospitalised COVID-19	51	40,7314	25,43717	3,56192	33,5771	47,8857	,40	92,40	
	Severe hospitalised COVID-19	48	36,0771	29,20095	4,21479	27,5980	44,5562	,90	97,80	
	Total	155	37,8168	27,34005	2,19601	33,4786	42,1550	,40	98,10	
Pallidum_left_volume	Control Group	56	1,3804	,15305	,02045	1,3394	1,4213	1,10	1,90	
	Asymptomatic non-hospitalised COVID-19	51	1,4412	,15770	,02208	1,3968	1,4855	1,10	1,90	
	Severe hospitalised COVID-19	48	1,3417	,14562	,02102	1,2994	1,3840	1,00	1,60	
	Total	155	1,3884	,15665	,01258	1,3635	1,4132	1,00	1,90	
Pallidum_left_percentile	Control Group	56	27,0946	23,85160	3,18730	20,7071	33,4821	,10	86,10	
	Asymptomatic non-hospitalised COVID-19	51	28,5137	22,19231	3,10754	22,2720	34,7554	,60	81,60	
	Severe hospitalised COVID-19	48	25,0771	25,64006	3,70082	17,6320	32,5222	,10	80,80	
	Total	155	26,9368	23,78297	1,91029	23,1630	30,7105	,10	86,10	
Thalamus_right_volume	Control Group	56	8,1286	,75552	,10096	7,9262	8,3309	6,80	10,40	
	Asymptomatic non-hospitalised COVID-19	51	8,3471	,81126	,11360	8,1189	8,5752	6,40	10,00	
	Severe hospitalised COVID-19	48	7,6646	,77032	,11119	7,4409	7,8883	6,20	9,40	
	Total	155	8,0568	,82241	,06606	7,9263	8,1873	6,20	10,40	
Thalamus_right_percentile	Control Group	56	44,4339	30,42709	4,06599	36,2855	52,5824	,20	93,80	
	Asymptomatic non-hospitalised COVID-19	51	44,1392	28,16849	3,94438	36,2167	52,0617	,60	99,80	
	Severe hospitalised COVID-19	48	31,9335	27,93017	4,03137	23,8235	40,0436	,00	94,90	
	Total	155	40,4659	29,31263	2,35445	35,8147	45,1171	,00	99,80	
Thalamus_left_volume	Control Group	56	8,4571	,81058	,10832	8,2401	8,6742	7,30	10,80	
	Asymptomatic non-hospitalised COVID-19	51	8,4647	1,35216	,18934	8,0844	8,8450	1,40	10,50	
	Severe hospitalised COVID-19	48	7,9917	,77893	,11243	7,7655	8,2178	6,20	9,90	
	Total	155	8,3155	1,02995	,08273	8,1521	8,4789	1,40	10,80	
Thalamus_left_percentile	Control Group	56	56,7321	29,84449	3,98814	48,7397	64,7246	,70	97,90	
	Asymptomatic non-hospitalised COVID-19	51	54,3294	26,68210	3,73624	46,8249	61,8339	8,50	99,90	
	Severe hospitalised COVID-19	48	42,8271	29,38754	4,24173	34,2938	51,3603	,10	97,90	
	Total	155	51,6355	29,13519	2,34020	47,0125	56,2585	,10	99,90	
Brainstem_volume	Control Group	56	27,8714	10,30650	1,37726	25,1113	30,6315	21,00	99,00	
	Asymptomatic non-hospitalised COVID-19	51	26,3882	2,91819	,40863	25,5675	27,2090	21,00	32,20	
	Severe hospitalised COVID-19	48	24,8792	2,62386	,37872	24,1173	25,6411	20,00	30,30	
	Total	155	26,4568	6,65644	,53466	25,4006	27,5130	20,00	99,00	
Brainstem_percentile	Control Group	56	55,9107	24,96579	3,33619	49,2248	62,5966	8,00	99,90	
	Asymptomatic non-hospitalised COVID-19	51	56,3451	28,31822	3,96534	48,3805	64,3097	1,00	99,00	
	Severe hospitalised COVID-19	48	46,2521	26,65100	3,84674	38,5134	53,9907	,00	97,20	
	Total	155	53,0626	26,84614	2,15633	48,8028	57,3224	,00	99,90	
Mesencephalon_volume	Control Group	56	8,9107	12,28785	1,64203	5,6200	12,2014	6,00	99,00	
	Asymptomatic non-hospitalised COVID-19	51	7,2314	,89632	,12551	6,9793	7,4835	6,00	9,30	
	Severe hospitalised COVID-19	48	6,8104	,83825	,12099	6,5670	7,0538	5,00	8,60	
	Total	155	7,7077	7,43324	,59705	6,5283	8,8872	5,00	99,00	
Mesencephalon_percentile	Control Group	56	49,4179	27,36541	3,65686	42,0894	56,7464	4,00	99,00	
	Asymptomatic non-hospitalised COVID-19	51	49,8216	28,56335	3,99967	41,7880	57,8551	3,00	98,00	
	Severe hospitalised COVID-19	48	40,9000	27,23278	3,93071	32,9924	48,8076	,00	94,40	
	Total	155	46,9129	27,83946	2,23612	42,4955	51,3303	,00	99,00	
Pons_volume	Control Group	56	15,8554	11,17777	1,49369	12,8619	18,8488	11,00	97,00	
	Asymptomatic non-hospitalised COVID-19	51	14,3588	1,76637	,24734	13,8620	14,8556	11,00	18,00	
	Severe hospitalised COVID-19	48	13,5875	1,47007	,21219	13,1606	14,0144	11,00	17,00	
	Total	155	14,6606	6,87045	,55185	13,5705	15,7508	11,00	97,00	
Pons_percentile	Control Group	56	54,0107	27,00505	3,60870	46,7787	61,2427	3,00	122,00	
	Asymptomatic non-hospitalised COVID-19	51	51,1471	29,23668	4,09395	42,9241	59,3700	,10	98,00	
	Severe hospitalised COVID-19	48	44,3688	27,22555	3,92967	36,4633	52,2742	,00	98,00	
	Total	155	50,0826	27,93499	2,24379	45,6500	54,5152	,00	122,00	
Cerebellar_grey_matter_volume	Control Group	56	109,6161	11,10947	1,48457	106,6409	112,5912	88,00	138,00	
	Asymptomatic non-hospitalised COVID-19	51	112,5157	11,18308	1,56594	109,3704	115,6610	91,00	141,00	
	Severe hospitalised COVID-19	48	105,9792	11,47608	1,65643	102,6469	109,3115	84,00	135,00	
	Total	155	109,4439	11,47844	,92197	107,6225	111,2652	84,00	141,00	
Cerebellar_grey_matter_percentile	Control Group	56	68,0143	24,56272	3,28233	61,4363	74,5922	4,00	99,00	
	Asymptomatic non-hospitalised COVID-19	51	70,4529	25,59591	3,58414	63,2540	77,6519	8,00	100,00	
	Severe hospitalised COVID-19	48	60,2375	27,64961	3,99088	52,2089	68,2661	7,00	99,70	
	Total	155	66,4084	26,07165	2,09413	62,2715	70,5453	4,00	100,00	
Left_ventricle_volume	Control Group	56	10,0946	7,45960	,99683	8,0969	12,0923	2,00	43,40	
	Asymptomatic non-hospitalised COVID-19	51	8,5078	5,92246	,82931	6,8421	10,1736	2,00	30,70	
	Severe hospitalised COVID-19	48	8,8333	5,13782	,74158	7,3415	10,3252	2,00	26,00	
	Total	155	9,1819	6,30945	,50679	8,1808	10,1831	2,00	43,40	
Left_ventricle_percentile	Control Group	56	54,3732	31,06270	4,15093	46,0546	62,6919	1,00	100,00	
	Asymptomatic non-hospitalised COVID-19	51	48,4804	29,30734	4,10385	40,2376	56,7232	2,00	99,00	
	Severe hospitalised COVID-19	48	56,0688	27,70818	3,99933	48,0231	64,1144	3,00	99,00	
	Total	155	52,9594	29,46472	2,36666	48,2840	57,6347	1,00	100,00	
Right_ventricle_volume	Control Group	56	9,9071	6,20823	,82961	8,2446	11,5697	2,00	31,50	
	Asymptomatic non-hospitalised COVID-19	51	9,0118	5,55070	,77725	7,4506	10,5729	1,70	28,30	
	Severe hospitalised COVID-19	48	9,5562	5,43538	,78453	7,9780	11,1345	2,00	31,00	
	Total	155	9,5039	5,73804	,46089	8,5934	10,4144	1,70	31,50	
Right_ventricle_percentile	Control Group	56	54,6679	31,63840	4,22786	46,1950	63,1407	1,00	99,60	
	Asymptomatic non-hospitalised COVID-19	51	51,0784	29,46008	4,12524	42,7927	59,3642	1,90	99,00	
	Severe hospitalised COVID-19	48	56,6063	29,85360	4,30900	47,9377	65,2748	1,00	99,00	
	Total	155	54,0871	30,27176	2,43149	49,2837	58,8905	1,00	99,60	
Third_ventricle_volume	Control Group	56	,6875	,40679	,05436	,5786	,7964	,10	1,90	
	Asymptomatic non-hospitalised COVID-19	51	,7608	,39399	,05517	,6500	,8716	,00	1,50	
	Severe hospitalised COVID-19	48	,7813	,35047	,05059	,6795	,8830	,00	1,80	
	Total	155	,7406	,38557	,03097	,6795	,8018	,00	1,90	
Third_ventricle_percentile	Control Group	56	48,6429	29,00896	3,87649	40,8742	56,4115	4,00	99,80	
	Asymptomatic non-hospitalised COVID-19	51	43,4725	29,44218	4,12273	35,1918	51,7533	,80	98,00	
	Severe hospitalised COVID-19	48	59,1979	27,77680	4,00924	51,1324	67,2635	5,00	100,00	
	Total	155	50,2103	29,30125	2,35353	45,5609	54,8597	,80	100,00	
Fourth_ventricle_volume	Control Group	56	1,1607	,36813	,04919	1,0621	1,2593	,70	2,00	
	Asymptomatic non-hospitalised COVID-19	51	1,2039	,42472	,05947	1,0845	1,3234	,50	2,80	
	Severe hospitalised COVID-19	48	1,0958	,36318	,05242	,9904	1,2013	,00	2,00	
	Total	155	1,1548	,38616	,03102	1,0936	1,2161	,00	2,80	
Fourth_ventricle_percentile	Control Group	56	48,3393	28,67372	3,83169	40,6604	56,0182	3,80	100,00	
	Asymptomatic non-hospitalised COVID-19	51	54,3784	27,16137	3,80335	46,7392	62,0177	6,70	100,00	
	Severe hospitalised COVID-19	48	48,8208	30,70438	4,43180	39,9052	57,7365	5,00	100,00	
	Total	155	50,4755	28,78272	2,31188	45,9084	55,0426	3,80	100,00	


ANOVA	
	Sum of Squares	df	Mean Square	F	Sig.	
Whole_brain_volume	Between Groups	191776,067	2	95888,034	6,090	,003	
	Within Groups	2393260,312	152	15745,134			
	Total	2585036,379	154				
Whole_brain_precentile	Between Groups	3559,612	2	1779,806	3,637	,029	
	Within Groups	74374,244	152	489,304			
	Total	77933,856	154				
Whole_brain_white_substance	Between Groups	29610,891	2	14805,446	3,538	,032	
	Within Groups	636140,529	152	4185,135			
	Total	665751,420	154				
Whole_brain_white_substance_percentile	Between Groups	357,337	2	178,668	,480	,619	
	Within Groups	56526,579	152	371,885			
	Total	56883,916	154				
Whole_brain_grey_substance	Between Groups	82863,739	2	41431,870	8,103	,000	
	Within Groups	777214,586	152	5113,254			
	Total	860078,325	154				
Whole_brain_grey_substance_percentile	Between Groups	4301,794	2	2150,897	3,268	,041	
	Within Groups	100037,445	152	658,141			
	Total	104339,239	154				
Supratentorial_gross_cerebral_cortex_volume	Between Groups	34309,960	2	17154,980	6,591	,002	
	Within Groups	395598,199	152	2602,620			
	Total	429908,159	154				
Supratentorial_cerebral_cortex_percentile	Between Groups	3408,667	2	1704,333	2,476	,088	
	Within Groups	104647,304	152	688,469			
	Total	108055,971	154				
Frontal_right_volume	Between Groups	1547,254	2	773,627	7,542	,001	
	Within Groups	15591,287	152	102,574			
	Total	17138,540	154				
Frontal_right_percentile	Between Groups	6234,374	2	3117,187	4,135	,018	
	Within Groups	114572,700	152	753,768			
	Total	120807,075	154				
Frontal_left_volume	Between Groups	1129,077	2	564,539	4,787	,010	
	Within Groups	17924,640	152	117,925			
	Total	19053,717	154				
Frontal_left_percentile	Between Groups	4112,587	2	2056,293	2,853	,061	
	Within Groups	109538,004	152	720,645			
	Total	113650,591	154				
Parietal_right_volume	Between Groups	343,466	2	171,733	5,754	,004	
	Within Groups	4536,358	152	29,844			
	Total	4879,823	154				
Parietal_right_percentile	Between Groups	1742,300	2	871,150	1,745	,178	
	Within Groups	75882,341	152	499,226			
	Total	77624,642	154				
Parietal_left_volume	Between Groups	419,117	2	209,559	6,544	,002	
	Within Groups	4867,348	152	32,022			
	Total	5286,465	154				
Parietal_left_percentile	Between Groups	3474,460	2	1737,230	2,763	,066	
	Within Groups	95581,287	152	628,824			
	Total	99055,747	154				
Precuneus_right_volume	Between Groups	21,394	2	10,697	4,939	,008	
	Within Groups	329,194	152	2,166			
	Total	350,588	154				
Precuneus_right_percentile	Between Groups	683,275	2	341,638	,446	,641	
	Within Groups	116484,794	152	766,347			
	Total	117168,070	154				
Precuneus_left_volume	Between Groups	24,225	2	12,112	3,513	,032	
	Within Groups	524,141	152	3,448			
	Total	548,365	154				
Precuneus_left_percentile	Between Groups	2595,827	2	1297,913	1,785	,171	
	Within Groups	110546,585	152	727,280			
	Total	113142,412	154				
Occipital_right_volume	Between Groups	226,444	2	113,222	2,659	,073	
	Within Groups	6471,855	152	42,578			
	Total	6698,299	154				
Occipital_right_percentlile	Between Groups	385,121	2	192,561	,342	,711	
	Within Groups	85680,678	152	563,689			
	Total	86065,800	154				
Occipital_left_volume	Between Groups	340,164	2	170,082	3,538	,031	
	Within Groups	7307,644	152	48,077			
	Total	7647,808	154				
Occipital_left_percentile	Between Groups	846,543	2	423,272	,532	,589	
	Within Groups	120933,635	152	795,616			
	Total	121780,179	154				
Temporal_right_volume	Between Groups	710,010	2	355,005	5,569	,005	
	Within Groups	9688,729	152	63,742			
	Total	10398,739	154				
Temporal_right_percentile	Between Groups	978,143	2	489,071	,627	,536	
	Within Groups	118615,826	152	780,367			
	Total	119593,969	154				
Temporal_left_volume	Between Groups	441,312	2	220,656	3,171	,045	
	Within Groups	10576,243	152	69,581			
	Total	11017,555	154				
Temporal_left_percentlile	Between Groups	2728,319	2	1364,160	1,641	,197	
	Within Groups	126387,203	152	831,495			
	Total	129115,523	154				
Mesiotemporal_right_volume	Between Groups	197,907	2	98,953	,864	,423	
	Within Groups	17404,213	152	114,501			
	Total	17602,119	154				
Mesiotemporal_right_percentile	Between Groups	820,095	2	410,047	,416	,660	
	Within Groups	149719,973	152	985,000			
	Total	150540,067	154				
Mesiotemproal_left_volume	Between Groups	176,815	2	88,408	,917	,402	
	Within Groups	14653,056	152	96,402			
	Total	14829,871	154				
Mesiotemporal_left_percentile	Between Groups	691,229	2	345,614	,353	,703	
	Within Groups	148722,061	152	978,435			
	Total	149413,289	154				
Hippocampus_right_volume	Between Groups	21,378	2	10,689	,849	,430	
	Within Groups	1914,744	152	12,597			
	Total	1936,123	154				
Hippocampus_right_percentile	Between Groups	58,895	2	29,447	,039	,962	
	Within Groups	114206,250	152	751,357			
	Total	114265,145	154				
Hippocampus_left_volume	Between Groups	52,276	2	26,138	,566	,569	
	Within Groups	7022,846	152	46,203			
	Total	7075,122	154				
Hippocampus_left_percentile	Between Groups	3,889	2	1,944	,003	,997	
	Within Groups	104719,514	152	688,944			
	Total	104723,403	154				
Gyrus_parahippocampalis_right_volume	Between Groups	,882	2	,441	2,992	,053	
	Within Groups	22,416	152	,147			
	Total	23,299	154				
Gyrus_parahippocampalis_right_percentile	Between Groups	1400,156	2	700,078	,978	,378	
	Within Groups	108786,729	152	715,702			
	Total	110186,886	154				
Gyrus_parahippocampalis_left_volume	Between Groups	1,319	2	,659	5,563	,005	
	Within Groups	18,012	152	,119			
	Total	19,331	154				
Gyrus_parahippocampalis_left_percentile	Between Groups	3947,026	2	1973,513	2,897	,058	
	Within Groups	103552,090	152	681,264			
	Total	107499,116	154				
Regio_entorhinalis_right_volume	Between Groups	,409	2	,205	2,364	,097	
	Within Groups	13,162	152	,087			
	Total	13,572	154				
Regio_entorhinalis_right_percentile	Between Groups	657,366	2	328,683	,613	,543	
	Within Groups	81516,001	152	536,289			
	Total	82173,367	154				
Regio_entorhinalis_left_volume	Between Groups	,234	2	,117	1,322	,270	
	Within Groups	13,438	152	,088			
	Total	13,672	154				
Regio_entorhinalis_left_percentile	Between Groups	899,080	2	449,540	,634	,532	
	Within Groups	107816,592	152	709,320			
	Total	108715,672	154				
Nucleus_caudatus_right_volume	Between Groups	2,301	2	1,150	6,499	,002	
	Within Groups	26,905	152	,177			
	Total	29,206	154				
Nucleus_caudatus_right_percentile	Between Groups	4727,787	2	2363,893	3,497	,033	
	Within Groups	102734,051	152	675,882			
	Total	107461,838	154				
Nucleus_caudatus_left_volume	Between Groups	2,608	2	1,304	9,216	,000	
	Within Groups	21,507	152	,141			
	Total	24,115	154				
Nucleus_caudatus_left_percentile	Between Groups	4849,273	2	2424,636	4,494	,013	
	Within Groups	82008,129	152	539,527			
	Total	86857,402	154				
Putamen_right_volume	Between Groups	2,423	2	1,212	5,240	,006	
	Within Groups	35,146	152	,231			
	Total	37,569	154				
Putamen_right_percentile	Between Groups	1009,521	2	504,761	,886	,415	
	Within Groups	86621,245	152	569,877			
	Total	87630,767	154				
Putamen_left_volume	Between Groups	2,811	2	1,406	5,688	,004	
	Within Groups	37,564	152	,247			
	Total	40,376	154				
Putamen_left_percentile	Between Groups	1880,926	2	940,463	1,593	,207	
	Within Groups	89761,198	152	590,534			
	Total	91642,124	154				
Pallidum_right_volume	Between Groups	,216	2	,108	4,417	,014	
	Within Groups	3,711	152	,024			
	Total	3,926	154				
Pallidum_right_percentile	Between Groups	654,283	2	327,141	,434	,648	
	Within Groups	114457,414	152	753,009			
	Total	115111,696	154				
Pallidum_left_volume	Between Groups	,251	2	,125	5,396	,005	
	Within Groups	3,529	152	,023			
	Total	3,779	154				
Pallidum_left_percentile	Between Groups	294,227	2	147,113	,258	,773	
	Within Groups	86812,754	152	571,137			
	Total	87106,980	154				
Thalamus_right_volume	Between Groups	11,969	2	5,985	9,867	,000	
	Within Groups	92,191	152	,607			
	Total	104,160	154				
Thalamus_right_percentile	Between Groups	5064,344	2	2532,172	3,025	,052	
	Within Groups	127257,075	152	837,218			
	Total	132321,420	154				
Thalamus_left_volume	Between Groups	7,293	2	3,646	3,551	,031	
	Within Groups	156,070	152	1,027			
	Total	163,363	154				
Thalamus_left_percentile	Between Groups	5548,992	2	2774,496	3,369	,037	
	Within Groups	125175,383	152	823,522			
	Total	130724,375	154				
Brainstem_volume	Between Groups	231,774	2	115,887	2,672	,072	
	Within Groups	6591,686	152	43,366			
	Total	6823,460	154				
Brainstem_percentile	Between Groups	3230,163	2	1615,082	2,278	,106	
	Within Groups	107760,020	152	708,947			
	Total	110990,183	154				
Mesencephalon_volume	Between Groups	131,263	2	65,631	1,191	,307	
	Within Groups	8377,708	152	55,117			
	Total	8508,971	154				
Mesencephalon_percentile	Between Groups	2518,306	2	1259,153	1,638	,198	
	Within Groups	116837,208	152	768,666			
	Total	119355,514	154				
Pons_volume	Between Groups	139,856	2	69,928	1,491	,228	
	Within Groups	7129,414	152	46,904			
	Total	7269,270	154				
Pons_percentile	Between Groups	2488,979	2	1244,490	1,607	,204	
	Within Groups	117687,024	152	774,257			
	Total	120176,003	154				
Cerebellar_grey_matter_volume	Between Groups	1059,100	2	529,550	4,185	,017	
	Within Groups	19231,102	152	126,520			
	Total	20290,202	154				
Cerebellar_grey_matter_percentile	Between Groups	2806,531	2	1403,265	2,094	,127	
	Within Groups	101872,068	152	670,211			
	Total	104678,599	154				
Left_ventricle_volume	Between Groups	75,657	2	37,829	,950	,389	
	Within Groups	6054,952	152	39,835			
	Total	6130,609	154				
Left_ventricle_percentile	Between Groups	1599,141	2	799,570	,920	,401	
	Within Groups	132098,953	152	869,072			
	Total	133698,094	154				
Right_ventricle_volume	Between Groups	21,589	2	10,795	,325	,723	
	Within Groups	5048,868	152	33,216			
	Total	5070,458	154				
Right_ventricle_percentile	Between Groups	785,158	2	392,579	,425	,654	
	Within Groups	140337,317	152	923,272			
	Total	141122,474	154				
Third_ventricle_volume	Between Groups	,258	2	,129	,866	,423	
	Within Groups	22,636	152	,149			
	Total	22,894	154				
Third_ventricle_percentile	Between Groups	6330,155	2	3165,077	3,822	,024	
	Within Groups	125888,589	152	828,214			
	Total	132218,743	154				
Fourth_ventricle_volume	Between Groups	,292	2	,146	,979	,378	
	Within Groups	22,672	152	,149			
	Total	22,964	154				
Fourth_ventricle_percentile	Between Groups	1163,848	2	581,924	,700	,498	
	Within Groups	126416,699	152	831,689			
	Total	127580,547	154				


Robust Tests of Equality of Means	
	Statistica	df1	df2	Sig.	
Whole_brain_volume	Welch	6,072	2	99,536	,003	
Whole_brain_precentile	Welch	2,966	2	94,501	,056	
Whole_brain_white_substance	Welch	3,434	2	99,719	,036	
Whole_brain_white_substance_percentile	Welch	,354	2	95,510	,703	
Whole_brain_grey_substance	Welch	7,654	2	98,697	,001	
Whole_brain_grey_substance_percentile	Welch	3,254	2	99,513	,043	
Supratentorial_gross_cerebral_cortex_volume	Welch	6,674	2	98,854	,002	
Supratentorial_cerebral_cortex_percentile	Welch	2,437	2	100,168	,093	
Frontal_right_volume	Welch	7,896	2	99,998	,001	
Frontal_right_percentile	Welch	4,134	2	100,025	,019	
Frontal_left_volume	Welch	5,700	2	97,298	,005	
Frontal_left_percentile	Welch	2,829	2	99,546	,064	
Parietal_right_volume	Welch	5,719	2	99,531	,004	
Parietal_right_percentile	Welch	1,851	2	99,533	,162	
Parietal_left_volume	Welch	6,629	2	99,974	,002	
Parietal_left_percentile	Welch	2,719	2	100,123	,071	
Precuneus_right_volume	Welch	5,168	2	100,354	,007	
Precuneus_right_percentile	Welch	,432	2	100,995	,650	
Precuneus_left_volume	Welch	3,946	2	101,230	,022	
Precuneus_left_percentile	Welch	1,732	2	96,926	,182	
Occipital_right_volume	Welch	2,049	2	84,984	,135	
Occipital_right_percentlile	Welch	,341	2	100,623	,712	
Occipital_left_volume	Welch	5,054	2	98,004	,008	
Occipital_left_percentile	Welch	,539	2	99,947	,585	
Temporal_right_volume	Welch	5,294	2	98,871	,007	
Temporal_right_percentile	Welch	,620	2	100,384	,540	
Temporal_left_volume	Welch	3,467	2	99,831	,035	
Temporal_left_percentlile	Welch	1,694	2	100,778	,189	
Mesiotemporal_right_volume	Welch	,851	2	100,854	,430	
Mesiotemporal_right_percentile	Welch	,450	2	100,365	,639	
Mesiotemproal_left_volume	Welch	,917	2	100,976	,403	
Mesiotemporal_left_percentile	Welch	,390	2	100,490	,678	
Hippocampus_right_volume	Welch	2,320	2	92,920	,104	
Hippocampus_right_percentile	Welch	,037	2	100,226	,964	
Hippocampus_left_volume	Welch	1,438	2	71,812	,244	
Hippocampus_left_percentile	Welch	,003	2	100,653	,997	
Gyrus_parahippocampalis_right_volume	Welch	2,789	2	100,041	,066	
Gyrus_parahippocampalis_right_percentile	Welch	,874	2	100,787	,420	
Gyrus_parahippocampalis_left_volume	Welch	5,250	2	99,972	,007	
Gyrus_parahippocampalis_left_percentile	Welch	3,213	2	100,164	,044	
Regio_entorhinalis_right_volume	Welch	2,373	2	100,547	,098	
Regio_entorhinalis_right_percentile	Welch	,596	2	101,259	,553	
Regio_entorhinalis_left_volume	Welch	1,221	2	99,223	,299	
Regio_entorhinalis_left_percentile	Welch	,639	2	100,361	,530	
Nucleus_caudatus_right_volume	Welch	6,655	2	100,244	,002	
Nucleus_caudatus_right_percentile	Welch	3,619	2	99,957	,030	
Nucleus_caudatus_left_volume	Welch	9,488	2	99,775	,000	
Nucleus_caudatus_left_percentile	Welch	4,482	2	99,450	,014	
Putamen_right_volume	Welch	4,993	2	99,785	,009	
Putamen_right_percentile	Welch	,836	2	99,214	,436	
Putamen_left_volume	Welch	5,564	2	99,846	,005	
Putamen_left_percentile	Welch	1,609	2	100,649	,205	
Pallidum_right_volume	Welch	4,966	2	101,279	,009	
Pallidum_right_percentile	Welch	,461	2	99,840	,632	
Pallidum_left_volume	Welch	5,349	2	100,628	,006	
Pallidum_left_percentile	Welch	,251	2	99,698	,778	
Thalamus_right_volume	Welch	9,665	2	100,077	,000	
Thalamus_right_percentile	Welch	3,123	2	100,955	,048	
Thalamus_left_volume	Welch	5,050	2	96,554	,008	
Thalamus_left_percentile	Welch	3,210	2	100,390	,045	
Brainstem_volume	Welch	4,968	2	95,455	,009	
Brainstem_percentile	Welch	2,259	2	99,483	,110	
Mesencephalon_volume	Welch	3,556	2	93,740	,032	
Mesencephalon_percentile	Welch	1,657	2	100,338	,196	
Pons_volume	Welch	3,610	2	93,698	,031	
Pons_percentile	Welch	1,672	2	100,119	,193	
Cerebellar_grey_matter_volume	Welch	4,089	2	100,143	,020	
Cerebellar_grey_matter_percentile	Welch	1,926	2	99,300	,151	
Left_ventricle_volume	Welch	,798	2	101,061	,453	
Left_ventricle_percentile	Welch	,949	2	101,078	,390	
Right_ventricle_volume	Welch	,317	2	101,143	,729	
Right_ventricle_percentile	Welch	,443	2	100,801	,643	
Third_ventricle_volume	Welch	,852	2	101,112	,430	
Third_ventricle_percentile	Welch	3,895	2	100,641	,023	
Fourth_ventricle_volume	Welch	,962	2	99,894	,386	
Fourth_ventricle_percentile	Welch	,742	2	99,785	,479	

a. Asymptotically F distributed.	


Post Hoc Tests


Multiple Comparisons	
Bonferroni  	
Dependent Variable	(I) COVID_severity	(J) COVID_severity	Mean Difference (I-J)	Std. Error	Sig.	95% Confidence Interval	
						Lower Bound	Upper Bound	
Whole_brain_volume	Control Group	Asymptomatic non-hospitalised COVID-19	-34,01145	24,28768	,490	-92,8062	24,7833	
		Severe hospitalised COVID-19	53,47262	24,68171	,095	-6,2760	113,2212	
	Asymptomatic non-hospitalised COVID-19	Control Group	34,01145	24,28768	,490	-24,7833	92,8062	
		Severe hospitalised COVID-19	87,48407*	25,23394	,002	26,3986	148,5695	
	Severe hospitalised COVID-19	Control Group	-53,47262	24,68171	,095	-113,2212	6,2760	
		Asymptomatic non-hospitalised COVID-19	-87,48407*	25,23394	,002	-148,5695	-26,3986	
Whole_brain_precentile	Control Group	Asymptomatic non-hospitalised COVID-19	1,53207	4,28156	1,000	-8,8326	11,8967	
		Severe hospitalised COVID-19	11,00327*	4,35102	,037	,4705	21,5361	
	Asymptomatic non-hospitalised COVID-19	Control Group	-1,53207	4,28156	1,000	-11,8967	8,8326	
		Severe hospitalised COVID-19	9,47120	4,44837	,105	-1,2973	20,2397	
	Severe hospitalised COVID-19	Control Group	-11,00327*	4,35102	,037	-21,5361	-,4705	
		Asymptomatic non-hospitalised COVID-19	-9,47120	4,44837	,105	-20,2397	1,2973	
Whole_brain_white_substance	Control Group	Asymptomatic non-hospitalised COVID-19	-17,54538	12,52182	,490	-47,8578	12,7670	
		Severe hospitalised COVID-19	17,04702	12,72497	,547	-13,7571	47,8512	
	Asymptomatic non-hospitalised COVID-19	Control Group	17,54538	12,52182	,490	-12,7670	47,8578	
		Severe hospitalised COVID-19	34,59240*	13,00968	,026	3,0990	66,0858	
	Severe hospitalised COVID-19	Control Group	-17,04702	12,72497	,547	-47,8512	13,7571	
		Asymptomatic non-hospitalised COVID-19	-34,59240*	13,00968	,026	-66,0858	-3,0990	
Whole_brain_white_substance_percentile	Control Group	Asymptomatic non-hospitalised COVID-19	-,02171	3,73265	1,000	-9,0576	9,0142	
		Severe hospitalised COVID-19	3,27351	3,79321	1,000	-5,9090	12,4560	
	Asymptomatic non-hospitalised COVID-19	Control Group	,02171	3,73265	1,000	-9,0142	9,0576	
		Severe hospitalised COVID-19	3,29522	3,87808	1,000	-6,0927	12,6831	
	Severe hospitalised COVID-19	Control Group	-3,27351	3,79321	1,000	-12,4560	5,9090	
		Asymptomatic non-hospitalised COVID-19	-3,29522	3,87808	1,000	-12,6831	6,0927	
Whole_brain_grey_substance	Control Group	Asymptomatic non-hospitalised COVID-19	-17,04559	13,84081	,660	-50,5509	16,4598	
		Severe hospitalised COVID-19	39,48542*	14,06536	,017	5,4365	73,5343	
	Asymptomatic non-hospitalised COVID-19	Control Group	17,04559	13,84081	,660	-16,4598	50,5509	
		Severe hospitalised COVID-19	56,53100*	14,38006	,000	21,7203	91,3417	
	Severe hospitalised COVID-19	Control Group	-39,48542*	14,06536	,017	-73,5343	-5,4365	
		Asymptomatic non-hospitalised COVID-19	-56,53100*	14,38006	,000	-91,3417	-21,7203	
Whole_brain_grey_substance_percentile	Control Group	Asymptomatic non-hospitalised COVID-19	2,95515	4,96561	1,000	-9,0654	14,9757	
		Severe hospitalised COVID-19	12,48958*	5,04617	,043	,2740	24,7052	
	Asymptomatic non-hospitalised COVID-19	Control Group	-2,95515	4,96561	1,000	-14,9757	9,0654	
		Severe hospitalised COVID-19	9,53444	5,15907	,200	-2,9545	22,0233	
	Severe hospitalised COVID-19	Control Group	-12,48958*	5,04617	,043	-24,7052	-,2740	
		Asymptomatic non-hospitalised COVID-19	-9,53444	5,15907	,200	-22,0233	2,9545	
Supratentorial_gross_cerebral_cortex_volume	Control Group	Asymptomatic non-hospitalised COVID-19	-7,49237	9,87457	1,000	-31,3964	16,4116	
		Severe hospitalised COVID-19	27,89673*	10,03477	,018	3,6049	52,1885	
	Asymptomatic non-hospitalised COVID-19	Control Group	7,49237	9,87457	1,000	-16,4116	31,3964	
		Severe hospitalised COVID-19	35,38909*	10,25929	,002	10,5538	60,2244	
	Severe hospitalised COVID-19	Control Group	-27,89673*	10,03477	,018	-52,1885	-3,6049	
		Asymptomatic non-hospitalised COVID-19	-35,38909*	10,25929	,002	-60,2244	-10,5538	
Supratentorial_cerebral_cortex_percentile	Control Group	Asymptomatic non-hospitalised COVID-19	6,14580	5,07873	,684	-6,1486	18,4402	
		Severe hospitalised COVID-19	11,44077	5,16112	,084	-1,0531	23,9346	
	Asymptomatic non-hospitalised COVID-19	Control Group	-6,14580	5,07873	,684	-18,4402	6,1486	
		Severe hospitalised COVID-19	5,29498	5,27660	,952	-7,4784	18,0684	
	Severe hospitalised COVID-19	Control Group	-11,44077	5,16112	,084	-23,9346	1,0531	
		Asymptomatic non-hospitalised COVID-19	-5,29498	5,27660	,952	-18,0684	7,4784	
Frontal_right_volume	Control Group	Asymptomatic non-hospitalised COVID-19	-1,53694	1,96034	1,000	-6,2825	3,2086	
		Severe hospitalised COVID-19	5,96012*	1,99215	,010	1,1376	10,7826	
	Asymptomatic non-hospitalised COVID-19	Control Group	1,53694	1,96034	1,000	-3,2086	6,2825	
		Severe hospitalised COVID-19	7,49706*	2,03672	,001	2,5666	12,4275	
	Severe hospitalised COVID-19	Control Group	-5,96012*	1,99215	,010	-10,7826	-1,1376	
		Asymptomatic non-hospitalised COVID-19	-7,49706*	2,03672	,001	-12,4275	-2,5666	
Frontal_right_percentile	Control Group	Asymptomatic non-hospitalised COVID-19	8,46597	5,31412	,340	-4,3983	21,3302	
		Severe hospitalised COVID-19	15,45506*	5,40034	,014	2,3821	28,5280	
	Asymptomatic non-hospitalised COVID-19	Control Group	-8,46597	5,31412	,340	-21,3302	4,3983	
		Severe hospitalised COVID-19	6,98909	5,52116	,622	-6,3763	20,3545	
	Severe hospitalised COVID-19	Control Group	-15,45506*	5,40034	,014	-28,5280	-2,3821	
		Asymptomatic non-hospitalised COVID-19	-6,98909	5,52116	,622	-20,3545	6,3763	
Frontal_left_volume	Control Group	Asymptomatic non-hospitalised COVID-19	-,70389	2,10192	1,000	-5,7921	4,3844	
		Severe hospitalised COVID-19	5,46756*	2,13602	,034	,2968	10,6384	
	Asymptomatic non-hospitalised COVID-19	Control Group	,70389	2,10192	1,000	-4,3844	5,7921	
		Severe hospitalised COVID-19	6,17145*	2,18381	,016	,8849	11,4579	
	Severe hospitalised COVID-19	Control Group	-5,46756*	2,13602	,034	-10,6384	-,2968	
		Asymptomatic non-hospitalised COVID-19	-6,17145*	2,18381	,016	-11,4579	-,8849	
Frontal_left_percentile	Control Group	Asymptomatic non-hospitalised COVID-19	4,92675	5,19605	1,000	-7,6517	17,5052	
		Severe hospitalised COVID-19	12,57381	5,28035	,055	-,2087	25,3563	
	Asymptomatic non-hospitalised COVID-19	Control Group	-4,92675	5,19605	1,000	-17,5052	7,6517	
		Severe hospitalised COVID-19	7,64706	5,39849	,476	-5,4214	20,7155	
	Severe hospitalised COVID-19	Control Group	-12,57381	5,28035	,055	-25,3563	,2087	
		Asymptomatic non-hospitalised COVID-19	-7,64706	5,39849	,476	-20,7155	5,4214	
Parietal_right_volume	Control Group	Asymptomatic non-hospitalised COVID-19	-,43459	1,05741	1,000	-2,9943	2,1252	
		Severe hospitalised COVID-19	2,98869*	1,07457	,018	,3874	5,5900	
	Asymptomatic non-hospitalised COVID-19	Control Group	,43459	1,05741	1,000	-2,1252	2,9943	
		Severe hospitalised COVID-19	3,42328*	1,09861	,007	,7638	6,0828	
	Severe hospitalised COVID-19	Control Group	-2,98869*	1,07457	,018	-5,5900	-,3874	
		Asymptomatic non-hospitalised COVID-19	-3,42328*	1,09861	,007	-6,0828	-,7638	
Parietal_right_percentile	Control Group	Asymptomatic non-hospitalised COVID-19	7,89762	4,32475	,209	-2,5716	18,3668	
		Severe hospitalised COVID-19	5,29345	4,39492	,691	-5,3456	15,9325	
	Asymptomatic non-hospitalised COVID-19	Control Group	-7,89762	4,32475	,209	-18,3668	2,5716	
		Severe hospitalised COVID-19	-2,60417	4,49325	1,000	-13,4813	8,2729	
	Severe hospitalised COVID-19	Control Group	-5,29345	4,39492	,691	-15,9325	5,3456	
		Asymptomatic non-hospitalised COVID-19	2,60417	4,49325	1,000	-8,2729	13,4813	
Parietal_left_volume	Control Group	Asymptomatic non-hospitalised COVID-19	-,34387	1,09531	1,000	-2,9954	2,3076	
		Severe hospitalised COVID-19	3,37917*	1,11308	,008	,6847	6,0737	
	Asymptomatic non-hospitalised COVID-19	Control Group	,34387	1,09531	1,000	-2,3076	2,9954	
		Severe hospitalised COVID-19	3,72304*	1,13798	,004	,9682	6,4778	
	Severe hospitalised COVID-19	Control Group	-3,37917*	1,11308	,008	-6,0737	-,6847	
		Asymptomatic non-hospitalised COVID-19	-3,72304*	1,13798	,004	-6,4778	-,9682	
Parietal_left_percentile	Control Group	Asymptomatic non-hospitalised COVID-19	9,52199	4,85375	,155	-2,2278	21,2718	
		Severe hospitalised COVID-19	10,17946	4,93250	,122	-1,7609	22,1199	
	Asymptomatic non-hospitalised COVID-19	Control Group	-9,52199	4,85375	,155	-21,2718	2,2278	
		Severe hospitalised COVID-19	,65748	5,04286	1,000	-11,5501	12,8650	
	Severe hospitalised COVID-19	Control Group	-10,17946	4,93250	,122	-22,1199	1,7609	
		Asymptomatic non-hospitalised COVID-19	-,65748	5,04286	1,000	-12,8650	11,5501	
Precuneus_right_volume	Control Group	Asymptomatic non-hospitalised COVID-19	-,22703	,28485	1,000	-,9166	,4625	
		Severe hospitalised COVID-19	,66905	,28947	,066	-,0317	1,3698	
	Asymptomatic non-hospitalised COVID-19	Control Group	,22703	,28485	1,000	-,4625	,9166	
		Severe hospitalised COVID-19	,89608*	,29595	,009	,1797	1,6125	
	Severe hospitalised COVID-19	Control Group	-,66905	,28947	,066	-1,3698	,0317	
		Asymptomatic non-hospitalised COVID-19	-,89608*	,29595	,009	-1,6125	-,1797	
Precuneus_right_percentile	Control Group	Asymptomatic non-hospitalised COVID-19	2,10193	5,35828	1,000	-10,8692	15,0731	
		Severe hospitalised COVID-19	5,13244	5,44521	1,000	-8,0491	18,3140	
	Asymptomatic non-hospitalised COVID-19	Control Group	-2,10193	5,35828	1,000	-15,0731	10,8692	
		Severe hospitalised COVID-19	3,03051	5,56704	1,000	-10,4460	16,5070	
	Severe hospitalised COVID-19	Control Group	-5,13244	5,44521	1,000	-18,3140	8,0491	
		Asymptomatic non-hospitalised COVID-19	-3,03051	5,56704	1,000	-16,5070	10,4460	
Precuneus_left_volume	Control Group	Asymptomatic non-hospitalised COVID-19	-,25167	,35943	1,000	-1,1218	,6184	
		Severe hospitalised COVID-19	,70470	,36526	,167	-,1795	1,5889	
	Asymptomatic non-hospitalised COVID-19	Control Group	,25167	,35943	1,000	-,6184	1,1218	
		Severe hospitalised COVID-19	,95637*	,37343	,034	,0524	1,8604	
	Severe hospitalised COVID-19	Control Group	-,70470	,36526	,167	-1,5889	,1795	
		Asymptomatic non-hospitalised COVID-19	-,95637*	,37343	,034	-1,8604	-,0524	
Precuneus_left_percentile	Control Group	Asymptomatic non-hospitalised COVID-19	5,36310	5,21992	,918	-7,2731	17,9993	
		Severe hospitalised COVID-19	9,98393	5,30460	,185	-2,8573	22,8251	
	Asymptomatic non-hospitalised COVID-19	Control Group	-5,36310	5,21992	,918	-17,9993	7,2731	
		Severe hospitalised COVID-19	4,62083	5,42329	1,000	-8,5077	17,7493	
	Severe hospitalised COVID-19	Control Group	-9,98393	5,30460	,185	-22,8251	2,8573	
		Asymptomatic non-hospitalised COVID-19	-4,62083	5,42329	1,000	-17,7493	8,5077	
Occipital_right_volume	Control Group	Asymptomatic non-hospitalised COVID-19	-1,74737	1,26301	,506	-4,8048	1,3101	
		Severe hospitalised COVID-19	1,25863	1,28350	,985	-1,8484	4,3657	
	Asymptomatic non-hospitalised COVID-19	Control Group	1,74737	1,26301	,506	-1,3101	4,8048	
		Severe hospitalised COVID-19	3,00600	1,31221	,070	-,1706	6,1826	
	Severe hospitalised COVID-19	Control Group	-1,25863	1,28350	,985	-4,3657	1,8484	
		Asymptomatic non-hospitalised COVID-19	-3,00600	1,31221	,070	-6,1826	,1706	
Occipital_right_percentlile	Control Group	Asymptomatic non-hospitalised COVID-19	1,77826	4,59550	1,000	-9,3464	12,9029	
		Severe hospitalised COVID-19	3,86012	4,67005	1,000	-7,4450	15,1652	
	Asymptomatic non-hospitalised COVID-19	Control Group	-1,77826	4,59550	1,000	-12,9029	9,3464	
		Severe hospitalised COVID-19	2,08186	4,77454	1,000	-9,4762	13,6399	
	Severe hospitalised COVID-19	Control Group	-3,86012	4,67005	1,000	-15,1652	7,4450	
		Asymptomatic non-hospitalised COVID-19	-2,08186	4,77454	1,000	-13,6399	9,4762	
Occipital_left_volume	Control Group	Asymptomatic non-hospitalised COVID-19	,04195	1,34208	1,000	-3,2069	3,2908	
		Severe hospitalised COVID-19	3,22381	1,36386	,058	-,0778	6,5254	
	Asymptomatic non-hospitalised COVID-19	Control Group	-,04195	1,34208	1,000	-3,2908	3,2069	
		Severe hospitalised COVID-19	3,18186	1,39437	,072	-,1936	6,5573	
	Severe hospitalised COVID-19	Control Group	-3,22381	1,36386	,058	-6,5254	,0778	
		Asymptomatic non-hospitalised COVID-19	-3,18186	1,39437	,072	-6,5573	,1936	
Occipital_left_percentile	Control Group	Asymptomatic non-hospitalised COVID-19	2,45385	5,45965	1,000	-10,7627	15,6704	
		Severe hospitalised COVID-19	5,71905	5,54822	,913	-7,7119	19,1500	
	Asymptomatic non-hospitalised COVID-19	Control Group	-2,45385	5,45965	1,000	-15,6704	10,7627	
		Severe hospitalised COVID-19	3,26520	5,67236	1,000	-10,4663	16,9966	
	Severe hospitalised COVID-19	Control Group	-5,71905	5,54822	,913	-19,1500	7,7119	
		Asymptomatic non-hospitalised COVID-19	-3,26520	5,67236	1,000	-16,9966	10,4663	
Temporal_right_volume	Control Group	Asymptomatic non-hospitalised COVID-19	-2,23690	1,54534	,449	-5,9778	1,5040	
		Severe hospitalised COVID-19	3,10476	1,57041	,150	-,6968	6,9064	
	Asymptomatic non-hospitalised COVID-19	Control Group	2,23690	1,54534	,449	-1,5040	5,9778	
		Severe hospitalised COVID-19	5,34167*	1,60555	,003	1,4550	9,2283	
	Severe hospitalised COVID-19	Control Group	-3,10476	1,57041	,150	-6,9064	,6968	
		Asymptomatic non-hospitalised COVID-19	-5,34167*	1,60555	,003	-9,2283	-1,4550	
Temporal_right_percentile	Control Group	Asymptomatic non-hospitalised COVID-19	-1,53064	5,40707	1,000	-14,6199	11,5586	
		Severe hospitalised COVID-19	4,52708	5,49480	1,000	-8,7745	17,8287	
	Asymptomatic non-hospitalised COVID-19	Control Group	1,53064	5,40707	1,000	-11,5586	14,6199	
		Severe hospitalised COVID-19	6,05772	5,61774	,848	-7,5415	19,6569	
	Severe hospitalised COVID-19	Control Group	-4,52708	5,49480	1,000	-17,8287	8,7745	
		Asymptomatic non-hospitalised COVID-19	-6,05772	5,61774	,848	-19,6569	7,5415	
Temporal_left_volume	Control Group	Asymptomatic non-hospitalised COVID-19	-1,73375	1,61457	,854	-5,6422	2,1747	
		Severe hospitalised COVID-19	2,47470	1,64076	,401	-1,4972	6,4466	
	Asymptomatic non-hospitalised COVID-19	Control Group	1,73375	1,61457	,854	-2,1747	5,6422	
		Severe hospitalised COVID-19	4,20846*	1,67747	,039	,1477	8,2692	
	Severe hospitalised COVID-19	Control Group	-2,47470	1,64076	,401	-6,4466	1,4972	
		Asymptomatic non-hospitalised COVID-19	-4,20846*	1,67747	,039	-8,2692	-,1477	
Temporal_left_percentlile	Control Group	Asymptomatic non-hospitalised COVID-19	-2,61225	5,58139	1,000	-16,1235	10,8990	
		Severe hospitalised COVID-19	7,52083	5,67194	,561	-6,2096	21,2513	
	Asymptomatic non-hospitalised COVID-19	Control Group	2,61225	5,58139	1,000	-10,8990	16,1235	
		Severe hospitalised COVID-19	10,13309	5,79885	,248	-3,9046	24,1707	
	Severe hospitalised COVID-19	Control Group	-7,52083	5,67194	,561	-21,2513	6,2096	
		Asymptomatic non-hospitalised COVID-19	-10,13309	5,79885	,248	-24,1707	3,9046	
Mesiotemporal_right_volume	Control Group	Asymptomatic non-hospitalised COVID-19	-2,42717	2,07118	,729	-7,4410	2,5867	
		Severe hospitalised COVID-19	-,04911	2,10478	1,000	-5,1443	5,0461	
	Asymptomatic non-hospitalised COVID-19	Control Group	2,42717	2,07118	,729	-2,5867	7,4410	
		Severe hospitalised COVID-19	2,37806	2,15188	,813	-2,8311	7,5873	
	Severe hospitalised COVID-19	Control Group	,04911	2,10478	1,000	-5,0461	5,1443	
		Asymptomatic non-hospitalised COVID-19	-2,37806	2,15188	,813	-7,5873	2,8311	
Mesiotemporal_right_percentile	Control Group	Asymptomatic non-hospitalised COVID-19	-4,58708	6,07478	1,000	-19,2927	10,1185	
		Severe hospitalised COVID-19	,60655	6,17334	1,000	-14,3377	15,5508	
	Asymptomatic non-hospitalised COVID-19	Control Group	4,58708	6,07478	1,000	-10,1185	19,2927	
		Severe hospitalised COVID-19	5,19363	6,31146	1,000	-10,0849	20,4722	
	Severe hospitalised COVID-19	Control Group	-,60655	6,17334	1,000	-15,5508	14,3377	
		Asymptomatic non-hospitalised COVID-19	-5,19363	6,31146	1,000	-20,4722	10,0849	
Mesiotemproal_left_volume	Control Group	Asymptomatic non-hospitalised COVID-19	-2,25840	1,90044	,710	-6,8589	2,3421	
		Severe hospitalised COVID-19	,03155	1,93128	1,000	-4,6436	4,7067	
	Asymptomatic non-hospitalised COVID-19	Control Group	2,25840	1,90044	,710	-2,3421	6,8589	
		Severe hospitalised COVID-19	2,28995	1,97449	,744	-2,4898	7,0697	
	Severe hospitalised COVID-19	Control Group	-,03155	1,93128	1,000	-4,7067	4,6436	
		Asymptomatic non-hospitalised COVID-19	-2,28995	1,97449	,744	-7,0697	2,4898	
Mesiotemporal_left_percentile	Control Group	Asymptomatic non-hospitalised COVID-19	-4,40641	6,05451	1,000	-19,0629	10,2501	
		Severe hospitalised COVID-19	,18452	6,15273	1,000	-14,7098	15,0788	
	Asymptomatic non-hospitalised COVID-19	Control Group	4,40641	6,05451	1,000	-10,2501	19,0629	
		Severe hospitalised COVID-19	4,59093	6,29039	1,000	-10,6366	19,8185	
	Severe hospitalised COVID-19	Control Group	-,18452	6,15273	1,000	-15,0788	14,7098	
		Asymptomatic non-hospitalised COVID-19	-4,59093	6,29039	1,000	-19,8185	10,6366	
Hippocampus_right_volume	Control Group	Asymptomatic non-hospitalised COVID-19	,66849	,68698	,996	-,9945	2,3315	
		Severe hospitalised COVID-19	,85268	,69813	,672	-,8373	2,5427	
	Asymptomatic non-hospitalised COVID-19	Control Group	-,66849	,68698	,996	-2,3315	,9945	
		Severe hospitalised COVID-19	,18419	,71375	1,000	-1,5436	1,9120	
	Severe hospitalised COVID-19	Control Group	-,85268	,69813	,672	-2,5427	,8373	
		Asymptomatic non-hospitalised COVID-19	-,18419	,71375	1,000	-1,9120	1,5436	
Hippocampus_right_percentile	Control Group	Asymptomatic non-hospitalised COVID-19	-1,28620	5,30562	1,000	-14,1299	11,5574	
		Severe hospitalised COVID-19	,05387	5,39169	1,000	-12,9982	13,1059	
	Asymptomatic non-hospitalised COVID-19	Control Group	1,28620	5,30562	1,000	-11,5574	14,1299	
		Severe hospitalised COVID-19	1,34007	5,51233	1,000	-12,0040	14,6841	
	Severe hospitalised COVID-19	Control Group	-,05387	5,39169	1,000	-13,1059	12,9982	
		Asymptomatic non-hospitalised COVID-19	-1,34007	5,51233	1,000	-14,6841	12,0040	
Hippocampus_left_volume	Control Group	Asymptomatic non-hospitalised COVID-19	,67423	1,31567	1,000	-2,5107	3,8592	
		Severe hospitalised COVID-19	1,42202	1,33702	,868	-1,8146	4,6586	
	Asymptomatic non-hospitalised COVID-19	Control Group	-,67423	1,31567	1,000	-3,8592	2,5107	
		Severe hospitalised COVID-19	,74779	1,36693	1,000	-2,5612	4,0568	
	Severe hospitalised COVID-19	Control Group	-1,42202	1,33702	,868	-4,6586	1,8146	
		Asymptomatic non-hospitalised COVID-19	-,74779	1,36693	1,000	-4,0568	2,5612	
Hippocampus_left_percentile	Control Group	Asymptomatic non-hospitalised COVID-19	-,32395	5,08048	1,000	-12,6226	11,9747	
		Severe hospitalised COVID-19	,02679	5,16290	1,000	-12,4714	12,5250	
	Asymptomatic non-hospitalised COVID-19	Control Group	,32395	5,08048	1,000	-11,9747	12,6226	
		Severe hospitalised COVID-19	,35074	5,27842	1,000	-12,4271	13,1285	
	Severe hospitalised COVID-19	Control Group	-,02679	5,16290	1,000	-12,5250	12,4714	
		Asymptomatic non-hospitalised COVID-19	-,35074	5,27842	1,000	-13,1285	12,4271	
Gyrus_parahippocampalis_right_volume	Control Group	Asymptomatic non-hospitalised COVID-19	-,12679	,07433	,270	-,3067	,0532	
		Severe hospitalised COVID-19	,05655	,07554	1,000	-,1263	,2394	
	Asymptomatic non-hospitalised COVID-19	Control Group	,12679	,07433	,270	-,0532	,3067	
		Severe hospitalised COVID-19	,18333	,07723	,057	-,0036	,3703	
	Severe hospitalised COVID-19	Control Group	-,05655	,07554	1,000	-,2394	,1263	
		Asymptomatic non-hospitalised COVID-19	-,18333	,07723	,057	-,3703	,0036	
Gyrus_parahippocampalis_right_percentile	Control Group	Asymptomatic non-hospitalised COVID-19	-7,05021	5,17820	,526	-19,5854	5,4850	
		Severe hospitalised COVID-19	-4,84911	5,26221	1,000	-17,5877	7,8895	
	Asymptomatic non-hospitalised COVID-19	Control Group	7,05021	5,17820	,526	-5,4850	19,5854	
		Severe hospitalised COVID-19	2,20110	5,37995	1,000	-10,8225	15,2247	
	Severe hospitalised COVID-19	Control Group	4,84911	5,26221	1,000	-7,8895	17,5877	
		Asymptomatic non-hospitalised COVID-19	-2,20110	5,37995	1,000	-15,2247	10,8225	
Gyrus_parahippocampalis_left_volume	Control Group	Asymptomatic non-hospitalised COVID-19	-,14009	,06663	,111	-,3014	,0212	
		Severe hospitalised COVID-19	,08810	,06771	,586	-,0758	,2520	
	Asymptomatic non-hospitalised COVID-19	Control Group	,14009	,06663	,111	-,0212	,3014	
		Severe hospitalised COVID-19	,22819*	,06923	,004	,0606	,3958	
	Severe hospitalised COVID-19	Control Group	-,08810	,06771	,586	-,2520	,0758	
		Asymptomatic non-hospitalised COVID-19	-,22819*	,06923	,004	-,3958	-,0606	
Gyrus_parahippocampalis_left_percentile	Control Group	Asymptomatic non-hospitalised COVID-19	-10,85266	5,05208	,100	-23,0826	1,3772	
		Severe hospitalised COVID-19	-,24911	5,13405	1,000	-12,6774	12,1792	
	Asymptomatic non-hospitalised COVID-19	Control Group	10,85266	5,05208	,100	-1,3772	23,0826	
		Severe hospitalised COVID-19	10,60355	5,24891	,135	-2,1028	23,3099	
	Severe hospitalised COVID-19	Control Group	,24911	5,13405	1,000	-12,1792	12,6774	
		Asymptomatic non-hospitalised COVID-19	-10,60355	5,24891	,135	-23,3099	2,1028	
Regio_entorhinalis_right_volume	Control Group	Asymptomatic non-hospitalised COVID-19	-,09072	,05696	,340	-,2286	,0472	
		Severe hospitalised COVID-19	,03244	,05788	1,000	-,1077	,1726	
	Asymptomatic non-hospitalised COVID-19	Control Group	,09072	,05696	,340	-,0472	,2286	
		Severe hospitalised COVID-19	,12316	,05918	,117	-,0201	,2664	
	Severe hospitalised COVID-19	Control Group	-,03244	,05788	1,000	-,1726	,1077	
		Asymptomatic non-hospitalised COVID-19	-,12316	,05918	,117	-,2664	,0201	
Regio_entorhinalis_right_percentile	Control Group	Asymptomatic non-hospitalised COVID-19	-4,90630	4,48242	,826	-15,7572	5,9446	
		Severe hospitalised COVID-19	-1,66905	4,55514	1,000	-12,6960	9,3579	
	Asymptomatic non-hospitalised COVID-19	Control Group	4,90630	4,48242	,826	-5,9446	15,7572	
		Severe hospitalised COVID-19	3,23725	4,65706	1,000	-8,0364	14,5109	
	Severe hospitalised COVID-19	Control Group	1,66905	4,55514	1,000	-9,3579	12,6960	
		Asymptomatic non-hospitalised COVID-19	-3,23725	4,65706	1,000	-14,5109	8,0364	
Regio_entorhinalis_left_volume	Control Group	Asymptomatic non-hospitalised COVID-19	-,06408	,05755	,802	-,2034	,0752	
		Severe hospitalised COVID-19	,03065	,05849	1,000	-,1109	,1722	
	Asymptomatic non-hospitalised COVID-19	Control Group	,06408	,05755	,802	-,0752	,2034	
		Severe hospitalised COVID-19	,09473	,05979	,346	-,0500	,2395	
	Severe hospitalised COVID-19	Control Group	-,03065	,05849	1,000	-,1722	,1109	
		Asymptomatic non-hospitalised COVID-19	-,09473	,05979	,346	-,2395	,0500	
Regio_entorhinalis_left_percentile	Control Group	Asymptomatic non-hospitalised COVID-19	-4,87787	5,15506	1,000	-17,3571	7,6013	
		Severe hospitalised COVID-19	,49762	5,23869	1,000	-12,1840	13,1793	
	Asymptomatic non-hospitalised COVID-19	Control Group	4,87787	5,15506	1,000	-7,6013	17,3571	
		Severe hospitalised COVID-19	5,37549	5,35591	,951	-7,5899	18,3409	
	Severe hospitalised COVID-19	Control Group	-,49762	5,23869	1,000	-13,1793	12,1840	
		Asymptomatic non-hospitalised COVID-19	-5,37549	5,35591	,951	-18,3409	7,5899	
Nucleus_caudatus_right_volume	Control Group	Asymptomatic non-hospitalised COVID-19	-,18824	,08143	,066	-,3854	,0089	
		Severe hospitalised COVID-19	,11250	,08276	,528	-,0878	,3128	
	Asymptomatic non-hospitalised COVID-19	Control Group	,18824	,08143	,066	-,0089	,3854	
		Severe hospitalised COVID-19	,30074*	,08461	,002	,0959	,5056	
	Severe hospitalised COVID-19	Control Group	-,11250	,08276	,528	-,3128	,0878	
		Asymptomatic non-hospitalised COVID-19	-,30074*	,08461	,002	-,5056	-,0959	
Nucleus_caudatus_right_percentile	Control Group	Asymptomatic non-hospitalised COVID-19	-11,69377	5,03209	,064	-23,8753	,4877	
		Severe hospitalised COVID-19	,12976	5,11373	1,000	-12,2494	12,5089	
	Asymptomatic non-hospitalised COVID-19	Control Group	11,69377	5,03209	,064	-,4877	23,8753	
		Severe hospitalised COVID-19	11,82353	5,22814	,075	-,8326	24,4796	
	Severe hospitalised COVID-19	Control Group	-,12976	5,11373	1,000	-12,5089	12,2494	
		Asymptomatic non-hospitalised COVID-19	-11,82353	5,22814	,075	-24,4796	,8326	
Nucleus_caudatus_left_volume	Control Group	Asymptomatic non-hospitalised COVID-19	-,22223*	,07281	,008	-,3985	-,0460	
		Severe hospitalised COVID-19	,09137	,07399	,656	-,0877	,2705	
	Asymptomatic non-hospitalised COVID-19	Control Group	,22223*	,07281	,008	,0460	,3985	
		Severe hospitalised COVID-19	,31360*	,07565	,000	,1305	,4967	
	Severe hospitalised COVID-19	Control Group	-,09137	,07399	,656	-,2705	,0877	
		Asymptomatic non-hospitalised COVID-19	-,31360*	,07565	,000	-,4967	-,1305	
Nucleus_caudatus_left_percentile	Control Group	Asymptomatic non-hospitalised COVID-19	-12,13999*	4,49593	,023	-23,0236	-1,2564	
		Severe hospitalised COVID-19	-,53006	4,56887	1,000	-11,5902	10,5301	
	Asymptomatic non-hospitalised COVID-19	Control Group	12,13999*	4,49593	,023	1,2564	23,0236	
		Severe hospitalised COVID-19	11,60993*	4,67109	,042	,3023	22,9175	
	Severe hospitalised COVID-19	Control Group	,53006	4,56887	1,000	-10,5301	11,5902	
		Asymptomatic non-hospitalised COVID-19	-11,60993*	4,67109	,042	-22,9175	-,3023	
Putamen_right_volume	Control Group	Asymptomatic non-hospitalised COVID-19	-,14013	,09307	,403	-,3654	,0852	
		Severe hospitalised COVID-19	,17262	,09458	,210	-,0563	,4016	
	Asymptomatic non-hospitalised COVID-19	Control Group	,14013	,09307	,403	-,0852	,3654	
		Severe hospitalised COVID-19	,31275*	,09670	,004	,0787	,5468	
	Severe hospitalised COVID-19	Control Group	-,17262	,09458	,210	-,4016	,0563	
		Asymptomatic non-hospitalised COVID-19	-,31275*	,09670	,004	-,5468	-,0787	
Putamen_right_percentile	Control Group	Asymptomatic non-hospitalised COVID-19	-4,26348	4,62065	1,000	-15,4490	6,9220	
		Severe hospitalised COVID-19	1,94583	4,69561	1,000	-9,4211	13,3128	
	Asymptomatic non-hospitalised COVID-19	Control Group	4,26348	4,62065	1,000	-6,9220	15,4490	
		Severe hospitalised COVID-19	6,20931	4,80068	,593	-5,4120	17,8306	
	Severe hospitalised COVID-19	Control Group	-1,94583	4,69561	1,000	-13,3128	9,4211	
		Asymptomatic non-hospitalised COVID-19	-6,20931	4,80068	,593	-17,8306	5,4120	
Putamen_left_volume	Control Group	Asymptomatic non-hospitalised COVID-19	-,14384	,09622	,411	-,3768	,0891	
		Severe hospitalised COVID-19	,19256	,09778	,152	-,0442	,4293	
	Asymptomatic non-hospitalised COVID-19	Control Group	,14384	,09622	,411	-,0891	,3768	
		Severe hospitalised COVID-19	,33640*	,09997	,003	,0944	,5784	
	Severe hospitalised COVID-19	Control Group	-,19256	,09778	,152	-,4293	,0442	
		Asymptomatic non-hospitalised COVID-19	-,33640*	,09997	,003	-,5784	-,0944	
Putamen_left_percentile	Control Group	Asymptomatic non-hospitalised COVID-19	-4,12279	4,70365	1,000	-15,5092	7,2636	
		Severe hospitalised COVID-19	4,59792	4,77996	1,000	-6,9733	16,1691	
	Asymptomatic non-hospitalised COVID-19	Control Group	4,12279	4,70365	1,000	-7,2636	15,5092	
		Severe hospitalised COVID-19	8,72071	4,88691	,229	-3,1094	20,5508	
	Severe hospitalised COVID-19	Control Group	-4,59792	4,77996	1,000	-16,1691	6,9733	
		Asymptomatic non-hospitalised COVID-19	-8,72071	4,88691	,229	-20,5508	3,1094	
Pallidum_right_volume	Control Group	Asymptomatic non-hospitalised COVID-19	-,04576	,03024	,397	-,1190	,0274	
		Severe hospitalised COVID-19	,04762	,03073	,370	-,0268	,1220	
	Asymptomatic non-hospitalised COVID-19	Control Group	,04576	,03024	,397	-,0274	,1190	
		Severe hospitalised COVID-19	,09338*	,03142	,010	,0173	,1694	
	Severe hospitalised COVID-19	Control Group	-,04762	,03073	,370	-,1220	,0268	
		Asymptomatic non-hospitalised COVID-19	-,09338*	,03142	,010	-,1694	-,0173	
Pallidum_right_percentile	Control Group	Asymptomatic non-hospitalised COVID-19	-4,07780	5,31145	1,000	-16,9356	8,7800	
		Severe hospitalised COVID-19	,57649	5,39762	1,000	-12,4899	13,6429	
	Asymptomatic non-hospitalised COVID-19	Control Group	4,07780	5,31145	1,000	-8,7800	16,9356	
		Severe hospitalised COVID-19	4,65429	5,51839	1,000	-8,7044	18,0130	
	Severe hospitalised COVID-19	Control Group	-,57649	5,39762	1,000	-13,6429	12,4899	
		Asymptomatic non-hospitalised COVID-19	-4,65429	5,51839	1,000	-18,0130	8,7044	
Pallidum_left_volume	Control Group	Asymptomatic non-hospitalised COVID-19	-,06082	,02949	,123	-,1322	,0106	
		Severe hospitalised COVID-19	,03869	,02997	,596	-,0339	,1112	
	Asymptomatic non-hospitalised COVID-19	Control Group	,06082	,02949	,123	-,0106	,1322	
		Severe hospitalised COVID-19	,09951*	,03064	,004	,0253	,1737	
	Severe hospitalised COVID-19	Control Group	-,03869	,02997	,596	-,1112	,0339	
		Asymptomatic non-hospitalised COVID-19	-,09951*	,03064	,004	-,1737	-,0253	
Pallidum_left_percentile	Control Group	Asymptomatic non-hospitalised COVID-19	-1,41908	4,62576	1,000	-12,6170	9,7788	
		Severe hospitalised COVID-19	2,01756	4,70080	1,000	-9,3620	13,3971	
	Asymptomatic non-hospitalised COVID-19	Control Group	1,41908	4,62576	1,000	-9,7788	12,6170	
		Severe hospitalised COVID-19	3,43664	4,80598	1,000	-8,1975	15,0708	
	Severe hospitalised COVID-19	Control Group	-2,01756	4,70080	1,000	-13,3971	9,3620	
		Asymptomatic non-hospitalised COVID-19	-3,43664	4,80598	1,000	-15,0708	8,1975	
Thalamus_right_volume	Control Group	Asymptomatic non-hospitalised COVID-19	-,21849	,15074	,448	-,5834	,1464	
		Severe hospitalised COVID-19	,46399*	,15319	,009	,0932	,8348	
	Asymptomatic non-hospitalised COVID-19	Control Group	,21849	,15074	,448	-,1464	,5834	
		Severe hospitalised COVID-19	,68248*	,15662	,000	,3033	1,0616	
	Severe hospitalised COVID-19	Control Group	-,46399*	,15319	,009	-,8348	-,0932	
		Asymptomatic non-hospitalised COVID-19	-,68248*	,15662	,000	-1,0616	-,3033	
Thalamus_right_percentile	Control Group	Asymptomatic non-hospitalised COVID-19	,29471	5,60057	1,000	-13,2629	13,8524	
		Severe hospitalised COVID-19	12,50039	5,69143	,089	-1,2772	26,2780	
	Asymptomatic non-hospitalised COVID-19	Control Group	-,29471	5,60057	1,000	-13,8524	13,2629	
		Severe hospitalised COVID-19	12,20567	5,81877	,113	-1,8802	26,2915	
	Severe hospitalised COVID-19	Control Group	-12,50039	5,69143	,089	-26,2780	1,2772	
		Asymptomatic non-hospitalised COVID-19	-12,20567	5,81877	,113	-26,2915	1,8802	
Thalamus_left_volume	Control Group	Asymptomatic non-hospitalised COVID-19	-,00756	,19613	1,000	-,4824	,4672	
		Severe hospitalised COVID-19	,46548	,19932	,062	-,0170	,9480	
	Asymptomatic non-hospitalised COVID-19	Control Group	,00756	,19613	1,000	-,4672	,4824	
		Severe hospitalised COVID-19	,47304	,20377	,065	-,0203	,9663	
	Severe hospitalised COVID-19	Control Group	-,46548	,19932	,062	-,9480	,0170	
		Asymptomatic non-hospitalised COVID-19	-,47304	,20377	,065	-,9663	,0203	
Thalamus_left_percentile	Control Group	Asymptomatic non-hospitalised COVID-19	2,40273	5,55457	1,000	-11,0436	15,8490	
		Severe hospitalised COVID-19	13,90506*	5,64468	,045	,2406	27,5695	
	Asymptomatic non-hospitalised COVID-19	Control Group	-2,40273	5,55457	1,000	-15,8490	11,0436	
		Severe hospitalised COVID-19	11,50233	5,77098	,144	-2,4679	25,4725	
	Severe hospitalised COVID-19	Control Group	-13,90506*	5,64468	,045	-27,5695	-,2406	
		Asymptomatic non-hospitalised COVID-19	-11,50233	5,77098	,144	-25,4725	2,4679	
Brainstem_volume	Control Group	Asymptomatic non-hospitalised COVID-19	1,48319	1,27465	,739	-1,6024	4,5688	
		Severe hospitalised COVID-19	2,99226	1,29532	,067	-,1434	6,1279	
	Asymptomatic non-hospitalised COVID-19	Control Group	-1,48319	1,27465	,739	-4,5688	1,6024	
		Severe hospitalised COVID-19	1,50907	1,32431	,769	-1,6968	4,7149	
	Severe hospitalised COVID-19	Control Group	-2,99226	1,29532	,067	-6,1279	,1434	
		Asymptomatic non-hospitalised COVID-19	-1,50907	1,32431	,769	-4,7149	1,6968	
Brainstem_percentile	Control Group	Asymptomatic non-hospitalised COVID-19	-,43438	5,15371	1,000	-12,9103	12,0415	
		Severe hospitalised COVID-19	9,65863	5,23732	,201	-3,0197	22,3370	
	Asymptomatic non-hospitalised COVID-19	Control Group	,43438	5,15371	1,000	-12,0415	12,9103	
		Severe hospitalised COVID-19	10,09301	5,35450	,184	-2,8690	23,0550	
	Severe hospitalised COVID-19	Control Group	-9,65863	5,23732	,201	-22,3370	3,0197	
		Asymptomatic non-hospitalised COVID-19	-10,09301	5,35450	,184	-23,0550	2,8690	
Mesencephalon_volume	Control Group	Asymptomatic non-hospitalised COVID-19	1,67934	1,43699	,733	-1,7993	5,1580	
		Severe hospitalised COVID-19	2,10030	1,46030	,457	-1,4348	5,6353	
	Asymptomatic non-hospitalised COVID-19	Control Group	-1,67934	1,43699	,733	-5,1580	1,7993	
		Severe hospitalised COVID-19	,42096	1,49298	1,000	-3,1932	4,0351	
	Severe hospitalised COVID-19	Control Group	-2,10030	1,46030	,457	-5,6353	1,4348	
		Asymptomatic non-hospitalised COVID-19	-,42096	1,49298	1,000	-4,0351	3,1932	
Mesencephalon_percentile	Control Group	Asymptomatic non-hospitalised COVID-19	-,40371	5,36638	1,000	-13,3945	12,5870	
		Severe hospitalised COVID-19	8,51786	5,45344	,361	-4,6836	21,7194	
	Asymptomatic non-hospitalised COVID-19	Control Group	,40371	5,36638	1,000	-12,5870	13,3945	
		Severe hospitalised COVID-19	8,92157	5,57546	,335	-4,5753	22,4184	
	Severe hospitalised COVID-19	Control Group	-8,51786	5,45344	,361	-21,7194	4,6836	
		Asymptomatic non-hospitalised COVID-19	-8,92157	5,57546	,335	-22,4184	4,5753	
Pons_volume	Control Group	Asymptomatic non-hospitalised COVID-19	1,49653	1,32562	,782	-1,7125	4,7055	
		Severe hospitalised COVID-19	2,26786	1,34712	,283	-,9932	5,5289	
	Asymptomatic non-hospitalised COVID-19	Control Group	-1,49653	1,32562	,782	-4,7055	1,7125	
		Severe hospitalised COVID-19	,77132	1,37726	1,000	-2,5627	4,1054	
	Severe hospitalised COVID-19	Control Group	-2,26786	1,34712	,283	-5,5289	,9932	
		Asymptomatic non-hospitalised COVID-19	-,77132	1,37726	1,000	-4,1054	2,5627	
Pons_percentile	Control Group	Asymptomatic non-hospitalised COVID-19	2,86366	5,38586	1,000	-10,1743	15,9016	
		Severe hospitalised COVID-19	9,64196	5,47324	,240	-3,6075	22,8914	
	Asymptomatic non-hospitalised COVID-19	Control Group	-2,86366	5,38586	1,000	-15,9016	10,1743	
		Severe hospitalised COVID-19	6,77831	5,59570	,683	-6,7676	20,3242	
	Severe hospitalised COVID-19	Control Group	-9,64196	5,47324	,240	-22,8914	3,6075	
		Asymptomatic non-hospitalised COVID-19	-6,77831	5,59570	,683	-20,3242	6,7676	
Cerebellar_grey_matter_volume	Control Group	Asymptomatic non-hospitalised COVID-19	-2,89961	2,17717	,555	-8,1700	2,3708	
		Severe hospitalised COVID-19	3,63690	2,21250	,307	-1,7190	8,9928	
	Asymptomatic non-hospitalised COVID-19	Control Group	2,89961	2,17717	,555	-2,3708	8,1700	
		Severe hospitalised COVID-19	6,53652*	2,26200	,013	1,0608	12,0123	
	Severe hospitalised COVID-19	Control Group	-3,63690	2,21250	,307	-8,9928	1,7190	
		Asymptomatic non-hospitalised COVID-19	-6,53652*	2,26200	,013	-12,0123	-1,0608	
Cerebellar_grey_matter_percentile	Control Group	Asymptomatic non-hospitalised COVID-19	-2,43866	5,01093	1,000	-14,5689	9,6916	
		Severe hospitalised COVID-19	7,77679	5,09223	,386	-4,5503	20,1039	
	Asymptomatic non-hospitalised COVID-19	Control Group	2,43866	5,01093	1,000	-9,6916	14,5689	
		Severe hospitalised COVID-19	10,21544	5,20616	,155	-2,3875	22,8183	
	Severe hospitalised COVID-19	Control Group	-7,77679	5,09223	,386	-20,1039	4,5503	
		Asymptomatic non-hospitalised COVID-19	-10,21544	5,20616	,155	-22,8183	2,3875	
Left_ventricle_volume	Control Group	Asymptomatic non-hospitalised COVID-19	1,58680	1,22165	,588	-1,3705	4,5441	
		Severe hospitalised COVID-19	1,26131	1,24147	,934	-1,7440	4,2666	
	Asymptomatic non-hospitalised COVID-19	Control Group	-1,58680	1,22165	,588	-4,5441	1,3705	
		Severe hospitalised COVID-19	-,32549	1,26924	1,000	-3,3980	2,7471	
	Severe hospitalised COVID-19	Control Group	-1,26131	1,24147	,934	-4,2666	1,7440	
		Asymptomatic non-hospitalised COVID-19	,32549	1,26924	1,000	-2,7471	3,3980	
Left_ventricle_percentile	Control Group	Asymptomatic non-hospitalised COVID-19	5,89282	5,70612	,910	-7,9203	19,7060	
		Severe hospitalised COVID-19	-1,69554	5,79869	1,000	-15,7328	12,3417	
	Asymptomatic non-hospitalised COVID-19	Control Group	-5,89282	5,70612	,910	-19,7060	7,9203	
		Severe hospitalised COVID-19	-7,58836	5,92843	,607	-21,9397	6,7630	
	Severe hospitalised COVID-19	Control Group	1,69554	5,79869	1,000	-12,3417	15,7328	
		Asymptomatic non-hospitalised COVID-19	7,58836	5,92843	,607	-6,7630	21,9397	
Right_ventricle_volume	Control Group	Asymptomatic non-hospitalised COVID-19	,89538	1,11555	1,000	-1,8051	3,5959	
		Severe hospitalised COVID-19	,35089	1,13365	1,000	-2,3934	3,0952	
	Asymptomatic non-hospitalised COVID-19	Control Group	-,89538	1,11555	1,000	-3,5959	1,8051	
		Severe hospitalised COVID-19	-,54449	1,15901	1,000	-3,3502	2,2612	
	Severe hospitalised COVID-19	Control Group	-,35089	1,13365	1,000	-3,0952	2,3934	
		Asymptomatic non-hospitalised COVID-19	,54449	1,15901	1,000	-2,2612	3,3502	
Right_ventricle_percentile	Control Group	Asymptomatic non-hospitalised COVID-19	3,58943	5,88136	1,000	-10,6480	17,8268	
		Severe hospitalised COVID-19	-1,93839	5,97677	1,000	-16,4068	12,5300	
	Asymptomatic non-hospitalised COVID-19	Control Group	-3,58943	5,88136	1,000	-17,8268	10,6480	
		Severe hospitalised COVID-19	-5,52782	6,11050	1,000	-20,3199	9,2643	
	Severe hospitalised COVID-19	Control Group	1,93839	5,97677	1,000	-12,5300	16,4068	
		Asymptomatic non-hospitalised COVID-19	5,52782	6,11050	1,000	-9,2643	20,3199	
Third_ventricle_volume	Control Group	Asymptomatic non-hospitalised COVID-19	-,07328	,07469	,984	-,2541	,1075	
		Severe hospitalised COVID-19	-,09375	,07591	,656	-,2775	,0900	
	Asymptomatic non-hospitalised COVID-19	Control Group	,07328	,07469	,984	-,1075	,2541	
		Severe hospitalised COVID-19	-,02047	,07760	1,000	-,2083	,1674	
	Severe hospitalised COVID-19	Control Group	,09375	,07591	,656	-,0900	,2775	
		Asymptomatic non-hospitalised COVID-19	,02047	,07760	1,000	-,1674	,2083	
Third_ventricle_percentile	Control Group	Asymptomatic non-hospitalised COVID-19	5,17031	5,57037	1,000	-8,3143	18,6549	
		Severe hospitalised COVID-19	-10,55506	5,66074	,192	-24,2584	3,1483	
	Asymptomatic non-hospitalised COVID-19	Control Group	-5,17031	5,57037	1,000	-18,6549	8,3143	
		Severe hospitalised COVID-19	-15,72537*	5,78740	,022	-29,7353	-1,7154	
	Severe hospitalised COVID-19	Control Group	10,55506	5,66074	,192	-3,1483	24,2584	
		Asymptomatic non-hospitalised COVID-19	15,72537*	5,78740	,022	1,7154	29,7353	
Fourth_ventricle_volume	Control Group	Asymptomatic non-hospitalised COVID-19	-,04321	,07475	1,000	-,2242	,1378	
		Severe hospitalised COVID-19	,06488	,07597	1,000	-,1190	,2488	
	Asymptomatic non-hospitalised COVID-19	Control Group	,04321	,07475	1,000	-,1378	,2242	
		Severe hospitalised COVID-19	,10809	,07767	,498	-,0799	,2961	
	Severe hospitalised COVID-19	Control Group	-,06488	,07597	1,000	-,2488	,1190	
		Asymptomatic non-hospitalised COVID-19	-,10809	,07767	,498	-,2961	,0799	
Fourth_ventricle_percentile	Control Group	Asymptomatic non-hospitalised COVID-19	-6,03915	5,58204	,843	-19,5520	7,4737	
		Severe hospitalised COVID-19	-,48155	5,67260	1,000	-14,2136	13,2505	
	Asymptomatic non-hospitalised COVID-19	Control Group	6,03915	5,58204	,843	-7,4737	19,5520	
		Severe hospitalised COVID-19	5,55760	5,79952	1,000	-8,4817	19,5969	
	Severe hospitalised COVID-19	Control Group	,48155	5,67260	1,000	-13,2505	14,2136	
		Asymptomatic non-hospitalised COVID-19	-5,55760	5,79952	1,000	-19,5969	8,4817	

*. The mean difference is significant at the 0.05 level.	
