# Supplementary material for: Brain Volume Changes after COVID-19 Compared to Healthy Controls by Artificial Intelligence-Based MRI Volumetry
Source: Diagnostics (Basel). 2023 May 12;13(10):1716. doi: 10.3390/diagnostics13101716 (PMC10216908; doi:10.3390/diagnostics13101716)
Supplement: Supplementary file 1 [file diagnostics-13-01716-s001.zip › Supplemental Table S2.doc]

Table S2. Pairwise analyses ex ICU


Descriptives	
	N	Mean	Std. Deviation	Std. Error	95% Confidence Interval for Mean	Minimum	Maximum	
					Lower Bound	Upper Bound			
Whole_brain_volume	Control Group	56	1264,1768	117,28260	15,67255	1232,7683	1295,5853	1007,00	1551,20	
	Asymptomatic non-hospitalised COVID-19	51	1298,1882	143,62517	20,11155	1257,7930	1338,5835	1018,00	1626,60	
	Severe hospitalised COVID-19	39	1207,0103	113,38323	18,15585	1170,2557	1243,7648	952,00	1452,00	
	Total	146	1260,7870	130,28444	10,78242	1239,4760	1282,0980	952,00	1626,60	
Whole_brain_precentile	Control Group	56	82,0929	17,40070	2,32527	77,4329	86,7528	27,30	99,40	
	Asymptomatic non-hospitalised COVID-19	51	80,5608	21,50755	3,01166	74,5117	86,6099	16,00	100,00	
	Severe hospitalised COVID-19	39	74,1103	25,05796	4,01249	65,9874	82,2331	8,00	100,00	
	Total	146	79,4253	21,20639	1,75505	75,9565	82,8941	8,00	100,00	
Whole_brain_white_substance	Control Group	56	554,2429	61,30981	8,19287	537,8240	570,6617	400,00	729,90	
	Asymptomatic non-hospitalised COVID-19	51	571,7882	74,26375	10,39900	550,9012	592,6753	440,00	773,30	
	Severe hospitalised COVID-19	39	535,3718	57,61222	9,22534	516,6961	554,0475	382,00	649,00	
	Total	146	555,3308	66,33449	5,48988	544,4803	566,1814	382,00	773,30	
Whole_brain_white_substance_percentile	Control Group	56	85,8214	16,02419	2,14132	81,5301	90,1127	33,00	100,00	
	Asymptomatic non-hospitalised COVID-19	51	85,8431	16,67326	2,33472	81,1537	90,5326	36,40	100,00	
	Severe hospitalised COVID-19	39	83,8897	22,99517	3,68217	76,4356	91,3439	14,00	100,00	
	Total	146	85,3130	18,23673	1,50928	82,3300	88,2960	14,00	100,00	
Whole_brain_grey_substance	Control Group	56	709,8250	64,35361	8,59961	692,5910	727,0590	603,20	888,00	
	Asymptomatic non-hospitalised COVID-19	51	726,8706	79,02982	11,06639	704,6431	749,0981	579,00	896,00	
	Severe hospitalised COVID-19	39	667,6846	72,17429	11,55714	644,2884	691,0808	450,00	803,00	
	Total	146	704,5226	75,10814	6,21599	692,2369	716,8083	450,00	896,00	
Whole_brain_grey_substance_percentile	Control Group	56	56,1375	24,22742	3,23753	49,6494	62,6256	3,00	98,00	
	Asymptomatic non-hospitalised COVID-19	51	53,1824	26,60188	3,72501	45,7005	60,6643	4,00	99,40	
	Severe hospitalised COVID-19	39	44,3615	26,62411	4,26327	35,7310	52,9921	7,00	94,00	
	Total	146	51,9596	25,98507	2,15054	47,7091	56,2100	3,00	99,40	
Supratentorial_gross_cerebral_cortex_volume	Control Group	56	483,6821	46,01472	6,14898	471,3593	496,0050	395,00	604,00	
	Asymptomatic non-hospitalised COVID-19	51	491,1745	58,25246	8,15698	474,7907	507,5583	381,00	611,00	
	Severe hospitalised COVID-19	39	455,9846	47,11653	7,54468	440,7112	471,2580	358,00	556,90	
	Total	146	478,9007	52,51763	4,34639	470,3102	487,4911	358,00	611,00	
Supratentorial_cerebral_cortex_percentile	Control Group	56	46,4929	25,97637	3,47124	39,5363	53,4494	3,00	99,00	
	Asymptomatic non-hospitalised COVID-19	51	40,3471	26,02458	3,64417	33,0275	47,6666	1,60	99,70	
	Severe hospitalised COVID-19	39	36,4282	27,40978	4,38908	27,5430	45,3134	3,00	87,00	
	Total	146	41,6575	26,52117	2,19491	37,3194	45,9957	1,60	99,70	
Frontal_right_volume	Control Group	56	91,2768	9,71660	1,29844	88,6747	93,8789	69,30	122,00	
	Asymptomatic non-hospitalised COVID-19	51	92,8137	11,20030	1,56836	89,6636	95,9639	69,00	117,00	
	Severe hospitalised COVID-19	39	85,6641	9,58605	1,53500	82,5567	88,7715	70,00	112,00	
	Total	146	90,3144	10,56042	,87399	88,5870	92,0418	69,00	122,00	
Frontal_right_percentile	Control Group	56	55,3071	26,81456	3,58325	48,1262	62,4881	3,00	98,00	
	Asymptomatic non-hospitalised COVID-19	51	46,8412	27,66534	3,87392	39,0602	54,6222	,10	99,80	
	Severe hospitalised COVID-19	39	43,4359	28,21608	4,51819	34,2893	52,5825	5,00	99,10	
	Total	146	49,1788	27,75961	2,29740	44,6380	53,7195	,10	99,80	
Frontal_left_volume	Control Group	56	87,5196	9,04657	1,20890	85,0970	89,9423	70,60	116,00	
	Asymptomatic non-hospitalised COVID-19	51	88,2235	13,72644	1,92209	84,3629	92,0842	31,00	115,00	
	Severe hospitalised COVID-19	39	82,4590	9,43788	1,51127	79,3996	85,5184	67,00	104,40	
	Total	146	86,4137	11,18875	,92599	84,5835	88,2439	31,00	116,00	
Frontal_left_percentile	Control Group	56	48,4071	25,52765	3,41128	41,5708	55,2435	1,00	97,00	
	Asymptomatic non-hospitalised COVID-19	51	43,4804	27,30055	3,82284	35,8020	51,1588	,10	99,30	
	Severe hospitalised COVID-19	39	39,7077	28,09733	4,49917	30,5996	48,8158	1,60	95,00	
	Total	146	44,3623	26,89798	2,22609	39,9625	48,7621	,10	99,30	
Parietal_right_volume	Control Group	56	48,0929	5,12388	,68471	46,7207	49,4650	39,00	60,00	
	Asymptomatic non-hospitalised COVID-19	51	48,5275	5,87156	,82218	46,8760	50,1789	38,00	62,40	
	Severe hospitalised COVID-19	39	45,0359	5,08737	,81463	43,3868	46,6850	34,00	57,00	
	Total	146	47,4281	5,54654	,45903	46,5208	48,3353	34,00	62,40	
Parietal_right_percentile	Control Group	56	34,1643	21,88769	2,92487	28,3027	40,0258	3,00	96,00	
	Asymptomatic non-hospitalised COVID-19	51	26,2667	21,08900	2,95305	20,3353	32,1980	,10	88,40	
	Severe hospitalised COVID-19	39	28,1487	22,99617	3,68233	20,6942	35,6032	,30	89,00	
	Total	146	29,7986	22,04872	1,82477	26,1921	33,4052	,10	96,00	
Parietal_left_volume	Control Group	56	50,2875	5,43765	,72664	48,8313	51,7437	38,00	61,20	
	Asymptomatic non-hospitalised COVID-19	51	50,6314	6,01184	,84183	48,9405	52,3222	39,80	63,00	
	Severe hospitalised COVID-19	39	46,8487	5,19761	,83228	45,1638	48,5336	35,00	59,00	
	Total	146	49,4890	5,77394	,47785	48,5446	50,4335	35,00	63,00	
Parietal_left_percentile	Control Group	56	46,4357	25,25666	3,37506	39,6719	53,1995	5,00	99,00	
	Asymptomatic non-hospitalised COVID-19	51	36,9137	23,87354	3,34296	30,1992	43,6283	,20	89,20	
	Severe hospitalised COVID-19	39	35,4410	24,82426	3,97506	27,3939	43,4881	1,50	87,00	
	Total	146	40,1726	24,99799	2,06885	36,0836	44,2616	,20	99,00	
Precuneus_right_volume	Control Group	56	11,4357	1,44555	,19317	11,0486	11,8228	8,40	14,10	
	Asymptomatic non-hospitalised COVID-19	51	11,6627	1,59762	,22371	11,2134	12,1121	8,90	14,80	
	Severe hospitalised COVID-19	39	10,7385	1,35426	,21685	10,2995	11,1775	7,90	12,90	
	Total	146	11,3288	1,51352	,12526	11,0812	11,5763	7,90	14,80	
Precuneus_right_percentile	Control Group	56	47,7804	29,91054	3,99696	39,7703	55,7905	3,00	95,60	
	Asymptomatic non-hospitalised COVID-19	51	45,6784	26,00076	3,64084	38,3656	52,9913	,10	98,50	
	Severe hospitalised COVID-19	39	43,6410	27,33623	4,37730	34,7796	52,5024	,80	90,50	
	Total	146	45,9404	27,76784	2,29808	41,3983	50,4825	,10	98,50	
Precuneus_left_volume	Control Group	56	11,9130	2,04552	,27334	11,3652	12,4608	1,03	14,60	
	Asymptomatic non-hospitalised COVID-19	51	12,1647	1,76009	,24646	11,6697	12,6597	8,90	15,80	
	Severe hospitalised COVID-19	39	11,2410	1,70567	,27313	10,6881	11,7939	7,80	14,70	
	Total	146	11,8214	1,88496	,15600	11,5131	12,1298	1,03	15,80	
Precuneus_left_percentile	Control Group	56	70,2964	23,33737	3,11859	64,0466	76,5462	11,00	99,90	
	Asymptomatic non-hospitalised COVID-19	51	64,9333	25,73612	3,60378	57,6949	72,1717	9,20	99,50	
	Severe hospitalised COVID-19	39	63,0923	30,44272	4,87474	53,2239	72,9607	1,10	98,80	
	Total	146	66,4986	26,21847	2,16986	62,2100	70,7873	1,10	99,90	
Occipital_right_volume	Control Group	56	31,5232	3,38005	,45168	30,6180	32,4284	23,00	39,60	
	Asymptomatic non-hospitalised COVID-19	51	33,2706	9,39728	1,31588	30,6276	35,9136	21,00	74,00	
	Severe hospitalised COVID-19	39	29,5231	4,11470	,65888	28,1892	30,8569	22,00	40,90	
	Total	146	31,5993	6,43164	,53229	30,5473	32,6514	21,00	74,00	
Occipital_right_percentlile	Control Group	56	23,7018	24,08260	3,21817	17,2524	30,1511	,70	92,50	
	Asymptomatic non-hospitalised COVID-19	51	21,9235	23,72335	3,32193	15,2512	28,5958	,00	85,60	
	Severe hospitalised COVID-19	39	20,0590	22,21782	3,55770	12,8568	27,2612	,00	92,20	
	Total	146	22,1075	23,35691	1,93303	18,2870	25,9281	,00	92,50	
Occipital_left_volume	Control Group	56	37,8321	8,09296	1,08147	35,6648	39,9995	26,00	90,00	
	Asymptomatic non-hospitalised COVID-19	51	37,7902	7,32372	1,02553	35,7304	39,8500	25,00	72,00	
	Severe hospitalised COVID-19	39	34,6436	4,57129	,73199	33,1617	36,1254	25,00	45,40	
	Total	146	36,9658	7,12703	,58984	35,8000	38,1315	25,00	90,00	
Occipital_left_percentile	Control Group	56	40,5107	27,27471	3,64474	33,2065	47,8149	2,20	94,60	
	Asymptomatic non-hospitalised COVID-19	51	38,0569	28,97574	4,05741	29,9073	46,2064	,80	95,60	
	Severe hospitalised COVID-19	39	37,2103	27,98912	4,48185	28,1372	46,2833	3,00	97,90	
	Total	146	38,7719	27,91045	2,30989	34,2065	43,3373	,80	97,90	
Temporal_right_volume	Control Group	56	71,5964	7,20136	,96232	69,6679	73,5250	57,00	87,00	
	Asymptomatic non-hospitalised COVID-19	51	73,8333	9,19973	1,28822	71,2459	76,4208	59,00	93,00	
	Severe hospitalised COVID-19	39	68,2436	7,19637	1,15234	65,9108	70,5764	49,80	83,00	
	Total	146	71,4822	8,19742	,67842	70,1413	72,8231	49,80	93,00	
Temporal_right_percentile	Control Group	56	56,3125	28,07251	3,75135	48,7946	63,8304	4,00	99,00	
	Asymptomatic non-hospitalised COVID-19	51	57,8431	27,53560	3,85576	50,0986	65,5876	7,30	99,90	
	Severe hospitalised COVID-19	39	53,4077	26,35543	4,22025	44,8643	61,9511	6,00	99,20	
	Total	146	56,0712	27,30281	2,25960	51,6052	60,5372	4,00	99,90	
Temporal_left_volume	Control Group	56	66,4643	8,30854	1,11027	64,2392	68,6893	30,00	83,00	
	Asymptomatic non-hospitalised COVID-19	51	68,1980	9,64155	1,35009	65,4863	70,9098	31,00	87,00	
	Severe hospitalised COVID-19	39	63,8179	6,10732	,97795	61,8382	65,7977	51,00	76,80	
	Total	146	66,3630	8,42288	,69708	64,9853	67,7408	30,00	87,00	
Temporal_left_percentlile	Control Group	56	56,8250	30,13031	4,02633	48,7561	64,8939	3,10	99,00	
	Asymptomatic non-hospitalised COVID-19	51	59,4373	27,70386	3,87932	51,6454	67,2291	7,70	99,90	
	Severe hospitalised COVID-19	39	50,1154	25,75369	4,12389	41,7670	58,4638	7,00	97,80	
	Total	146	55,9452	28,22330	2,33578	51,3286	60,5618	3,10	99,90	
Mesiotemporal_right_volume	Control Group	56	23,8571	11,00096	1,47006	20,9111	26,8032	5,00	38,00	
	Asymptomatic non-hospitalised COVID-19	51	26,2843	10,79100	1,51104	23,2493	29,3193	7,10	40,00	
	Severe hospitalised COVID-19	39	22,6667	10,34612	1,65671	19,3128	26,0205	4,00	36,00	
	Total	146	24,3870	10,78338	,89244	22,6231	26,1509	4,00	40,00	
Mesiotemporal_right_percentile	Control Group	56	48,9482	32,89097	4,39524	40,1400	57,7565	8,00	99,00	
	Asymptomatic non-hospitalised COVID-19	51	53,5353	28,83399	4,03757	45,4256	61,6450	12,00	99,00	
	Severe hospitalised COVID-19	39	47,4103	32,11306	5,14220	37,0004	57,8201	8,00	99,00	
	Total	146	50,1397	31,20735	2,58274	45,0350	55,2444	8,00	99,00	
Mesiotemproal_left_volume	Control Group	56	22,4357	10,23394	1,36757	19,6950	25,1764	5,00	36,00	
	Asymptomatic non-hospitalised COVID-19	51	24,6941	9,79190	1,37114	21,9401	27,4481	8,60	39,00	
	Severe hospitalised COVID-19	39	21,2256	9,36993	1,50039	18,1883	24,2630	4,00	33,00	
	Total	146	22,9014	9,88772	,81831	21,2840	24,5187	4,00	39,00	
Mesiotemporal_left_percentile	Control Group	56	48,2054	33,65593	4,49746	39,1922	57,2185	5,00	99,00	
	Asymptomatic non-hospitalised COVID-19	51	52,6118	28,19284	3,94779	44,6824	60,5411	16,00	99,90	
	Severe hospitalised COVID-19	39	46,3154	31,24943	5,00391	36,1855	56,4453	6,00	99,00	
	Total	146	49,2397	31,08640	2,57273	44,1548	54,3246	5,00	99,90	
Hippocampus_right_volume	Control Group	56	5,0214	5,86576	,78385	3,4506	6,5923	3,50	48,00	
	Asymptomatic non-hospitalised COVID-19	51	4,3529	,52853	,07401	4,2043	4,5016	3,60	6,00	
	Severe hospitalised COVID-19	39	4,1359	,38285	,06130	4,0118	4,2600	3,50	5,00	
	Total	146	4,5514	3,65121	,30218	3,9541	5,1486	3,50	48,00	
Hippocampus_right_percentile	Control Group	56	56,1393	26,79240	3,58028	48,9642	63,3143	1,00	98,60	
	Asymptomatic non-hospitalised COVID-19	51	57,4255	28,65053	4,01188	49,3674	65,4836	,30	99,00	
	Severe hospitalised COVID-19	39	55,1077	26,33312	4,21667	46,5715	63,6439	7,90	95,00	
	Total	146	56,3130	27,16430	2,24813	51,8697	60,7564	,30	99,00	
Hippocampus_left_volume	Control Group	56	5,5429	10,69430	1,42909	2,6789	8,4068	3,20	84,00	
	Asymptomatic non-hospitalised COVID-19	51	4,8686	3,78690	,53027	3,8035	5,9337	3,40	31,00	
	Severe hospitalised COVID-19	39	4,0769	,55130	,08828	3,8982	4,2556	3,10	5,00	
	Total	146	4,9158	6,98194	,57783	3,7737	6,0578	3,10	84,00	
Hippocampus_left_percentile	Control Group	56	58,8643	26,40656	3,52872	51,7926	65,9360	6,80	99,80	
	Asymptomatic non-hospitalised COVID-19	51	59,1882	27,21394	3,81071	51,5342	66,8423	8,30	99,00	
	Severe hospitalised COVID-19	39	58,7308	25,03745	4,00920	50,6146	66,8470	6,70	95,00	
	Total	146	58,9418	26,15711	2,16478	54,6632	63,2204	6,70	99,80	
Gyrus_parahippocampalis_right_volume	Control Group	56	3,2732	,37440	,05003	3,1729	3,3735	2,50	4,10	
	Asymptomatic non-hospitalised COVID-19	51	3,4000	,43726	,06123	3,2770	3,5230	2,50	4,30	
	Severe hospitalised COVID-19	39	3,2103	,33308	,05334	3,1023	3,3182	2,50	4,10	
	Total	146	3,3007	,39260	,03249	3,2365	3,3649	2,50	4,30	
Gyrus_parahippocampalis_right_percentile	Control Group	56	62,8321	28,40811	3,79619	55,2244	70,4399	3,30	99,90	
	Asymptomatic non-hospitalised COVID-19	51	69,8824	28,60626	4,00568	61,8367	77,9280	1,40	99,90	
	Severe hospitalised COVID-19	39	69,3436	23,42129	3,75041	61,7513	76,9359	22,40	99,90	
	Total	146	67,0342	27,26090	2,25613	62,5751	71,4934	1,40	99,90	
Gyrus_parahippocampalis_left_volume	Control Group	56	3,4089	,33044	,04416	3,3204	3,4974	2,60	4,20	
	Asymptomatic non-hospitalised COVID-19	51	3,5490	,36790	,05152	3,4455	3,6525	2,80	4,30	
	Severe hospitalised COVID-19	39	3,2974	,28515	,04566	3,2050	3,3899	2,70	4,00	
	Total	146	3,4281	,34536	,02858	3,3716	3,4846	2,60	4,30	
Gyrus_parahippocampalis_left_percentile	Control Group	56	60,6571	27,73297	3,70597	53,2302	68,0841	6,10	99,80	
	Asymptomatic non-hospitalised COVID-19	51	71,5098	23,26589	3,25788	64,9662	78,0534	18,80	99,90	
	Severe hospitalised COVID-19	39	61,6000	26,29032	4,20982	53,0777	70,1223	3,80	98,10	
	Total	146	64,7000	26,16744	2,16563	60,4197	68,9803	3,80	99,90	
Regio_entorhinalis_right_volume	Control Group	56	2,5054	,31008	,04144	2,4223	2,5884	1,90	3,20	
	Asymptomatic non-hospitalised COVID-19	51	2,5961	,32060	,04489	2,5059	2,6862	2,10	3,30	
	Severe hospitalised COVID-19	39	2,4487	,24802	,03971	2,3683	2,5291	2,00	3,20	
	Total	146	2,5219	,30252	,02504	2,4724	2,5714	1,90	3,30	
Regio_entorhinalis_right_percentile	Control Group	56	70,4643	26,89721	3,59429	63,2612	77,6674	3,90	99,70	
	Asymptomatic non-hospitalised COVID-19	51	75,3706	21,56478	3,01967	69,3054	81,4358	20,50	99,90	
	Severe hospitalised COVID-19	39	72,2103	19,97399	3,19840	65,7354	78,6851	29,00	99,80	
	Total	146	72,6445	23,32016	1,92999	68,8300	76,4591	3,90	99,90	
Regio_entorhinalis_left_volume	Control Group	56	2,4536	,27632	,03692	2,3796	2,5276	1,90	3,30	
	Asymptomatic non-hospitalised COVID-19	51	2,5176	,31222	,04372	2,4298	2,6055	1,60	3,10	
	Severe hospitalised COVID-19	39	2,4077	,29858	,04781	2,3109	2,5045	1,90	3,40	
	Total	146	2,4637	,29639	,02453	2,4152	2,5122	1,60	3,40	
Regio_entorhinalis_left_percentile	Control Group	56	59,1143	26,74015	3,57330	51,9532	66,2753	2,50	99,90	
	Asymptomatic non-hospitalised COVID-19	51	63,9922	26,22516	3,67226	56,6162	71,3681	2,50	97,70	
	Severe hospitalised COVID-19	39	58,5513	26,57122	4,25480	49,9379	67,1647	7,60	99,90	
	Total	146	60,6678	26,44676	2,18875	56,3418	64,9938	2,50	99,90	
Nucleus_caudatus_right_volume	Control Group	56	3,3000	,42725	,05709	3,1856	3,4144	2,40	4,50	
	Asymptomatic non-hospitalised COVID-19	51	3,4882	,39982	,05599	3,3758	3,6007	2,70	4,50	
	Severe hospitalised COVID-19	39	3,1846	,39772	,06369	3,0557	3,3135	2,50	4,20	
	Total	146	3,3349	,42501	,03517	3,2654	3,4045	2,40	4,50	
Nucleus_caudatus_right_percentile	Control Group	56	40,4964	25,81606	3,44982	33,5828	47,4100	2,20	99,40	
	Asymptomatic non-hospitalised COVID-19	51	52,1902	24,97050	3,49657	45,1671	59,2133	4,50	96,60	
	Severe hospitalised COVID-19	39	40,6385	25,69235	4,11407	32,3100	48,9670	3,60	96,20	
	Total	146	44,6192	25,91883	2,14506	40,3796	48,8588	2,20	99,40	
Nucleus_caudatus_left_volume	Control Group	56	2,9268	,37537	,05016	2,8263	3,0273	2,10	4,10	
	Asymptomatic non-hospitalised COVID-19	51	3,1490	,35178	,04926	3,0501	3,2480	2,40	3,80	
	Severe hospitalised COVID-19	39	2,8359	,38147	,06108	2,7122	2,9596	2,10	3,60	
	Total	146	2,9801	,38856	,03216	2,9166	3,0437	2,10	4,10	
Nucleus_caudatus_left_percentile	Control Group	56	27,3679	22,08103	2,95070	21,4545	33,2812	1,00	86,40	
	Asymptomatic non-hospitalised COVID-19	51	39,5078	23,30756	3,26371	32,9525	46,0632	,20	93,50	
	Severe hospitalised COVID-19	39	27,8128	24,00671	3,84415	20,0307	35,5949	,50	84,80	
	Total	146	31,7274	23,58083	1,95156	27,8702	35,5846	,20	93,50	
Putamen_right_volume	Control Group	56	4,1893	,45793	,06119	4,0667	4,3119	3,30	5,30	
	Asymptomatic non-hospitalised COVID-19	51	4,3294	,50884	,07125	4,1863	4,4725	3,10	5,80	
	Severe hospitalised COVID-19	39	4,0462	,50463	,08081	3,8826	4,2097	3,00	5,40	
	Total	146	4,2000	,49786	,04120	4,1186	4,2814	3,00	5,80	
Putamen_right_percentile	Control Group	56	27,6875	22,35835	2,98776	21,6999	33,6751	,10	92,30	
	Asymptomatic non-hospitalised COVID-19	51	31,9510	24,27669	3,39942	25,1231	38,7789	,20	91,80	
	Severe hospitalised COVID-19	39	28,4128	26,64048	4,26589	19,7770	37,0487	,10	95,80	
	Total	146	29,3705	24,13544	1,99746	25,4226	33,3185	,10	95,80	
Putamen_left_volume	Control Group	56	4,3071	,47246	,06314	4,1806	4,4337	3,40	5,50	
	Asymptomatic non-hospitalised COVID-19	51	4,4510	,55113	,07717	4,2960	4,6060	3,20	6,10	
	Severe hospitalised COVID-19	39	4,1410	,49509	,07928	3,9805	4,3015	3,20	5,40	
	Total	146	4,3130	,51799	,04287	4,2283	4,3977	3,20	6,10	
Putamen_left_percentile	Control Group	56	29,7125	24,41752	3,26293	23,1734	36,2516	,10	81,70	
	Asymptomatic non-hospitalised COVID-19	51	33,8353	25,36712	3,55211	26,7007	40,9699	,10	96,60	
	Severe hospitalised COVID-19	39	27,8513	24,17376	3,87090	20,0151	35,6875	,10	92,70	
	Total	146	30,6555	24,64151	2,03935	26,6248	34,6862	,10	96,60	
Pallidum_right_volume	Control Group	56	1,4268	,17108	,02286	1,3810	1,4726	1,10	2,00	
	Asymptomatic non-hospitalised COVID-19	51	1,4725	,15110	,02116	1,4301	1,5150	1,10	1,80	
	Severe hospitalised COVID-19	39	1,3667	,14388	,02304	1,3200	1,4133	1,00	1,60	
	Total	146	1,4267	,16158	,01337	1,4003	1,4531	1,00	2,00	
Pallidum_right_percentile	Control Group	56	36,6536	27,64327	3,69399	29,2507	44,0565	1,30	98,10	
	Asymptomatic non-hospitalised COVID-19	51	40,7314	25,43717	3,56192	33,5771	47,8857	,40	92,40	
	Severe hospitalised COVID-19	39	34,3154	28,13196	4,50472	25,1961	43,4347	,90	97,80	
	Total	146	37,4534	26,96384	2,23154	33,0429	41,8640	,40	98,10	
Pallidum_left_volume	Control Group	56	1,3804	,15305	,02045	1,3394	1,4213	1,10	1,90	
	Asymptomatic non-hospitalised COVID-19	51	1,4412	,15770	,02208	1,3968	1,4855	1,10	1,90	
	Severe hospitalised COVID-19	39	1,3231	,14412	,02308	1,2764	1,3698	1,00	1,60	
	Total	146	1,3863	,15828	,01310	1,3604	1,4122	1,00	1,90	
Pallidum_left_percentile	Control Group	56	27,0946	23,85160	3,18730	20,7071	33,4821	,10	86,10	
	Asymptomatic non-hospitalised COVID-19	51	28,5137	22,19231	3,10754	22,2720	34,7554	,60	81,60	
	Severe hospitalised COVID-19	39	22,8795	24,09471	3,85824	15,0689	30,6901	,10	80,80	
	Total	146	26,4644	23,29912	1,92825	22,6533	30,2755	,10	86,10	
Thalamus_right_volume	Control Group	56	8,1286	,75552	,10096	7,9262	8,3309	6,80	10,40	
	Asymptomatic non-hospitalised COVID-19	51	8,3471	,81126	,11360	8,1189	8,5752	6,40	10,00	
	Severe hospitalised COVID-19	39	7,7205	,80530	,12895	7,4595	7,9816	6,20	9,40	
	Total	146	8,0959	,82092	,06794	7,9616	8,2302	6,20	10,40	
Thalamus_right_percentile	Control Group	56	44,4339	30,42709	4,06599	36,2855	52,5824	,20	93,80	
	Asymptomatic non-hospitalised COVID-19	51	44,1392	28,16849	3,94438	36,2167	52,0617	,60	99,80	
	Severe hospitalised COVID-19	39	35,1333	29,07749	4,65612	25,7075	44,5592	,10	94,90	
	Total	146	41,8466	29,37536	2,43112	37,0416	46,6516	,10	99,80	
Thalamus_left_volume	Control Group	56	8,4571	,81058	,10832	8,2401	8,6742	7,30	10,80	
	Asymptomatic non-hospitalised COVID-19	51	8,4647	1,35216	,18934	8,0844	8,8450	1,40	10,50	
	Severe hospitalised COVID-19	39	8,0410	,82803	,13259	7,7726	8,3094	6,20	9,90	
	Total	146	8,3486	1,04599	,08657	8,1775	8,5197	1,40	10,80	
Thalamus_left_percentile	Control Group	56	56,7321	29,84449	3,98814	48,7397	64,7246	,70	97,90	
	Asymptomatic non-hospitalised COVID-19	51	54,3294	26,68210	3,73624	46,8249	61,8339	8,50	99,90	
	Severe hospitalised COVID-19	39	45,9846	29,30056	4,69184	36,4865	55,4828	2,60	97,90	
	Total	146	53,0219	28,76759	2,38082	48,3163	57,7275	,70	99,90	
Brainstem_volume	Control Group	56	27,8714	10,30650	1,37726	25,1113	30,6315	21,00	99,00	
	Asymptomatic non-hospitalised COVID-19	51	26,3882	2,91819	,40863	25,5675	27,2090	21,00	32,20	
	Severe hospitalised COVID-19	39	24,8308	2,70138	,43257	23,9551	25,7065	20,00	30,30	
	Total	146	26,5411	6,82783	,56508	25,4242	27,6579	20,00	99,00	
Brainstem_percentile	Control Group	56	55,9107	24,96579	3,33619	49,2248	62,5966	8,00	99,90	
	Asymptomatic non-hospitalised COVID-19	51	56,3451	28,31822	3,96534	48,3805	64,3097	1,00	99,00	
	Severe hospitalised COVID-19	39	47,8436	26,27609	4,20754	39,3259	56,3613	2,80	97,20	
	Total	146	53,9075	26,59730	2,20121	49,5569	58,2581	1,00	99,90	
Mesencephalon_volume	Control Group	56	8,9107	12,28785	1,64203	5,6200	12,2014	6,00	99,00	
	Asymptomatic non-hospitalised COVID-19	51	7,2314	,89632	,12551	6,9793	7,4835	6,00	9,30	
	Severe hospitalised COVID-19	39	6,8205	,87425	,13999	6,5371	7,1039	5,00	8,60	
	Total	146	7,7658	7,65486	,63352	6,5136	9,0179	5,00	99,00	
Mesencephalon_percentile	Control Group	56	49,4179	27,36541	3,65686	42,0894	56,7464	4,00	99,00	
	Asymptomatic non-hospitalised COVID-19	51	49,8216	28,56335	3,99967	41,7880	57,8551	3,00	98,00	
	Severe hospitalised COVID-19	39	41,1692	26,98072	4,32037	32,4231	49,9154	1,70	94,40	
	Total	146	47,3555	27,75312	2,29686	42,8158	51,8951	1,70	99,00	
Pons_volume	Control Group	56	15,8554	11,17777	1,49369	12,8619	18,8488	11,00	97,00	
	Asymptomatic non-hospitalised COVID-19	51	14,3588	1,76637	,24734	13,8620	14,8556	11,00	18,00	
	Severe hospitalised COVID-19	39	13,5718	1,49013	,23861	13,0887	14,0548	11,00	17,00	
	Total	146	14,7226	7,06740	,58490	13,5666	15,8786	11,00	97,00	
Pons_percentile	Control Group	56	54,0107	27,00505	3,60870	46,7787	61,2427	3,00	122,00	
	Asymptomatic non-hospitalised COVID-19	51	51,1471	29,23668	4,09395	42,9241	59,3700	,10	98,00	
	Severe hospitalised COVID-19	39	45,3718	26,59763	4,25903	36,7498	53,9937	2,80	98,00	
	Total	146	50,7027	27,72561	2,29459	46,1676	55,2379	,10	122,00	
Cerebellar_grey_matter_volume	Control Group	56	109,6161	11,10947	1,48457	106,6409	112,5912	88,00	138,00	
	Asymptomatic non-hospitalised COVID-19	51	112,5157	11,18308	1,56594	109,3704	115,6610	91,00	141,00	
	Severe hospitalised COVID-19	39	104,7487	11,44159	1,83212	101,0398	108,4577	84,00	128,80	
	Total	146	109,3288	11,55391	,95621	107,4389	111,2187	84,00	141,00	
Cerebellar_grey_matter_percentile	Control Group	56	68,0143	24,56272	3,28233	61,4363	74,5922	4,00	99,00	
	Asymptomatic non-hospitalised COVID-19	51	70,4529	25,59591	3,58414	63,2540	77,6519	8,00	100,00	
	Severe hospitalised COVID-19	39	58,7641	27,74859	4,44333	49,7690	67,7592	7,00	99,70	
	Total	146	66,3952	26,05797	2,15657	62,1328	70,6576	4,00	100,00	
Left_ventricle_volume	Control Group	56	10,0946	7,45960	,99683	8,0969	12,0923	2,00	43,40	
	Asymptomatic non-hospitalised COVID-19	51	8,5078	5,92246	,82931	6,8421	10,1736	2,00	30,70	
	Severe hospitalised COVID-19	39	7,8718	4,04091	,64706	6,5619	9,1817	2,00	23,00	
	Total	146	8,9466	6,19425	,51264	7,9334	9,9598	2,00	43,40	
Left_ventricle_percentile	Control Group	56	54,3732	31,06270	4,15093	46,0546	62,6919	1,00	100,00	
	Asymptomatic non-hospitalised COVID-19	51	48,4804	29,30734	4,10385	40,2376	56,7232	2,00	99,00	
	Severe hospitalised COVID-19	39	51,9051	26,45941	4,23690	43,3280	60,4823	3,00	95,00	
	Total	146	51,6555	29,19011	2,41579	46,8808	56,4302	1,00	100,00	
Right_ventricle_volume	Control Group	56	9,9071	6,20823	,82961	8,2446	11,5697	2,00	31,50	
	Asymptomatic non-hospitalised COVID-19	51	9,0118	5,55070	,77725	7,4506	10,5729	1,70	28,30	
	Severe hospitalised COVID-19	39	8,3487	3,76549	,60296	7,1281	9,5693	2,00	19,80	
	Total	146	9,1781	5,41844	,44843	8,2918	10,0644	1,70	31,50	
Right_ventricle_percentile	Control Group	56	54,6679	31,63840	4,22786	46,1950	63,1407	1,00	99,60	
	Asymptomatic non-hospitalised COVID-19	51	51,0784	29,46008	4,12524	42,7927	59,3642	1,90	99,00	
	Severe hospitalised COVID-19	39	52,2513	29,26419	4,68602	42,7649	61,7376	1,00	96,00	
	Total	146	52,7685	30,09757	2,49089	47,8453	57,6916	1,00	99,60	
Third_ventricle_volume	Control Group	56	,6875	,40679	,05436	,5786	,7964	,10	1,90	
	Asymptomatic non-hospitalised COVID-19	51	,7608	,39399	,05517	,6500	,8716	,00	1,50	
	Severe hospitalised COVID-19	39	,7462	,36044	,05772	,6293	,8630	,00	1,80	
	Total	146	,7288	,38916	,03221	,6651	,7924	,00	1,90	
Third_ventricle_percentile	Control Group	56	48,6429	29,00896	3,87649	40,8742	56,4115	4,00	99,80	
	Asymptomatic non-hospitalised COVID-19	51	43,4725	29,44218	4,12273	35,1918	51,7533	,80	98,00	
	Severe hospitalised COVID-19	39	54,1718	26,93551	4,31313	45,4403	62,9033	5,00	100,00	
	Total	146	48,3137	28,73604	2,37821	43,6133	53,0141	,80	100,00	
Fourth_ventricle_volume	Control Group	56	1,1607	,36813	,04919	1,0621	1,2593	,70	2,00	
	Asymptomatic non-hospitalised COVID-19	51	1,2039	,42472	,05947	1,0845	1,3234	,50	2,80	
	Severe hospitalised COVID-19	39	1,0538	,33705	,05397	,9446	1,1631	,00	2,00	
	Total	146	1,1473	,38331	,03172	1,0846	1,2100	,00	2,80	
Fourth_ventricle_percentile	Control Group	56	48,3393	28,67372	3,83169	40,6604	56,0182	3,80	100,00	
	Asymptomatic non-hospitalised COVID-19	51	54,3784	27,16137	3,80335	46,7392	62,0177	6,70	100,00	
	Severe hospitalised COVID-19	39	46,7487	30,55337	4,89245	36,8445	56,6530	5,00	100,00	
	Total	146	50,0240	28,66268	2,37214	45,3355	54,7124	3,80	100,00	


ANOVA	
	Sum of Squares	df	Mean Square	F	Sig.	
Whole_brain_volume	Between Groups	184770,537	2	92385,268	5,803	,004	
	Within Groups	2276464,709	143	15919,334			
	Total	2461235,245	145				
Whole_brain_precentile	Between Groups	1565,982	2	782,991	1,759	,176	
	Within Groups	63642,135	143	445,050			
	Total	65208,116	145				
Whole_brain_white_substance	Between Groups	29415,602	2	14707,801	3,456	,034	
	Within Groups	608622,729	143	4256,103			
	Total	638038,331	145				
Whole_brain_white_substance_percentile	Between Groups	107,810	2	53,905	,160	,852	
	Within Groups	48116,055	143	336,476			
	Total	48223,865	145				
Whole_brain_grey_substance	Between Groups	79969,974	2	39984,987	7,748	,001	
	Within Groups	738008,762	143	5160,900			
	Total	817978,735	145				
Whole_brain_grey_substance_percentile	Between Groups	3305,214	2	1652,607	2,498	,086	
	Within Groups	94602,278	143	661,554			
	Total	97907,492	145				
Supratentorial_gross_cerebral_cortex_volume	Between Groups	29443,980	2	14721,990	5,682	,004	
	Within Groups	370480,710	143	2590,774			
	Total	399924,690	145				
Supratentorial_cerebral_cortex_percentile	Between Groups	2463,374	2	1231,687	1,770	,174	
	Within Groups	99525,623	143	695,983			
	Total	101988,997	145				
Frontal_right_volume	Between Groups	1213,830	2	606,915	5,803	,004	
	Within Groups	14956,930	143	104,594			
	Total	16170,760	145				
Frontal_right_percentile	Between Groups	3668,114	2	1834,057	2,427	,092	
	Within Groups	108068,290	143	755,722			
	Total	111736,404	145				
Frontal_left_volume	Between Groups	845,498	2	422,749	3,493	,033	
	Within Groups	17306,775	143	121,026			
	Total	18152,273	145				
Frontal_left_percentile	Between Groups	1800,818	2	900,409	1,249	,290	
	Within Groups	103106,845	143	721,027			
	Total	104907,663	145				
Parietal_right_volume	Between Groups	309,566	2	154,783	5,332	,006	
	Within Groups	4151,228	143	29,030			
	Total	4460,795	145				
Parietal_right_percentile	Between Groups	1809,680	2	904,840	1,884	,156	
	Within Groups	68681,499	143	480,290			
	Total	70491,180	145				
Parietal_left_volume	Between Groups	374,134	2	187,067	5,998	,003	
	Within Groups	4459,928	143	31,188			
	Total	4834,062	145				
Parietal_left_percentile	Between Groups	3611,447	2	1805,724	2,968	,055	
	Within Groups	86999,003	143	608,385			
	Total	90610,450	145				
Precuneus_right_volume	Between Groups	19,919	2	9,960	4,561	,012	
	Within Groups	312,240	143	2,183			
	Total	332,159	145				
Precuneus_right_percentile	Between Groups	399,283	2	199,641	,256	,774	
	Within Groups	111403,429	143	779,045			
	Total	111802,712	145				
Precuneus_left_volume	Between Groups	19,618	2	9,809	2,830	,062	
	Within Groups	495,578	143	3,466			
	Total	515,196	145				
Precuneus_left_percentile	Between Groups	1385,179	2	692,590	1,008	,368	
	Within Groups	98289,040	143	687,336			
	Total	99674,220	145				
Occipital_right_volume	Between Groups	310,895	2	155,447	3,909	,022	
	Within Groups	5687,175	143	39,770			
	Total	5998,070	145				
Occipital_right_percentlile	Between Groups	307,726	2	153,863	,279	,757	
	Within Groups	78796,316	143	551,023			
	Total	79104,042	145				
Occipital_left_volume	Between Groups	287,006	2	143,503	2,899	,058	
	Within Groups	7078,203	143	49,498			
	Total	7365,209	145				
Occipital_left_percentile	Between Groups	290,500	2	145,250	,184	,832	
	Within Groups	112663,555	143	787,857			
	Total	112954,055	145				
Temporal_right_volume	Between Groups	691,705	2	345,853	5,464	,005	
	Within Groups	9051,969	143	63,300			
	Total	9743,674	145				
Temporal_right_percentile	Between Groups	440,065	2	220,033	,292	,747	
	Within Groups	107649,214	143	752,792			
	Total	108089,279	145				
Temporal_left_volume	Between Groups	424,924	2	212,462	3,081	,049	
	Within Groups	9862,096	143	68,966			
	Total	10287,020	145				
Temporal_left_percentlile	Between Groups	1990,747	2	995,373	1,254	,288	
	Within Groups	113509,715	143	793,774			
	Total	115500,462	145				
Mesiotemporal_right_volume	Between Groups	314,734	2	157,367	1,360	,260	
	Within Groups	16546,051	143	115,707			
	Total	16860,785	145				
Mesiotemporal_right_percentile	Between Groups	958,077	2	479,039	,488	,615	
	Within Groups	140257,252	143	980,820			
	Total	141215,330	145				
Mesiotemproal_left_volume	Between Groups	285,569	2	142,784	1,470	,233	
	Within Groups	13890,651	143	97,137			
	Total	14176,220	145				
Mesiotemporal_left_percentile	Between Groups	973,337	2	486,669	,500	,608	
	Within Groups	139149,512	143	973,074			
	Total	140122,850	145				
Hippocampus_right_volume	Between Groups	21,114	2	10,557	,790	,456	
	Within Groups	1911,931	143	13,370			
	Total	1933,045	145				
Hippocampus_right_percentile	Between Groups	121,467	2	60,734	,081	,922	
	Within Groups	106873,898	143	747,370			
	Total	106995,365	145				
Hippocampus_left_volume	Between Groups	49,578	2	24,789	,505	,605	
	Within Groups	7018,816	143	49,083			
	Total	7068,394	145				
Hippocampus_left_percentile	Between Groups	5,171	2	2,585	,004	,996	
	Within Groups	99202,985	143	693,727			
	Total	99208,155	145				
Gyrus_parahippocampalis_right_volume	Between Groups	,864	2	,432	2,876	,060	
	Within Groups	21,486	143	,150			
	Total	22,350	145				
Gyrus_parahippocampalis_right_percentile	Between Groups	1610,517	2	805,258	1,085	,341	
	Within Groups	106147,192	143	742,288			
	Total	107757,709	145				
Gyrus_parahippocampalis_left_volume	Between Groups	1,432	2	,716	6,455	,002	
	Within Groups	15,863	143	,111			
	Total	17,295	145				
Gyrus_parahippocampalis_left_percentile	Between Groups	3655,138	2	1827,569	2,733	,068	
	Within Groups	95631,422	143	668,751			
	Total	99286,560	145				
Regio_entorhinalis_right_volume	Between Groups	,505	2	,252	2,828	,062	
	Within Groups	12,765	143	,089			
	Total	13,270	145				
Regio_entorhinalis_right_percentile	Between Groups	652,550	2	326,275	,597	,552	
	Within Groups	78202,770	143	546,873			
	Total	78855,321	145				
Regio_entorhinalis_left_volume	Between Groups	,277	2	,138	1,587	,208	
	Within Groups	12,461	143	,087			
	Total	12,738	145				
Regio_entorhinalis_left_percentile	Between Groups	873,476	2	436,738	,621	,539	
	Within Groups	100544,063	143	703,105			
	Total	101417,539	145				
Nucleus_caudatus_right_volume	Between Groups	2,148	2	1,074	6,388	,002	
	Within Groups	24,044	143	,168			
	Total	26,192	145				
Nucleus_caudatus_right_percentile	Between Groups	4493,170	2	2246,585	3,458	,034	
	Within Groups	92915,737	143	649,760			
	Total	97408,906	145				
Nucleus_caudatus_left_volume	Between Groups	2,425	2	1,213	8,908	,000	
	Within Groups	19,467	143	,136			
	Total	21,892	145				
Nucleus_caudatus_left_percentile	Between Groups	4749,248	2	2374,624	4,475	,013	
	Within Groups	75878,823	143	530,621			
	Total	80628,070	145				
Putamen_right_volume	Between Groups	1,784	2	,892	3,734	,026	
	Within Groups	34,156	143	,239			
	Total	35,940	145				
Putamen_right_percentile	Between Groups	533,991	2	266,996	,455	,635	
	Within Groups	83931,352	143	586,933			
	Total	84465,343	145				
Putamen_left_volume	Between Groups	2,126	2	1,063	4,134	,018	
	Within Groups	36,779	143	,257			
	Total	38,905	145				
Putamen_left_percentile	Between Groups	872,145	2	436,073	,715	,491	
	Within Groups	87172,455	143	609,598			
	Total	88044,601	145				
Pallidum_right_volume	Between Groups	,248	2	,124	5,007	,008	
	Within Groups	3,538	143	,025			
	Total	3,786	145				
Pallidum_right_percentile	Between Groups	967,863	2	483,932	,663	,517	
	Within Groups	104454,200	143	730,449			
	Total	105422,063	145				
Pallidum_left_volume	Between Groups	,311	2	,156	6,705	,002	
	Within Groups	3,321	143	,023			
	Total	3,633	145				
Pallidum_left_percentile	Between Groups	737,642	2	368,821	,676	,510	
	Within Groups	77975,432	143	545,283			
	Total	78713,075	145				
Thalamus_right_volume	Between Groups	8,773	2	4,386	7,052	,001	
	Within Groups	88,945	143	,622			
	Total	97,718	145				
Thalamus_right_percentile	Between Groups	2400,590	2	1200,295	1,399	,250	
	Within Groups	122721,634	143	858,193			
	Total	125122,223	145				
Thalamus_left_volume	Between Groups	5,037	2	2,518	2,344	,100	
	Within Groups	153,608	143	1,074			
	Total	158,645	145				
Thalamus_left_percentile	Between Groups	2789,491	2	1394,746	1,702	,186	
	Within Groups	117208,759	143	819,642			
	Total	119998,250	145				
Brainstem_volume	Between Groups	214,383	2	107,192	2,342	,100	
	Within Groups	6545,410	143	45,772			
	Total	6759,793	145				
Brainstem_percentile	Between Groups	1961,826	2	980,913	1,394	,251	
	Within Groups	100613,516	143	703,591			
	Total	102575,342	145				
Mesencephalon_volume	Between Groups	122,822	2	61,411	1,049	,353	
	Within Groups	8373,727	143	58,558			
	Total	8496,549	145				
Mesencephalon_percentile	Between Groups	2040,869	2	1020,435	1,331	,267	
	Within Groups	109643,311	143	766,736			
	Total	111684,181	145				
Pons_volume	Between Groups	130,255	2	65,127	1,309	,273	
	Within Groups	7112,221	143	49,736			
	Total	7242,475	145				
Pons_percentile	Between Groups	1731,199	2	865,600	1,128	,327	
	Within Groups	109731,660	143	767,354			
	Total	111462,859	145				
Cerebellar_grey_matter_volume	Between Groups	1340,699	2	670,349	5,321	,006	
	Within Groups	18015,760	143	125,984			
	Total	19356,459	145				
Cerebellar_grey_matter_percentile	Between Groups	3257,641	2	1628,821	2,447	,090	
	Within Groups	95199,925	143	665,734			
	Total	98457,567	145				
Left_ventricle_volume	Between Groups	128,679	2	64,340	1,693	,188	
	Within Groups	5434,784	143	38,005			
	Total	5563,463	145				
Left_ventricle_percentile	Between Groups	930,191	2	465,096	,542	,583	
	Within Groups	122618,849	143	857,474			
	Total	123549,041	145				
Right_ventricle_volume	Between Groups	58,002	2	29,001	,988	,375	
	Within Groups	4199,128	143	29,365			
	Total	4257,130	145				
Right_ventricle_percentile	Between Groups	358,129	2	179,065	,195	,823	
	Within Groups	130992,106	143	916,029			
	Total	131350,235	145				
Third_ventricle_volume	Between Groups	,159	2	,080	,523	,594	
	Within Groups	21,800	143	,152			
	Total	21,959	145				
Third_ventricle_percentile	Between Groups	2539,715	2	1269,857	1,549	,216	
	Within Groups	117195,518	143	819,549			
	Total	119735,233	145				
Fourth_ventricle_volume	Between Groups	,514	2	,257	1,768	,174	
	Within Groups	20,790	143	,145			
	Total	21,304	145				
Fourth_ventricle_percentile	Between Groups	1544,329	2	772,164	,939	,393	
	Within Groups	117580,337	143	822,240			
	Total	119124,666	145				


Robust Tests of Equality of Means	
	Statistica	df1	df2	Sig.	
Whole_brain_volume	Welch	5,927	2	91,009	,004	
Whole_brain_precentile	Welch	1,478	2	83,977	,234	
Whole_brain_white_substance	Welch	3,441	2	91,477	,036	
Whole_brain_white_substance_percentile	Welch	,117	2	84,262	,890	
Whole_brain_grey_substance	Welch	7,247	2	88,455	,001	
Whole_brain_grey_substance_percentile	Welch	2,446	2	88,936	,092	
Supratentorial_gross_cerebral_cortex_volume	Welch	5,932	2	89,964	,004	
Supratentorial_cerebral_cortex_percentile	Welch	1,729	2	89,427	,183	
Frontal_right_volume	Welch	6,037	2	90,799	,003	
Frontal_right_percentile	Welch	2,430	2	89,585	,094	
Frontal_left_volume	Welch	4,170	2	88,718	,019	
Frontal_left_percentile	Welch	1,247	2	88,874	,292	
Parietal_right_volume	Welch	5,605	2	90,699	,005	
Parietal_right_percentile	Welch	1,915	2	89,304	,153	
Parietal_left_volume	Welch	6,459	2	91,330	,002	
Parietal_left_percentile	Welch	2,881	2	90,360	,061	
Precuneus_right_volume	Welch	4,870	2	91,647	,010	
Precuneus_right_percentile	Welch	,243	2	90,939	,785	
Precuneus_left_volume	Welch	3,251	2	92,355	,043	
Precuneus_left_percentile	Welch	1,036	2	85,896	,359	
Occipital_right_volume	Welch	4,620	2	81,607	,013	
Occipital_right_percentlile	Welch	,287	2	91,650	,751	
Occipital_left_volume	Welch	4,569	2	95,073	,013	
Occipital_left_percentile	Welch	,188	2	90,098	,829	
Temporal_right_volume	Welch	5,416	2	90,338	,006	
Temporal_right_percentile	Welch	,304	2	91,345	,738	
Temporal_left_volume	Welch	3,774	2	94,183	,027	
Temporal_left_percentlile	Welch	1,410	2	92,480	,249	
Mesiotemporal_right_volume	Welch	1,382	2	91,316	,256	
Mesiotemporal_right_percentile	Welch	,521	2	89,863	,596	
Mesiotemproal_left_volume	Welch	1,525	2	91,632	,223	
Mesiotemporal_left_percentile	Welch	,550	2	90,269	,579	
Hippocampus_right_volume	Welch	3,043	2	90,322	,053	
Hippocampus_right_percentile	Welch	,080	2	90,845	,924	
Hippocampus_left_volume	Welch	1,579	2	72,049	,213	
Hippocampus_left_percentile	Welch	,004	2	91,362	,996	
Gyrus_parahippocampalis_right_volume	Welch	2,754	2	92,347	,069	
Gyrus_parahippocampalis_right_percentile	Welch	1,041	2	93,355	,357	
Gyrus_parahippocampalis_left_volume	Welch	6,631	2	92,822	,002	
Gyrus_parahippocampalis_left_percentile	Welch	2,962	2	89,917	,057	
Regio_entorhinalis_right_volume	Welch	3,013	2	93,745	,054	
Regio_entorhinalis_right_percentile	Welch	,583	2	93,377	,560	
Regio_entorhinalis_left_volume	Welch	1,470	2	89,245	,235	
Regio_entorhinalis_left_percentile	Welch	,623	2	90,381	,538	
Nucleus_caudatus_right_volume	Welch	6,672	2	91,225	,002	
Nucleus_caudatus_right_percentile	Welch	3,497	2	90,282	,034	
Nucleus_caudatus_left_volume	Welch	9,119	2	89,715	,000	
Nucleus_caudatus_left_percentile	Welch	4,416	2	89,067	,015	
Putamen_right_volume	Welch	3,451	2	88,894	,036	
Putamen_right_percentile	Welch	,468	2	87,479	,628	
Putamen_left_volume	Welch	3,899	2	89,762	,024	
Putamen_left_percentile	Welch	,699	2	90,663	,500	
Pallidum_right_volume	Welch	5,692	2	92,442	,005	
Pallidum_right_percentile	Welch	,679	2	89,554	,510	
Pallidum_left_volume	Welch	6,800	2	91,475	,002	
Pallidum_left_percentile	Welch	,662	2	89,769	,518	
Thalamus_right_volume	Welch	6,684	2	89,451	,002	
Thalamus_right_percentile	Welch	1,400	2	90,704	,252	
Thalamus_left_volume	Welch	3,282	2	88,451	,042	
Thalamus_left_percentile	Welch	1,607	2	89,949	,206	
Brainstem_volume	Welch	4,630	2	90,561	,012	
Brainstem_percentile	Welch	1,397	2	89,708	,253	
Mesencephalon_volume	Welch	3,006	2	88,306	,055	
Mesencephalon_percentile	Welch	1,363	2	90,745	,261	
Pons_volume	Welch	3,399	2	90,412	,038	
Pons_percentile	Welch	1,200	2	90,826	,306	
Cerebellar_grey_matter_volume	Welch	5,174	2	89,875	,007	
Cerebellar_grey_matter_percentile	Welch	2,206	2	88,351	,116	
Left_ventricle_volume	Welch	1,737	2	95,248	,182	
Left_ventricle_percentile	Welch	,511	2	92,680	,602	
Right_ventricle_volume	Welch	1,156	2	95,292	,319	
Right_ventricle_percentile	Welch	,189	2	91,299	,828	
Third_ventricle_volume	Welch	,497	2	92,213	,610	
Third_ventricle_percentile	Welch	1,596	2	91,656	,208	
Fourth_ventricle_volume	Welch	1,922	2	91,963	,152	
Fourth_ventricle_percentile	Welch	,962	2	88,946	,386	

a. Asymptotically F distributed.	


Post Hoc Tests


Multiple Comparisons	
Bonferroni  	
Dependent Variable	(I) COVID_severity	(J) COVID_severity	Mean Difference (I-J)	Std. Error	Sig.	95% Confidence Interval	
						Lower Bound	Upper Bound	
Whole_brain_volume	Control Group	Asymptomatic non-hospitalised COVID-19	-34,01145	24,42166	,498	-93,1722	25,1493	
		Severe hospitalised COVID-19	57,16653	26,31467	,094	-6,5800	120,9130	
	Asymptomatic non-hospitalised COVID-19	Control Group	34,01145	24,42166	,498	-25,1493	93,1722	
		Severe hospitalised COVID-19	91,17798*	26,83900	,003	26,1613	156,1946	
	Severe hospitalised COVID-19	Control Group	-57,16653	26,31467	,094	-120,9130	6,5800	
		Asymptomatic non-hospitalised COVID-19	-91,17798*	26,83900	,003	-156,1946	-26,1613	
Whole_brain_precentile	Control Group	Asymptomatic non-hospitalised COVID-19	1,53207	4,08335	1,000	-8,3597	11,4239	
		Severe hospitalised COVID-19	7,98260	4,39987	,215	-2,6760	18,6412	
	Asymptomatic non-hospitalised COVID-19	Control Group	-1,53207	4,08335	1,000	-11,4239	8,3597	
		Severe hospitalised COVID-19	6,45053	4,48754	,458	-4,4204	17,3215	
	Severe hospitalised COVID-19	Control Group	-7,98260	4,39987	,215	-18,6412	2,6760	
		Asymptomatic non-hospitalised COVID-19	-6,45053	4,48754	,458	-17,3215	4,4204	
Whole_brain_white_substance	Control Group	Asymptomatic non-hospitalised COVID-19	-17,54538	12,62754	,501	-48,1352	13,0445	
		Severe hospitalised COVID-19	18,87106	13,60635	,503	-14,0899	51,8320	
	Asymptomatic non-hospitalised COVID-19	Control Group	17,54538	12,62754	,501	-13,0445	48,1352	
		Severe hospitalised COVID-19	36,41644*	13,87746	,029	2,7987	70,0342	
	Severe hospitalised COVID-19	Control Group	-18,87106	13,60635	,503	-51,8320	14,0899	
		Asymptomatic non-hospitalised COVID-19	-36,41644*	13,87746	,029	-70,0342	-2,7987	
Whole_brain_white_substance_percentile	Control Group	Asymptomatic non-hospitalised COVID-19	-,02171	3,55050	1,000	-8,6227	8,5793	
		Severe hospitalised COVID-19	1,93168	3,82571	1,000	-7,3360	11,1994	
	Asymptomatic non-hospitalised COVID-19	Control Group	,02171	3,55050	1,000	-8,5793	8,6227	
		Severe hospitalised COVID-19	1,95339	3,90194	1,000	-7,4989	11,4057	
	Severe hospitalised COVID-19	Control Group	-1,93168	3,82571	1,000	-11,1994	7,3360	
		Asymptomatic non-hospitalised COVID-19	-1,95339	3,90194	1,000	-11,4057	7,4989	
Whole_brain_grey_substance	Control Group	Asymptomatic non-hospitalised COVID-19	-17,04559	13,90515	,667	-50,7304	16,6392	
		Severe hospitalised COVID-19	42,14038*	14,98298	,017	5,8446	78,4362	
	Asymptomatic non-hospitalised COVID-19	Control Group	17,04559	13,90515	,667	-16,6392	50,7304	
		Severe hospitalised COVID-19	59,18597*	15,28152	,000	22,1669	96,2050	
	Severe hospitalised COVID-19	Control Group	-42,14038*	14,98298	,017	-78,4362	-5,8446	
		Asymptomatic non-hospitalised COVID-19	-59,18597*	15,28152	,000	-96,2050	-22,1669	
Whole_brain_grey_substance_percentile	Control Group	Asymptomatic non-hospitalised COVID-19	2,95515	4,97847	1,000	-9,1050	15,0153	
		Severe hospitalised COVID-19	11,77596	5,36436	,089	-1,2190	24,7710	
	Asymptomatic non-hospitalised COVID-19	Control Group	-2,95515	4,97847	1,000	-15,0153	9,1050	
		Severe hospitalised COVID-19	8,82081	5,47125	,327	-4,4331	22,0748	
	Severe hospitalised COVID-19	Control Group	-11,77596	5,36436	,089	-24,7710	1,2190	
		Asymptomatic non-hospitalised COVID-19	-8,82081	5,47125	,327	-22,0748	4,4331	
Supratentorial_gross_cerebral_cortex_volume	Control Group	Asymptomatic non-hospitalised COVID-19	-7,49237	9,85207	1,000	-31,3587	16,3740	
		Severe hospitalised COVID-19	27,69753*	10,61574	,030	1,9812	53,4138	
	Asymptomatic non-hospitalised COVID-19	Control Group	7,49237	9,85207	1,000	-16,3740	31,3587	
		Severe hospitalised COVID-19	35,18989*	10,82726	,004	8,9612	61,4186	
	Severe hospitalised COVID-19	Control Group	-27,69753*	10,61574	,030	-53,4138	-1,9812	
		Asymptomatic non-hospitalised COVID-19	-35,18989*	10,82726	,004	-61,4186	-8,9612	
Supratentorial_cerebral_cortex_percentile	Control Group	Asymptomatic non-hospitalised COVID-19	6,14580	5,10637	,692	-6,2242	18,5158	
		Severe hospitalised COVID-19	10,06465	5,50218	,208	-3,2642	23,3935	
	Asymptomatic non-hospitalised COVID-19	Control Group	-6,14580	5,10637	,692	-18,5158	6,2242	
		Severe hospitalised COVID-19	3,91885	5,61181	1,000	-9,6756	17,5133	
	Severe hospitalised COVID-19	Control Group	-10,06465	5,50218	,208	-23,3935	3,2642	
		Asymptomatic non-hospitalised COVID-19	-3,91885	5,61181	1,000	-17,5133	9,6756	
Frontal_right_volume	Control Group	Asymptomatic non-hospitalised COVID-19	-1,53694	1,97955	1,000	-6,3323	3,2585	
		Severe hospitalised COVID-19	5,61268*	2,13299	,028	,4456	10,7798	
	Asymptomatic non-hospitalised COVID-19	Control Group	1,53694	1,97955	1,000	-3,2585	6,3323	
		Severe hospitalised COVID-19	7,14962*	2,17549	,004	1,8796	12,4197	
	Severe hospitalised COVID-19	Control Group	-5,61268*	2,13299	,028	-10,7798	-,4456	
		Asymptomatic non-hospitalised COVID-19	-7,14962*	2,17549	,004	-12,4197	-1,8796	
Frontal_right_percentile	Control Group	Asymptomatic non-hospitalised COVID-19	8,46597	5,32101	,341	-4,4240	21,3559	
		Severe hospitalised COVID-19	11,87125	5,73346	,121	-2,0179	25,7604	
	Asymptomatic non-hospitalised COVID-19	Control Group	-8,46597	5,32101	,341	-21,3559	4,4240	
		Severe hospitalised COVID-19	3,40528	5,84770	1,000	-10,7606	17,5712	
	Severe hospitalised COVID-19	Control Group	-11,87125	5,73346	,121	-25,7604	2,0179	
		Asymptomatic non-hospitalised COVID-19	-3,40528	5,84770	1,000	-17,5712	10,7606	
Frontal_left_volume	Control Group	Asymptomatic non-hospitalised COVID-19	-,70389	2,12938	1,000	-5,8622	4,4545	
		Severe hospitalised COVID-19	5,06067	2,29443	,087	-,4975	10,6189	
	Asymptomatic non-hospitalised COVID-19	Control Group	,70389	2,12938	1,000	-4,4545	5,8622	
		Severe hospitalised COVID-19	5,76456*	2,34015	,045	,0956	11,4335	
	Severe hospitalised COVID-19	Control Group	-5,06067	2,29443	,087	-10,6189	,4975	
		Asymptomatic non-hospitalised COVID-19	-5,76456*	2,34015	,045	-11,4335	-,0956	
Frontal_left_percentile	Control Group	Asymptomatic non-hospitalised COVID-19	4,92675	5,19743	1,000	-7,6639	17,5174	
		Severe hospitalised COVID-19	8,69945	5,60030	,368	-4,8671	22,2660	
	Asymptomatic non-hospitalised COVID-19	Control Group	-4,92675	5,19743	1,000	-17,5174	7,6639	
		Severe hospitalised COVID-19	3,77270	5,71189	1,000	-10,0642	17,6096	
	Severe hospitalised COVID-19	Control Group	-8,69945	5,60030	,368	-22,2660	4,8671	
		Asymptomatic non-hospitalised COVID-19	-3,77270	5,71189	1,000	-17,6096	10,0642	
Parietal_right_volume	Control Group	Asymptomatic non-hospitalised COVID-19	-,43459	1,04288	1,000	-2,9609	2,0917	
		Severe hospitalised COVID-19	3,05696*	1,12371	,022	,3348	5,7791	
	Asymptomatic non-hospitalised COVID-19	Control Group	,43459	1,04288	1,000	-2,0917	2,9609	
		Severe hospitalised COVID-19	3,49155*	1,14610	,008	,7152	6,2680	
	Severe hospitalised COVID-19	Control Group	-3,05696*	1,12371	,022	-5,7791	-,3348	
		Asymptomatic non-hospitalised COVID-19	-3,49155*	1,14610	,008	-6,2680	-,7152	
Parietal_right_percentile	Control Group	Asymptomatic non-hospitalised COVID-19	7,89762	4,24194	,194	-2,3784	18,1736	
		Severe hospitalised COVID-19	6,01557	4,57075	,571	-5,0569	17,0881	
	Asymptomatic non-hospitalised COVID-19	Control Group	-7,89762	4,24194	,194	-18,1736	2,3784	
		Severe hospitalised COVID-19	-1,88205	4,66182	1,000	-13,1752	9,4111	
	Severe hospitalised COVID-19	Control Group	-6,01557	4,57075	,571	-17,0881	5,0569	
		Asymptomatic non-hospitalised COVID-19	1,88205	4,66182	1,000	-9,4111	13,1752	
Parietal_left_volume	Control Group	Asymptomatic non-hospitalised COVID-19	-,34387	1,08096	1,000	-2,9625	2,2747	
		Severe hospitalised COVID-19	3,43878*	1,16475	,011	,6172	6,2603	
	Asymptomatic non-hospitalised COVID-19	Control Group	,34387	1,08096	1,000	-2,2747	2,9625	
		Severe hospitalised COVID-19	3,78265*	1,18795	,005	,9049	6,6604	
	Severe hospitalised COVID-19	Control Group	-3,43878*	1,16475	,011	-6,2603	-,6172	
		Asymptomatic non-hospitalised COVID-19	-3,78265*	1,18795	,005	-6,6604	-,9049	
Parietal_left_percentile	Control Group	Asymptomatic non-hospitalised COVID-19	9,52199	4,77421	,144	-2,0434	21,0874	
		Severe hospitalised COVID-19	10,99469	5,14428	,103	-1,4672	23,4566	
	Asymptomatic non-hospitalised COVID-19	Control Group	-9,52199	4,77421	,144	-21,0874	2,0434	
		Severe hospitalised COVID-19	1,47270	5,24678	1,000	-11,2375	14,1829	
	Severe hospitalised COVID-19	Control Group	-10,99469	5,14428	,103	-23,4566	1,4672	
		Asymptomatic non-hospitalised COVID-19	-1,47270	5,24678	1,000	-14,1829	11,2375	
Precuneus_right_volume	Control Group	Asymptomatic non-hospitalised COVID-19	-,22703	,28602	1,000	-,9199	,4658	
		Severe hospitalised COVID-19	,69725	,30819	,076	-,0493	1,4438	
	Asymptomatic non-hospitalised COVID-19	Control Group	,22703	,28602	1,000	-,4658	,9199	
		Severe hospitalised COVID-19	,92428*	,31433	,011	,1628	1,6857	
	Severe hospitalised COVID-19	Control Group	-,69725	,30819	,076	-1,4438	,0493	
		Asymptomatic non-hospitalised COVID-19	-,92428*	,31433	,011	-1,6857	-,1628	
Precuneus_right_percentile	Control Group	Asymptomatic non-hospitalised COVID-19	2,10193	5,40249	1,000	-10,9854	15,1893	
		Severe hospitalised COVID-19	4,13933	5,82126	1,000	-9,9625	18,2412	
	Asymptomatic non-hospitalised COVID-19	Control Group	-2,10193	5,40249	1,000	-15,1893	10,9854	
		Severe hospitalised COVID-19	2,03741	5,93725	1,000	-12,3454	16,4202	
	Severe hospitalised COVID-19	Control Group	-4,13933	5,82126	1,000	-18,2412	9,9625	
		Asymptomatic non-hospitalised COVID-19	-2,03741	5,93725	1,000	-16,4202	12,3454	
Precuneus_left_volume	Control Group	Asymptomatic non-hospitalised COVID-19	-,25167	,36033	1,000	-1,1246	,6212	
		Severe hospitalised COVID-19	,67201	,38826	,257	-,2685	1,6126	
	Asymptomatic non-hospitalised COVID-19	Control Group	,25167	,36033	1,000	-,6212	1,1246	
		Severe hospitalised COVID-19	,92368	,39600	,063	-,0356	1,8830	
	Severe hospitalised COVID-19	Control Group	-,67201	,38826	,257	-1,6126	,2685	
		Asymptomatic non-hospitalised COVID-19	-,92368	,39600	,063	-1,8830	,0356	
Precuneus_left_percentile	Control Group	Asymptomatic non-hospitalised COVID-19	5,36310	5,07455	,877	-6,9298	17,6560	
		Severe hospitalised COVID-19	7,20412	5,46789	,569	-6,0417	20,4499	
	Asymptomatic non-hospitalised COVID-19	Control Group	-5,36310	5,07455	,877	-17,6560	6,9298	
		Severe hospitalised COVID-19	1,84103	5,57684	1,000	-11,6687	15,3508	
	Severe hospitalised COVID-19	Control Group	-7,20412	5,46789	,569	-20,4499	6,0417	
		Asymptomatic non-hospitalised COVID-19	-1,84103	5,57684	1,000	-15,3508	11,6687	
Occipital_right_volume	Control Group	Asymptomatic non-hospitalised COVID-19	-1,74737	1,22066	,463	-4,7044	1,2096	
		Severe hospitalised COVID-19	2,00014	1,31527	,392	-1,1861	5,1863	
	Asymptomatic non-hospitalised COVID-19	Control Group	1,74737	1,22066	,463	-1,2096	4,7044	
		Severe hospitalised COVID-19	3,74751*	1,34148	,018	,4978	6,9972	
	Severe hospitalised COVID-19	Control Group	-2,00014	1,31527	,392	-5,1863	1,1861	
		Asymptomatic non-hospitalised COVID-19	-3,74751*	1,34148	,018	-6,9972	-,4978	
Occipital_right_percentlile	Control Group	Asymptomatic non-hospitalised COVID-19	1,77826	4,54358	1,000	-9,2284	12,7849	
		Severe hospitalised COVID-19	3,64281	4,89576	1,000	-8,2170	15,5027	
	Asymptomatic non-hospitalised COVID-19	Control Group	-1,77826	4,54358	1,000	-12,7849	9,2284	
		Severe hospitalised COVID-19	1,86456	4,99331	1,000	-10,2316	13,9607	
	Severe hospitalised COVID-19	Control Group	-3,64281	4,89576	1,000	-15,5027	8,2170	
		Asymptomatic non-hospitalised COVID-19	-1,86456	4,99331	1,000	-13,9607	10,2316	
Occipital_left_volume	Control Group	Asymptomatic non-hospitalised COVID-19	,04195	1,36178	1,000	-3,2569	3,3408	
		Severe hospitalised COVID-19	3,18855	1,46733	,094	-,3660	6,7431	
	Asymptomatic non-hospitalised COVID-19	Control Group	-,04195	1,36178	1,000	-3,3408	3,2569	
		Severe hospitalised COVID-19	3,14661	1,49657	,112	-,4788	6,7720	
	Severe hospitalised COVID-19	Control Group	-3,18855	1,46733	,094	-6,7431	,3660	
		Asymptomatic non-hospitalised COVID-19	-3,14661	1,49657	,112	-6,7720	,4788	
Occipital_left_percentile	Control Group	Asymptomatic non-hospitalised COVID-19	2,45385	5,43296	1,000	-10,7073	15,6150	
		Severe hospitalised COVID-19	3,30046	5,85409	1,000	-10,8809	17,4818	
	Asymptomatic non-hospitalised COVID-19	Control Group	-2,45385	5,43296	1,000	-15,6150	10,7073	
		Severe hospitalised COVID-19	,84661	5,97073	1,000	-13,6173	15,3105	
	Severe hospitalised COVID-19	Control Group	-3,30046	5,85409	1,000	-17,4818	10,8809	
		Asymptomatic non-hospitalised COVID-19	-,84661	5,97073	1,000	-15,3105	13,6173	
Temporal_right_volume	Control Group	Asymptomatic non-hospitalised COVID-19	-2,23690	1,53998	,446	-5,9675	1,4937	
		Severe hospitalised COVID-19	3,35284	1,65935	,136	-,6669	7,3726	
	Asymptomatic non-hospitalised COVID-19	Control Group	2,23690	1,53998	,446	-1,4937	5,9675	
		Severe hospitalised COVID-19	5,58974*	1,69242	,004	1,4899	9,6896	
	Severe hospitalised COVID-19	Control Group	-3,35284	1,65935	,136	-7,3726	,6669	
		Asymptomatic non-hospitalised COVID-19	-5,58974*	1,69242	,004	-9,6896	-1,4899	
Temporal_right_percentile	Control Group	Asymptomatic non-hospitalised COVID-19	-1,53064	5,31068	1,000	-14,3956	11,3343	
		Severe hospitalised COVID-19	2,90481	5,72233	1,000	-10,9574	16,7670	
	Asymptomatic non-hospitalised COVID-19	Control Group	1,53064	5,31068	1,000	-11,3343	14,3956	
		Severe hospitalised COVID-19	4,43544	5,83635	1,000	-9,7029	18,5738	
	Severe hospitalised COVID-19	Control Group	-2,90481	5,72233	1,000	-16,7670	10,9574	
		Asymptomatic non-hospitalised COVID-19	-4,43544	5,83635	1,000	-18,5738	9,7029	
Temporal_left_volume	Control Group	Asymptomatic non-hospitalised COVID-19	-1,73375	1,60742	,848	-5,6277	2,1602	
		Severe hospitalised COVID-19	2,64634	1,73202	,386	-1,5494	6,8421	
	Asymptomatic non-hospitalised COVID-19	Control Group	1,73375	1,60742	,848	-2,1602	5,6277	
		Severe hospitalised COVID-19	4,38009*	1,76653	,043	,1007	8,6595	
	Severe hospitalised COVID-19	Control Group	-2,64634	1,73202	,386	-6,8421	1,5494	
		Asymptomatic non-hospitalised COVID-19	-4,38009*	1,76653	,043	-8,6595	-,1007	
Temporal_left_percentlile	Control Group	Asymptomatic non-hospitalised COVID-19	-2,61225	5,45332	1,000	-15,8228	10,5983	
		Severe hospitalised COVID-19	6,70962	5,87603	,766	-7,5249	20,9441	
	Asymptomatic non-hospitalised COVID-19	Control Group	2,61225	5,45332	1,000	-10,5983	15,8228	
		Severe hospitalised COVID-19	9,32187	5,99311	,366	-5,1963	23,8400	
	Severe hospitalised COVID-19	Control Group	-6,70962	5,87603	,766	-20,9441	7,5249	
		Asymptomatic non-hospitalised COVID-19	-9,32187	5,99311	,366	-23,8400	5,1963	
Mesiotemporal_right_volume	Control Group	Asymptomatic non-hospitalised COVID-19	-2,42717	2,08205	,737	-7,4709	2,6165	
		Severe hospitalised COVID-19	1,19048	2,24344	1,000	-4,2442	6,6251	
	Asymptomatic non-hospitalised COVID-19	Control Group	2,42717	2,08205	,737	-2,6165	7,4709	
		Severe hospitalised COVID-19	3,61765	2,28814	,348	-1,9253	9,1606	
	Severe hospitalised COVID-19	Control Group	-1,19048	2,24344	1,000	-6,6251	4,2442	
		Asymptomatic non-hospitalised COVID-19	-3,61765	2,28814	,348	-9,1606	1,9253	
Mesiotemporal_right_percentile	Control Group	Asymptomatic non-hospitalised COVID-19	-4,58708	6,06188	1,000	-19,2718	10,0976	
		Severe hospitalised COVID-19	1,53796	6,53176	1,000	-14,2850	17,3609	
	Asymptomatic non-hospitalised COVID-19	Control Group	4,58708	6,06188	1,000	-10,0976	19,2718	
		Severe hospitalised COVID-19	6,12504	6,66191	1,000	-10,0132	22,2633	
	Severe hospitalised COVID-19	Control Group	-1,53796	6,53176	1,000	-17,3609	14,2850	
		Asymptomatic non-hospitalised COVID-19	-6,12504	6,66191	1,000	-22,2633	10,0132	
Mesiotemproal_left_volume	Control Group	Asymptomatic non-hospitalised COVID-19	-2,25840	1,90768	,715	-6,8797	2,3629	
		Severe hospitalised COVID-19	1,21007	2,05555	1,000	-3,7694	6,1896	
	Asymptomatic non-hospitalised COVID-19	Control Group	2,25840	1,90768	,715	-2,3629	6,8797	
		Severe hospitalised COVID-19	3,46848	2,09651	,301	-1,6103	8,5472	
	Severe hospitalised COVID-19	Control Group	-1,21007	2,05555	1,000	-6,1896	3,7694	
		Asymptomatic non-hospitalised COVID-19	-3,46848	2,09651	,301	-8,5472	1,6103	
Mesiotemporal_left_percentile	Control Group	Asymptomatic non-hospitalised COVID-19	-4,40641	6,03790	1,000	-19,0330	10,2202	
		Severe hospitalised COVID-19	1,88997	6,50591	1,000	-13,8704	17,6504	
	Asymptomatic non-hospitalised COVID-19	Control Group	4,40641	6,03790	1,000	-10,2202	19,0330	
		Severe hospitalised COVID-19	6,29638	6,63555	1,000	-9,7780	22,3708	
	Severe hospitalised COVID-19	Control Group	-1,88997	6,50591	1,000	-17,6504	13,8704	
		Asymptomatic non-hospitalised COVID-19	-6,29638	6,63555	1,000	-22,3708	9,7780	
Hippocampus_right_volume	Control Group	Asymptomatic non-hospitalised COVID-19	,66849	,70775	1,000	-1,0460	2,3830	
		Severe hospitalised COVID-19	,88553	,76261	,743	-,9619	2,7329	
	Asymptomatic non-hospitalised COVID-19	Control Group	-,66849	,70775	1,000	-2,3830	1,0460	
		Severe hospitalised COVID-19	,21704	,77781	1,000	-1,6672	2,1013	
	Severe hospitalised COVID-19	Control Group	-,88553	,76261	,743	-2,7329	,9619	
		Asymptomatic non-hospitalised COVID-19	-,21704	,77781	1,000	-2,1013	1,6672	
Hippocampus_right_percentile	Control Group	Asymptomatic non-hospitalised COVID-19	-1,28620	5,29152	1,000	-14,1048	11,5323	
		Severe hospitalised COVID-19	1,03159	5,70169	1,000	-12,7806	14,8438	
	Asymptomatic non-hospitalised COVID-19	Control Group	1,28620	5,29152	1,000	-11,5323	14,1048	
		Severe hospitalised COVID-19	2,31780	5,81529	1,000	-11,7696	16,4052	
	Severe hospitalised COVID-19	Control Group	-1,03159	5,70169	1,000	-14,8438	12,7806	
		Asymptomatic non-hospitalised COVID-19	-2,31780	5,81529	1,000	-16,4052	11,7696	
Hippocampus_left_volume	Control Group	Asymptomatic non-hospitalised COVID-19	,67423	1,35605	1,000	-2,6108	3,9592	
		Severe hospitalised COVID-19	1,46593	1,46117	,952	-2,0737	5,0056	
	Asymptomatic non-hospitalised COVID-19	Control Group	-,67423	1,35605	1,000	-3,9592	2,6108	
		Severe hospitalised COVID-19	,79170	1,49028	1,000	-2,8185	4,4019	
	Severe hospitalised COVID-19	Control Group	-1,46593	1,46117	,952	-5,0056	2,0737	
		Asymptomatic non-hospitalised COVID-19	-,79170	1,49028	1,000	-4,4019	2,8185	
Hippocampus_left_percentile	Control Group	Asymptomatic non-hospitalised COVID-19	-,32395	5,09809	1,000	-12,6739	12,0260	
		Severe hospitalised COVID-19	,13352	5,49326	1,000	-13,1737	13,4408	
	Asymptomatic non-hospitalised COVID-19	Control Group	,32395	5,09809	1,000	-12,0260	12,6739	
		Severe hospitalised COVID-19	,45747	5,60271	1,000	-13,1149	14,0299	
	Severe hospitalised COVID-19	Control Group	-,13352	5,49326	1,000	-13,4408	13,1737	
		Asymptomatic non-hospitalised COVID-19	-,45747	5,60271	1,000	-14,0299	13,1149	
Gyrus_parahippocampalis_right_volume	Control Group	Asymptomatic non-hospitalised COVID-19	-,12679	,07503	,280	-,3085	,0550	
		Severe hospitalised COVID-19	,06296	,08084	1,000	-,1329	,2588	
	Asymptomatic non-hospitalised COVID-19	Control Group	,12679	,07503	,280	-,0550	,3085	
		Severe hospitalised COVID-19	,18974	,08245	,068	-,0100	,3895	
	Severe hospitalised COVID-19	Control Group	-,06296	,08084	1,000	-,2588	,1329	
		Asymptomatic non-hospitalised COVID-19	-,18974	,08245	,068	-,3895	,0100	
Gyrus_parahippocampalis_right_percentile	Control Group	Asymptomatic non-hospitalised COVID-19	-7,05021	5,27350	,550	-19,8251	5,7247	
		Severe hospitalised COVID-19	-6,51145	5,68227	,761	-20,2766	7,2537	
	Asymptomatic non-hospitalised COVID-19	Control Group	7,05021	5,27350	,550	-5,7247	19,8251	
		Severe hospitalised COVID-19	,53876	5,79549	1,000	-13,5006	14,5782	
	Severe hospitalised COVID-19	Control Group	6,51145	5,68227	,761	-7,2537	20,2766	
		Asymptomatic non-hospitalised COVID-19	-,53876	5,79549	1,000	-14,5782	13,5006	
Gyrus_parahippocampalis_left_volume	Control Group	Asymptomatic non-hospitalised COVID-19	-,14009	,06447	,094	-,2963	,0161	
		Severe hospitalised COVID-19	,11149	,06946	,332	-,0568	,2798	
	Asymptomatic non-hospitalised COVID-19	Control Group	,14009	,06447	,094	-,0161	,2963	
		Severe hospitalised COVID-19	,25158*	,07085	,002	,0800	,4232	
	Severe hospitalised COVID-19	Control Group	-,11149	,06946	,332	-,2798	,0568	
		Asymptomatic non-hospitalised COVID-19	-,25158*	,07085	,002	-,4232	-,0800	
Gyrus_parahippocampalis_left_percentile	Control Group	Asymptomatic non-hospitalised COVID-19	-10,85266	5,00547	,095	-22,9783	1,2729	
		Severe hospitalised COVID-19	-,94286	5,39346	1,000	-14,0084	12,1226	
	Asymptomatic non-hospitalised COVID-19	Control Group	10,85266	5,00547	,095	-1,2729	22,9783	
		Severe hospitalised COVID-19	9,90980	5,50093	,221	-3,4160	23,2356	
	Severe hospitalised COVID-19	Control Group	,94286	5,39346	1,000	-12,1226	14,0084	
		Asymptomatic non-hospitalised COVID-19	-9,90980	5,50093	,221	-23,2356	3,4160	
Regio_entorhinalis_right_volume	Control Group	Asymptomatic non-hospitalised COVID-19	-,09072	,05783	,357	-,2308	,0494	
		Severe hospitalised COVID-19	,05664	,06231	1,000	-,0943	,2076	
	Asymptomatic non-hospitalised COVID-19	Control Group	,09072	,05783	,357	-,0494	,2308	
		Severe hospitalised COVID-19	,14736	,06355	,065	-,0066	,3013	
	Severe hospitalised COVID-19	Control Group	-,05664	,06231	1,000	-,2076	,0943	
		Asymptomatic non-hospitalised COVID-19	-,14736	,06355	,065	-,3013	,0066	
Regio_entorhinalis_right_percentile	Control Group	Asymptomatic non-hospitalised COVID-19	-4,90630	4,52643	,841	-15,8714	6,0588	
		Severe hospitalised COVID-19	-1,74597	4,87729	1,000	-13,5611	10,0691	
	Asymptomatic non-hospitalised COVID-19	Control Group	4,90630	4,52643	,841	-6,0588	15,8714	
		Severe hospitalised COVID-19	3,16033	4,97447	1,000	-8,8902	15,2108	
	Severe hospitalised COVID-19	Control Group	1,74597	4,87729	1,000	-10,0691	13,5611	
		Asymptomatic non-hospitalised COVID-19	-3,16033	4,97447	1,000	-15,2108	8,8902	
Regio_entorhinalis_left_volume	Control Group	Asymptomatic non-hospitalised COVID-19	-,06408	,05714	,792	-,2025	,0743	
		Severe hospitalised COVID-19	,04588	,06157	1,000	-,1033	,1950	
	Asymptomatic non-hospitalised COVID-19	Control Group	,06408	,05714	,792	-,0743	,2025	
		Severe hospitalised COVID-19	,10995	,06279	,246	-,0422	,2621	
	Severe hospitalised COVID-19	Control Group	-,04588	,06157	1,000	-,1950	,1033	
		Asymptomatic non-hospitalised COVID-19	-,10995	,06279	,246	-,2621	,0422	
Regio_entorhinalis_left_percentile	Control Group	Asymptomatic non-hospitalised COVID-19	-4,87787	5,13243	1,000	-17,3110	7,5553	
		Severe hospitalised COVID-19	,56300	5,53026	1,000	-12,8339	13,9599	
	Asymptomatic non-hospitalised COVID-19	Control Group	4,87787	5,13243	1,000	-7,5553	17,3110	
		Severe hospitalised COVID-19	5,44087	5,64045	1,000	-8,2230	19,1047	
	Severe hospitalised COVID-19	Control Group	-,56300	5,53026	1,000	-13,9599	12,8339	
		Asymptomatic non-hospitalised COVID-19	-5,44087	5,64045	1,000	-19,1047	8,2230	
Nucleus_caudatus_right_volume	Control Group	Asymptomatic non-hospitalised COVID-19	-,18824	,07937	,057	-,3805	,0040	
		Severe hospitalised COVID-19	,11538	,08552	,538	-,0918	,3226	
	Asymptomatic non-hospitalised COVID-19	Control Group	,18824	,07937	,057	-,0040	,3805	
		Severe hospitalised COVID-19	,30362*	,08722	,002	,0923	,5149	
	Severe hospitalised COVID-19	Control Group	-,11538	,08552	,538	-,3226	,0918	
		Asymptomatic non-hospitalised COVID-19	-,30362*	,08722	,002	-,5149	-,0923	
Nucleus_caudatus_right_percentile	Control Group	Asymptomatic non-hospitalised COVID-19	-11,69377	4,93389	,057	-23,6460	,2584	
		Severe hospitalised COVID-19	-,14203	5,31633	1,000	-13,0207	12,7366	
	Asymptomatic non-hospitalised COVID-19	Control Group	11,69377	4,93389	,057	-,2584	23,6460	
		Severe hospitalised COVID-19	11,55173	5,42226	,105	-1,5835	24,6870	
	Severe hospitalised COVID-19	Control Group	,14203	5,31633	1,000	-12,7366	13,0207	
		Asymptomatic non-hospitalised COVID-19	-11,55173	5,42226	,105	-24,6870	1,5835	
Nucleus_caudatus_left_volume	Control Group	Asymptomatic non-hospitalised COVID-19	-,22223*	,07142	,007	-,3952	-,0492	
		Severe hospitalised COVID-19	,09089	,07695	,719	-,0955	,2773	
	Asymptomatic non-hospitalised COVID-19	Control Group	,22223*	,07142	,007	,0492	,3952	
		Severe hospitalised COVID-19	,31312*	,07848	,000	,1230	,5032	
	Severe hospitalised COVID-19	Control Group	-,09089	,07695	,719	-,2773	,0955	
		Asymptomatic non-hospitalised COVID-19	-,31312*	,07848	,000	-,5032	-,1230	
Nucleus_caudatus_left_percentile	Control Group	Asymptomatic non-hospitalised COVID-19	-12,13999*	4,45867	,022	-22,9410	-1,3390	
		Severe hospitalised COVID-19	-,44496	4,80427	1,000	-12,0832	11,1932	
	Asymptomatic non-hospitalised COVID-19	Control Group	12,13999*	4,45867	,022	1,3390	22,9410	
		Severe hospitalised COVID-19	11,69502	4,90000	,055	-,1751	23,5651	
	Severe hospitalised COVID-19	Control Group	,44496	4,80427	1,000	-11,1932	12,0832	
		Asymptomatic non-hospitalised COVID-19	-11,69502	4,90000	,055	-23,5651	,1751	
Putamen_right_volume	Control Group	Asymptomatic non-hospitalised COVID-19	-,14013	,09460	,422	-,3693	,0890	
		Severe hospitalised COVID-19	,14313	,10193	,487	-,1038	,3901	
	Asymptomatic non-hospitalised COVID-19	Control Group	,14013	,09460	,422	-,0890	,3693	
		Severe hospitalised COVID-19	,28326*	,10396	,022	,0314	,5351	
	Severe hospitalised COVID-19	Control Group	-,14313	,10193	,487	-,3901	,1038	
		Asymptomatic non-hospitalised COVID-19	-,28326*	,10396	,022	-,5351	-,0314	
Putamen_right_percentile	Control Group	Asymptomatic non-hospitalised COVID-19	-4,26348	4,68929	1,000	-15,6231	7,0962	
		Severe hospitalised COVID-19	-,72532	5,05277	1,000	-12,9655	11,5149	
	Asymptomatic non-hospitalised COVID-19	Control Group	4,26348	4,68929	1,000	-7,0962	15,6231	
		Severe hospitalised COVID-19	3,53816	5,15345	1,000	-8,9459	16,0222	
	Severe hospitalised COVID-19	Control Group	,72532	5,05277	1,000	-11,5149	12,9655	
		Asymptomatic non-hospitalised COVID-19	-3,53816	5,15345	1,000	-16,0222	8,9459	
Putamen_left_volume	Control Group	Asymptomatic non-hospitalised COVID-19	-,14384	,09816	,435	-,3816	,0940	
		Severe hospitalised COVID-19	,16612	,10577	,356	-,0901	,4223	
	Asymptomatic non-hospitalised COVID-19	Control Group	,14384	,09816	,435	-,0940	,3816	
		Severe hospitalised COVID-19	,30995*	,10788	,014	,0486	,5713	
	Severe hospitalised COVID-19	Control Group	-,16612	,10577	,356	-,4223	,0901	
		Asymptomatic non-hospitalised COVID-19	-,30995*	,10788	,014	-,5713	-,0486	
Putamen_left_percentile	Control Group	Asymptomatic non-hospitalised COVID-19	-4,12279	4,77897	1,000	-15,6997	7,4541	
		Severe hospitalised COVID-19	1,86122	5,14941	1,000	-10,6131	14,3355	
	Asymptomatic non-hospitalised COVID-19	Control Group	4,12279	4,77897	1,000	-7,4541	15,6997	
		Severe hospitalised COVID-19	5,98401	5,25201	,769	-6,7388	18,7068	
	Severe hospitalised COVID-19	Control Group	-1,86122	5,14941	1,000	-14,3355	10,6131	
		Asymptomatic non-hospitalised COVID-19	-5,98401	5,25201	,769	-18,7068	6,7388	
Pallidum_right_volume	Control Group	Asymptomatic non-hospitalised COVID-19	-,04576	,03045	,405	-,1195	,0280	
		Severe hospitalised COVID-19	,06012	,03281	,207	-,0194	,1396	
	Asymptomatic non-hospitalised COVID-19	Control Group	,04576	,03045	,405	-,0280	,1195	
		Severe hospitalised COVID-19	,10588*	,03346	,006	,0248	,1869	
	Severe hospitalised COVID-19	Control Group	-,06012	,03281	,207	-,1396	,0194	
		Asymptomatic non-hospitalised COVID-19	-,10588*	,03346	,006	-,1869	-,0248	
Pallidum_right_percentile	Control Group	Asymptomatic non-hospitalised COVID-19	-4,07780	5,23128	1,000	-16,7504	8,5948	
		Severe hospitalised COVID-19	2,33819	5,63677	1,000	-11,3167	15,9931	
	Asymptomatic non-hospitalised COVID-19	Control Group	4,07780	5,23128	1,000	-8,5948	16,7504	
		Severe hospitalised COVID-19	6,41599	5,74909	,799	-7,5110	20,3430	
	Severe hospitalised COVID-19	Control Group	-2,33819	5,63677	1,000	-15,9931	11,3167	
		Asymptomatic non-hospitalised COVID-19	-6,41599	5,74909	,799	-20,3430	7,5110	
Pallidum_left_volume	Control Group	Asymptomatic non-hospitalised COVID-19	-,06082	,02950	,123	-,1323	,0106	
		Severe hospitalised COVID-19	,05728	,03178	,221	-,0197	,1343	
	Asymptomatic non-hospitalised COVID-19	Control Group	,06082	,02950	,123	-,0106	,1323	
		Severe hospitalised COVID-19	,11810*	,03242	,001	,0396	,1966	
	Severe hospitalised COVID-19	Control Group	-,05728	,03178	,221	-,1343	,0197	
		Asymptomatic non-hospitalised COVID-19	-,11810*	,03242	,001	-,1966	-,0396	
Pallidum_left_percentile	Control Group	Asymptomatic non-hospitalised COVID-19	-1,41908	4,51985	1,000	-12,3683	9,5301	
		Severe hospitalised COVID-19	4,21516	4,87020	1,000	-7,5827	16,0131	
	Asymptomatic non-hospitalised COVID-19	Control Group	1,41908	4,51985	1,000	-9,5301	12,3683	
		Severe hospitalised COVID-19	5,63424	4,96724	,776	-6,3987	17,6672	
	Severe hospitalised COVID-19	Control Group	-4,21516	4,87020	1,000	-16,0131	7,5827	
		Asymptomatic non-hospitalised COVID-19	-5,63424	4,96724	,776	-17,6672	6,3987	
Thalamus_right_volume	Control Group	Asymptomatic non-hospitalised COVID-19	-,21849	,15265	,464	-,5883	,1513	
		Severe hospitalised COVID-19	,40806*	,16449	,043	,0096	,8065	
	Asymptomatic non-hospitalised COVID-19	Control Group	,21849	,15265	,464	-,1513	,5883	
		Severe hospitalised COVID-19	,62655*	,16776	,001	,2201	1,0329	
	Severe hospitalised COVID-19	Control Group	-,40806*	,16449	,043	-,8065	-,0096	
		Asymptomatic non-hospitalised COVID-19	-,62655*	,16776	,001	-1,0329	-,2201	
Thalamus_right_percentile	Control Group	Asymptomatic non-hospitalised COVID-19	,29471	5,67029	1,000	-13,4414	14,0308	
		Severe hospitalised COVID-19	9,30060	6,10981	,390	-5,5002	24,1014	
	Asymptomatic non-hospitalised COVID-19	Control Group	-,29471	5,67029	1,000	-14,0308	13,4414	
		Severe hospitalised COVID-19	9,00588	6,23155	,452	-6,0899	24,1016	
	Severe hospitalised COVID-19	Control Group	-9,30060	6,10981	,390	-24,1014	5,5002	
		Asymptomatic non-hospitalised COVID-19	-9,00588	6,23155	,452	-24,1016	6,0899	
Thalamus_left_volume	Control Group	Asymptomatic non-hospitalised COVID-19	-,00756	,20061	1,000	-,4935	,4784	
		Severe hospitalised COVID-19	,41612	,21616	,169	-,1075	,9398	
	Asymptomatic non-hospitalised COVID-19	Control Group	,00756	,20061	1,000	-,4784	,4935	
		Severe hospitalised COVID-19	,42368	,22047	,170	-,1104	,9578	
	Severe hospitalised COVID-19	Control Group	-,41612	,21616	,169	-,9398	,1075	
		Asymptomatic non-hospitalised COVID-19	-,42368	,22047	,170	-,9578	,1104	
Thalamus_left_percentile	Control Group	Asymptomatic non-hospitalised COVID-19	2,40273	5,54147	1,000	-11,0213	15,8268	
		Severe hospitalised COVID-19	10,74753	5,97101	,222	-3,7171	25,2121	
	Asymptomatic non-hospitalised COVID-19	Control Group	-2,40273	5,54147	1,000	-15,8268	11,0213	
		Severe hospitalised COVID-19	8,34480	6,08998	,518	-6,4080	23,0976	
	Severe hospitalised COVID-19	Control Group	-10,74753	5,97101	,222	-25,2121	3,7171	
		Asymptomatic non-hospitalised COVID-19	-8,34480	6,08998	,518	-23,0976	6,4080	
Brainstem_volume	Control Group	Asymptomatic non-hospitalised COVID-19	1,48319	1,30952	,778	-1,6891	4,6555	
		Severe hospitalised COVID-19	3,04066	1,41103	,099	-,3775	6,4588	
	Asymptomatic non-hospitalised COVID-19	Control Group	-1,48319	1,30952	,778	-4,6555	1,6891	
		Severe hospitalised COVID-19	1,55747	1,43914	,843	-1,9288	5,0437	
	Severe hospitalised COVID-19	Control Group	-3,04066	1,41103	,099	-6,4588	,3775	
		Asymptomatic non-hospitalised COVID-19	-1,55747	1,43914	,843	-5,0437	1,9288	
Brainstem_percentile	Control Group	Asymptomatic non-hospitalised COVID-19	-,43438	5,13420	1,000	-12,8718	12,0031	
		Severe hospitalised COVID-19	8,06712	5,53217	,441	-5,3344	21,4686	
	Asymptomatic non-hospitalised COVID-19	Control Group	,43438	5,13420	1,000	-12,0031	12,8718	
		Severe hospitalised COVID-19	8,50151	5,64240	,402	-5,1670	22,1701	
	Severe hospitalised COVID-19	Control Group	-8,06712	5,53217	,441	-21,4686	5,3344	
		Asymptomatic non-hospitalised COVID-19	-8,50151	5,64240	,402	-22,1701	5,1670	
Mesencephalon_volume	Control Group	Asymptomatic non-hospitalised COVID-19	1,67934	1,48117	,776	-1,9087	5,2674	
		Severe hospitalised COVID-19	2,09020	1,59598	,577	-1,7760	5,9564	
	Asymptomatic non-hospitalised COVID-19	Control Group	-1,67934	1,48117	,776	-5,2674	1,9087	
		Severe hospitalised COVID-19	,41086	1,62778	1,000	-3,5324	4,3541	
	Severe hospitalised COVID-19	Control Group	-2,09020	1,59598	,577	-5,9564	1,7760	
		Asymptomatic non-hospitalised COVID-19	-,41086	1,62778	1,000	-4,3541	3,5324	
Mesencephalon_percentile	Control Group	Asymptomatic non-hospitalised COVID-19	-,40371	5,35964	1,000	-13,3873	12,5799	
		Severe hospitalised COVID-19	8,24863	5,77509	,466	-5,7413	22,2386	
	Asymptomatic non-hospitalised COVID-19	Control Group	,40371	5,35964	1,000	-12,5799	13,3873	
		Severe hospitalised COVID-19	8,65234	5,89016	,432	-5,6164	22,9211	
	Severe hospitalised COVID-19	Control Group	-8,24863	5,77509	,466	-22,2386	5,7413	
		Asymptomatic non-hospitalised COVID-19	-8,65234	5,89016	,432	-22,9211	5,6164	
Pons_volume	Control Group	Asymptomatic non-hospitalised COVID-19	1,49653	1,36505	,824	-1,8103	4,8033	
		Severe hospitalised COVID-19	2,28356	1,47086	,368	-1,2795	5,8467	
	Asymptomatic non-hospitalised COVID-19	Control Group	-1,49653	1,36505	,824	-4,8033	1,8103	
		Severe hospitalised COVID-19	,78703	1,50016	1,000	-2,8471	4,4211	
	Severe hospitalised COVID-19	Control Group	-2,28356	1,47086	,368	-5,8467	1,2795	
		Asymptomatic non-hospitalised COVID-19	-,78703	1,50016	1,000	-4,4211	2,8471	
Pons_percentile	Control Group	Asymptomatic non-hospitalised COVID-19	2,86366	5,36180	1,000	-10,1251	15,8525	
		Severe hospitalised COVID-19	8,63892	5,77741	,411	-5,3567	22,6345	
	Asymptomatic non-hospitalised COVID-19	Control Group	-2,86366	5,36180	1,000	-15,8525	10,1251	
		Severe hospitalised COVID-19	5,77526	5,89253	,986	-8,4992	20,0497	
	Severe hospitalised COVID-19	Control Group	-8,63892	5,77741	,411	-22,6345	5,3567	
		Asymptomatic non-hospitalised COVID-19	-5,77526	5,89253	,986	-20,0497	8,4992	
Cerebellar_grey_matter_volume	Control Group	Asymptomatic non-hospitalised COVID-19	-2,89961	2,17256	,552	-8,1626	2,3633	
		Severe hospitalised COVID-19	4,86735	2,34096	,118	-,8035	10,5383	
	Asymptomatic non-hospitalised COVID-19	Control Group	2,89961	2,17256	,552	-2,3633	8,1626	
		Severe hospitalised COVID-19	7,76697*	2,38760	,004	1,9831	13,5509	
	Severe hospitalised COVID-19	Control Group	-4,86735	2,34096	,118	-10,5383	,8035	
		Asymptomatic non-hospitalised COVID-19	-7,76697*	2,38760	,004	-13,5509	-1,9831	
Cerebellar_grey_matter_percentile	Control Group	Asymptomatic non-hospitalised COVID-19	-2,43866	4,99417	1,000	-14,5369	9,6596	
		Severe hospitalised COVID-19	9,25018	5,38128	,263	-3,7858	22,2862	
	Asymptomatic non-hospitalised COVID-19	Control Group	2,43866	4,99417	1,000	-9,6596	14,5369	
		Severe hospitalised COVID-19	11,68884	5,48851	,105	-1,6069	24,9846	
	Severe hospitalised COVID-19	Control Group	-9,25018	5,38128	,263	-22,2862	3,7858	
		Asymptomatic non-hospitalised COVID-19	-11,68884	5,48851	,105	-24,9846	1,6069	
Left_ventricle_volume	Control Group	Asymptomatic non-hospitalised COVID-19	1,58680	1,19326	,557	-1,3038	4,4774	
		Severe hospitalised COVID-19	2,22285	1,28576	,258	-,8919	5,3376	
	Asymptomatic non-hospitalised COVID-19	Control Group	-1,58680	1,19326	,557	-4,4774	1,3038	
		Severe hospitalised COVID-19	,63605	1,31138	1,000	-2,5407	3,8128	
	Severe hospitalised COVID-19	Control Group	-2,22285	1,28576	,258	-5,3376	,8919	
		Asymptomatic non-hospitalised COVID-19	-,63605	1,31138	1,000	-3,8128	2,5407	
Left_ventricle_percentile	Control Group	Asymptomatic non-hospitalised COVID-19	5,89282	5,66792	,901	-7,8375	19,6232	
		Severe hospitalised COVID-19	2,46809	6,10726	1,000	-12,3266	17,2627	
	Asymptomatic non-hospitalised COVID-19	Control Group	-5,89282	5,66792	,901	-19,6232	7,8375	
		Severe hospitalised COVID-19	-3,42474	6,22894	1,000	-18,5142	11,6647	
	Severe hospitalised COVID-19	Control Group	-2,46809	6,10726	1,000	-17,2627	12,3266	
		Asymptomatic non-hospitalised COVID-19	3,42474	6,22894	1,000	-11,6647	18,5142	
Right_ventricle_volume	Control Group	Asymptomatic non-hospitalised COVID-19	,89538	1,04888	1,000	-1,6455	3,4362	
		Severe hospitalised COVID-19	1,55842	1,13018	,510	-1,1794	4,2962	
	Asymptomatic non-hospitalised COVID-19	Control Group	-,89538	1,04888	1,000	-3,4362	1,6455	
		Severe hospitalised COVID-19	,66305	1,15270	1,000	-2,1293	3,4554	
	Severe hospitalised COVID-19	Control Group	-1,55842	1,13018	,510	-4,2962	1,1794	
		Asymptomatic non-hospitalised COVID-19	-,66305	1,15270	1,000	-3,4554	2,1293	
Right_ventricle_percentile	Control Group	Asymptomatic non-hospitalised COVID-19	3,58943	5,85824	1,000	-10,6020	17,7808	
		Severe hospitalised COVID-19	2,41658	6,31233	1,000	-12,8749	17,7080	
	Asymptomatic non-hospitalised COVID-19	Control Group	-3,58943	5,85824	1,000	-17,7808	10,6020	
		Severe hospitalised COVID-19	-1,17285	6,43811	1,000	-16,7690	14,4233	
	Severe hospitalised COVID-19	Control Group	-2,41658	6,31233	1,000	-17,7080	12,8749	
		Asymptomatic non-hospitalised COVID-19	1,17285	6,43811	1,000	-14,4233	16,7690	
Third_ventricle_volume	Control Group	Asymptomatic non-hospitalised COVID-19	-,07328	,07557	1,000	-,2564	,1098	
		Severe hospitalised COVID-19	-,05865	,08143	1,000	-,2559	,1386	
	Asymptomatic non-hospitalised COVID-19	Control Group	,07328	,07557	1,000	-,1098	,2564	
		Severe hospitalised COVID-19	,01463	,08305	1,000	-,1866	,2158	
	Severe hospitalised COVID-19	Control Group	,05865	,08143	1,000	-,1386	,2559	
		Asymptomatic non-hospitalised COVID-19	-,01463	,08305	1,000	-,2158	,1866	
Third_ventricle_percentile	Control Group	Asymptomatic non-hospitalised COVID-19	5,17031	5,54115	1,000	-8,2530	18,5936	
		Severe hospitalised COVID-19	-5,52894	5,97067	1,000	-19,9927	8,9348	
	Asymptomatic non-hospitalised COVID-19	Control Group	-5,17031	5,54115	1,000	-18,5936	8,2530	
		Severe hospitalised COVID-19	-10,69925	6,08964	,243	-25,4512	4,0527	
	Severe hospitalised COVID-19	Control Group	5,52894	5,97067	1,000	-8,9348	19,9927	
		Asymptomatic non-hospitalised COVID-19	10,69925	6,08964	,243	-4,0527	25,4512	
Fourth_ventricle_volume	Control Group	Asymptomatic non-hospitalised COVID-19	-,04321	,07380	1,000	-,2220	,1356	
		Severe hospitalised COVID-19	,10687	,07952	,543	-,0858	,2995	
	Asymptomatic non-hospitalised COVID-19	Control Group	,04321	,07380	1,000	-,1356	,2220	
		Severe hospitalised COVID-19	,15008	,08111	,199	-,0464	,3466	
	Severe hospitalised COVID-19	Control Group	-,10687	,07952	,543	-,2995	,0858	
		Asymptomatic non-hospitalised COVID-19	-,15008	,08111	,199	-,3466	,0464	
Fourth_ventricle_percentile	Control Group	Asymptomatic non-hospitalised COVID-19	-6,03915	5,55024	,835	-19,4844	7,4062	
		Severe hospitalised COVID-19	1,59057	5,98046	1,000	-12,8969	16,0781	
	Asymptomatic non-hospitalised COVID-19	Control Group	6,03915	5,55024	,835	-7,4062	19,4844	
		Severe hospitalised COVID-19	7,62971	6,09963	,639	-7,1464	22,4059	
	Severe hospitalised COVID-19	Control Group	-1,59057	5,98046	1,000	-16,0781	12,8969	
		Asymptomatic non-hospitalised COVID-19	-7,62971	6,09963	,639	-22,4059	7,1464	

*. The mean difference is significant at the 0.05 level.	
