# Supplementary material for: Brain Volume Changes after COVID-19 Compared to Healthy Controls by Artificial Intelligence-Based MRI Volumetry
Source: Diagnostics (Basel). 2023 May 12;13(10):1716. doi: 10.3390/diagnostics13101716 (PMC10216908; doi:10.3390/diagnostics13101716)
Supplement: Supplementary file 1 [file diagnostics-13-01716-s001.zip › Supplemental Table S3.docx]

**Supplementary Table S3:** Multivariant regression analysis estimating variable-related volumetric changes in respective brain regions

| **Brain Region** |  | **Estimate** | **Standard Error** | ***t*-Value** | ***p*-Value** |
| --- | --- | --- | --- | --- | --- |
| **Whole brain** | Age (years) | −2.4107 | 0.6102 | −3.951 | 0.000120 |
|  | Gender (male) | 97.1842 | 21.3302 | 4.556 | 1.08e-05 |
|  | COVID-19 Mild | 32.6959 | 18.3726 | 1.780 | 0.077195 |
|  | COVID-19 Severe | −36.1273 | 18.8432 | −1.917 | 0.057131 |
|  | Height | 4.0755 | 1.0574 | 3.854 | 0.000173 |
|  | BMI | −0.9211 | 1.1922 | −0.773 | 0.441023 |
|  | *Multiple R-squared* | 0.4932 |  | *p*-value | <2.2e-16 |
| **White matter** | Age (years) | −0.13652 | 0.33865 | −0.403 | 0.6874 |
|  | Gender (male) | 39.71106 | 11.83884 | 3.354 | 0.001 |
|  | COVID-19 Mild | 19.1882 | 10.19729 | 1.882 | 0.0618 |
|  | COVID-19 Severe | −11.21156 | 10.45846 | −1.072 | 0.2855 |
|  | Height | 2.28621 | 0.58689 | 3.895 | 0.0001 |
|  | BMI | −0.61808 | 0.66173 | −0.934 | 0.3518 |
|  | *Multiple R-squared* | 0.3938 |  | *p*-value | 3.766e-14 |
| **Grey matter** | Age (years) | −2.20669 | 0.33652 | −6.557 | 8.58e-10 |
|  | Gender (male) | 58.22570 | 11.76448 | 4.949 | 2.00e-06 |
|  | COVID-19 Mild | 13.84293 | 10.13324 | 1.366 | 0.17398 |
|  | COVID-19 Severe | −28.64409 | 10.39277 | −2.756 | 0.00658 |
|  | Height | 1.92446 | 0.58321 | 3.300 | 0.00121 |
|  | BMI | 0.05646 | 0.65757 | 0.086 | 0.93169 |
|  | *Multiple R-squared* | 0.5367 |  | *p*-value | <2.2e-16 |
| **Supratentorial** | Age (years) | −1.53934 | 0.24475 | −6.289 | 3.39e-09 |
| **cerebral** | Gender (male) | 39.97339 | 8.55623 | 4.671 | 6.64e-06 |
| **cortex** | COVID-19 Mild | 5.92185 | 7.36984 | 0.803 | 0.42295 |
|  | COVID-19 Severe | −19.36262 | 7.55859 | −2.561 | 0.01141 |
|  | Height | 1.37947 | 0.42416 | 3.252 | 0.00141 |
|  | BMI | −0.41309 | 0.47824 | −0.863 | 0.38911 |
|  | *Multiple R-squared* | 0.5097 |  | *p*-value | <2.2e-16 |
| **Frontal lobe** | Age (years) | −0.39890 | 0.04735 | −8.425 | 2.92e-14 |
| **right** | Gender (male) | 6.96303 | 1.65526 | 4.207 | 4.47e-05 |
|  | COVID-19 Mild | 1.15013 | 1.42575 | 0.807 | 0.42114 |
|  | COVID-19 Severe | −3.92092 | 1.46226 | −2.681 | 0.00816 |
|  | Height | 0.25741 | 0.08206 | 3.137 | 0.00206 |
|  | BMI | −0.09647 | 0.09252 | −1.043 | 0.29881 |
|  | *Multiple R-squared* | 0.5397 |  | *p*-value | <2.2e-16 |
| **Frontal lobe** | Age (years) | −0.35550 | 0.05445 | −6.529 | 9.96e-10 |
| **left** | Gender (male) | 7.28431 | 1.90364 | 3.827 | 0.000191 |
|  | COVID-19 Mild | 0.42527 | 1.63969 | 0.259 | 0.795716 |
|  | COVID-19 Severe | −3.48425 | 1.68168 | −2.072 | 0.040011 |
|  | Height | 0.27121 | 0.09437 | 2.874 | 0.004651 |
|  | BMI | −0.13552 | 0.10640 | −1.274 | 0.204797 |
|  | *Multiple R-squared* | 0.4524 |  | *p*-value | <2.2e-16 |
| **Parietal lobe** | Age (years) | −0.19783 | 0.02668 | −7.414 | 8.75e-12 |
| **right** | Gender (male) | 3.76298 | 0.93281 | 4.034 | 0.0001 |
|  | COVID-19 Mild | 0.17747 | 0.80347 | 0.221 | 0.8255 |
|  | COVID-19 Severe | −2.05789 | 0.82405 | −2.497 | 0.0136 |
|  | Height | 0.13298 | 0.04624 | 2.876 | 0.0046 |
|  | BMI | −0.00355 | 0.05214 | −0.068 | 0.9458 |
|  | *Multiple R-squared* | 0.4866 |  | *p*-value | <2.2e-16 |
| **Parietal lobe** | Age (years) | −0.18253 | 0.02821 | −6.47 | 1.35e-09 |
| **left** | Gender (male) | 4.09906 | 0.98632 | 4.156 | 0.0001 |
|  | COVID-19 Mild | 0.15045 | 0.84956 | 0.177 | 0.8597 |
|  | COVID-19 Severe | −2.4184 | 0.87132 | −2.776 | 0.0062 |
|  | Height | 0.13495 | 0.0489 | 2.76 | 0.0065 |
|  | BMI | −0.048 | 0.05513 | −0.871 | 0.3853 |
|  | *Multiple R-squared* | 0.4701 |  | *p*-value | <2.2e-16 |
| **Precuneus** | Age (years) | −0.0379 | 0.00757 | −5.01 | 1.53e-06 |
| **right** | Gender (male) | 1.1321 | 0.26447 | 4.281 | 3.33e-05 |
|  | COVID-19 Mild | 0.16744 | 0.2278 | 0.735 | 0.4635 |
|  | COVID-19 Severe | −0.4825 | 0.23363 | −2.065 | 0.0406 |
|  | Height | 0.03398 | 0.01311 | 2.592 | 0.0105 |
|  | BMI | 0.00059 | 0.01478 | 0.04 | 0.9682 |
|  | *Multiple R-squared* | 0.4255 |  | *p*-value | 8.19e-16 |
| **Precuneus** | Age (years) | −0.04513 | 0.01004 | −4.494 | 1.40e-05 |
| **left** | Gender (male) | 1.7543 | 0.35105 | 4.997 | 1.62e-06 |
|  | COVID-19 Mild | 0.1357 | 0.30237 | 0.449 | 0.6542 |
|  | COVID-19 Severe | −0.53504 | 0.31012 | −1.725 | 0.0866 |
|  | Height | 0.0113 | 0.0174 | 0.649 | 0.5172 |
|  | BMI | −0.00786 | 0.01962 | −0.4 | 0.6895 |
|  | *Multiple R-squared* | 0.3529 |  | *p*-value | 3.825e-12 |
| **Occipital lobe** | Age (years) | −0.06506 | 0.0399 | −1.63 | 0.1051 |
| **right** | Gender (male) | 5.53616 | 1.39499 | 3.969 | 0.0001 |
|  | COVID-19 Mild | 1.36311 | 1.20157 | 1.134 | 0.2584 |
|  | COVID-19 Severe | −1.19923 | 1.23234 | −0.973 | 0.3321 |
|  | Height | −0.06946 | 0.06915 | −1.004 | 0.3168 |
|  | BMI | −0.03723 | 0.07797 | −0.477 | 0.6338 |
|  | *Multiple R-squared* | 0.1635 |  | *p*-value | 0.00016 |
| **Occipital lobe** | Age (years) | −0.07167 | 0.04324 | −1.658 | 0.0995 |
| **left** | Gender (male) | 1.62171 | 1.51154 | 1.073 | 0.2851 |
|  | COVID-19 Mild | −0.10926 | 1.30195 | −0.084 | 0.9332 |
|  | COVID-19 Severe | −2.81361 | 1.3353 | −2.107 | 0.0368 |
|  | Height | 0.14092 | 0.07493 | 1.881 | 0.062 |
|  | BMI | 0.05991 | 0.08449 | 0.709 | 0.4794 |
|  | *Multiple R-squared* | 0.1398 |  | *p*-value | 0.0009485 |
| **Temporal lobe** | Age (years) | −0.1551 | 0.03995 | −3.882 | 0.0002 |
| **right** | Gender (male) | 5.56413 | 1.39655 | 3.984 | 0.0001 |
|  | COVID-19 Mild | 2.12769 | 1.2029 | 1.769 | 0.079 |
|  | COVID-19 Severe | −2.06131 | 1.23371 | −1.671 | 0.0969 |
|  | Height | 0.26713 | 0.06923 | 3.859 | 0.0002 |
|  | BMI | −0.01036 | 0.07806 | −0.133 | 0.8945 |
|  | *Multiple R-squared* | 0.46 |  | *p*-value | < 2.2e-16 |
| **Temporal lobe** | Age (years) | −0.13364 | 0.04693 | −2.847 | 0.005 |
| **left** | Gender (male) | 5.29721 | 1.64078 | 3.228 | 0.0015 |
|  | COVID-19 Mild | 1.59621 | 1.41327 | 1.129 | 0.2605 |
|  | COVID-19 Severe | −1.62364 | 1.44947 | −1.12 | 0.2645 |
|  | Height | 0.18689 | 0.08134 | 2.298 | 0.023 |
|  | BMI | −0.03603 | 0.09171 | −0.393 | 0.695 |
|  | *Multiple R-squared* | 0.2964 |  | *p*-value | 1.34e-09 |
| **Mesiotemporal** | Age (years) | −0.19888 | 0.06781 | −2.933 | 0.0039 |
| **right** | Gender (male) | 2.57378 | 2.37057 | 1.086 | 0.2794 |
|  | COVID-19 Mild | 2.21279 | 2.04187 | 1.084 | 0.2803 |
|  | COVID-19 Severe | 0.96327 | 2.09417 | 0.46 | 0.6462 |
|  | Height | 0.02642 | 0.11752 | 0.225 | 0.8224 |
|  | BMI | −0.09826 | 0.1325 | −0.742 | 0.4595 |
|  | *Multiple R-squared* | 0.08074 |  | *p*-value | 0.04937 |
| **Mesiotemporal** | Age (years) | −0.19063 | 0.0621 | −3.07 | 0.0026 |
| **left** | Gender (male) | 2.2926 | 2.17097 | 1.056 | 0.2927 |
|  | COVID-19 Mild | 2.06097 | 1.86995 | 1.102 | 0.2722 |
|  | COVID-19 Severe | 0.84468 | 1.91784 | 0.44 | 0.6603 |
|  | Height | 0.01255 | 0.10762 | 0.117 | 0.9073 |
|  | BMI | −0.10651 | 0.12135 | −0.878 | 0.3815 |
|  | *Multiple R-squared* | 0.0849 |  | *p*-value | 0.03843 |
| **Hippocampus** | Age (years) | −0.00906 | 0.02299 | −0.394 | 0.6941 |
| **right** | Gender (male) | 0.06297 | 0.8036 | 0.078 | 0.9377 |
|  | COVID-19 Mild | −0.6457 | 0.69217 | −0.933 | 0.3524 |
|  | COVID-19 Severe | −0.75283 | 0.7099 | −1.06 | 0.2907 |
|  | Height | 0.05653 | 0.03984 | 1.419 | 0.158 |
|  | BMI | 0.02641 | 0.04492 | 0.588 | 0.5574 |
|  | *Multiple R-squared* | 0.03963 |  | *p*-value | 0.416 |
| **Hippocampus** | Age (years) | −0.0214 | 0.04431 | −0.483 | 0.6299 |
| **left** | Gender (male) | −0.41047 | 1.54891 | −0.265 | 0.7914 |
|  | COVID-19 Mild | −0.62528 | 1.33414 | −0.469 | 0.64 |
|  | COVID-19 Severe | −1.23179 | 1.36831 | −0.9 | 0.3695 |
|  | Height | 0.09472 | 0.07678 | 1.234 | 0.2193 |
|  | BMI | 0.04865 | 0.08658 | 0.562 | 0.575 |
|  | *Multiple R-squared* | 0.02363 |  | *p*-value | 0.7324 |
| **Gyrus para-** | Age (years) | −0.00406 | 0.00204 | −1.994 | 0.048 |
| **hippocampalis** | Gender (male) | 0.2537 | 0.07114 | 3.566 | 0.0005 |
| **right** | COVID-19 Mild | 0.12969 | 0.06128 | 2.116 | 0.036 |
|  | COVID-19 Severe | −0.01374 | 0.06285 | −0.219 | 0.8272 |
|  | Height | 0.01198 | 0.00353 | 3.397 | 0.0009 |
|  | BMI | −0.00379 | 0.00398 | −0.952 | 0.3425 |
|  | *Multiple R-squared* | 0.3745 |  | *p*-value | 3.472e-13 |
| **Gyrus para-** | Age (years) | −0.00176 | 0.00191 | −0.921 | 0.3584 |
| **hippocampalis** | Gender (male) | 0.23806 | 0.0669 | 3.559 | 0.0005 |
| **left** | COVID-19 Mild | 0.14446 | 0.05762 | 2.507 | 0.0133 |
|  | COVID-19 Severe | −0.05742 | 0.0591 | −0.972 | 0.3328 |
|  | Height | 0.00796 | 0.00332 | 2.401 | 0.0176 |
|  | BMI | −0.00601 | 0.00374 | −1.608 | 0.11 |
|  | *Multiple R-squared* | 0.3334 |  | *p*-value | 3.079e-11 |
| **Regio** | Age (years) | 0.00115 | 0.00158 | 0.725 | 0.4695 |
| **entorhinalis** | Gender (male) | 0.22105 | 0.05535 | 3.994 | 0.0001 |
| **right** | COVID-19 Mild | 0.09207 | 0.04767 | 1.931 | 0.0553 |
|  | COVID-19 Severe | −0.02341 | 0.04889 | −0.479 | 0.6328 |
|  | Height | 0.00708 | 0.00274 | 2.579 | 0.0109 |
|  | BMI | −0.00107 | 0.00309 | −0.345 | 0.7308 |
|  | *Multiple R-squared* | 0.3501 |  | *p*-value | 5.166e-12 |
| **Regio** | Age (years) | 0.00023 | 0.00172 | 0.131 | 0.8957 |
| **entorhinalis** | Gender (male) | 0.25127 | 0.05997 | 4.19 | 4.78e-05 |
| **left** | COVID-19 Mild | 0.05859 | 0.05165 | 1.134 | 0.2585 |
|  | COVID-19 Severe | −0.02626 | 0.05298 | −0.496 | 0.6209 |
|  | Height | 0.00234 | 0.00297 | 0.787 | 0.4325 |
|  | BMI | −0.00243 | 0.00335 | −0.724 | 0.4704 |
|  | *Multiple R-squared* | 0.2426 |  | *p*-value | 2.146e-07 |
| **Nucleus** | Age (years) | −0.00964 | 0.00257 | −3.753 | 0.0003 |
| **caudatus** | Gender (male) | 0.23133 | 0.08977 | 2.577 | 0.0109 |
| **right** | COVID-19 Mild | 0.16848 | 0.07732 | 2.179 | 0.0309 |
|  | COVID-19 Severe | −0.07699 | 0.0793 | −0.971 | 0.3333 |
|  | Height | −0.00042 | 0.00445 | −0.094 | 0.925 |
|  | BMI | −0.00308 | 0.00502 | −0.614 | 0.5403 |
|  | *Multiple R-squared* | 0.2055 |  | *p*-value | 5.405e-06 |
| **Nucleus** | Age (years) | −0.01054 | 0.00227 | −4.648 | 7.35e-06 |
| **caudatus** | Gender (male) | 0.15347 | 0.07925 | 1.937 | 0.0547 |
| **left** | COVID-19 Mild | 0.20998 | 0.06826 | 3.076 | 0.0025 |
|  | COVID-19 Severe | −0.04397 | 0.07001 | −0.628 | 0.5309 |
|  | Height | −0.00134 | 0.00393 | −0.341 | 0.7334 |
|  | BMI | −0.00782 | 0.00443 | −1.765 | 0.0797 |
|  | *Multiple R-squared* | 0.2502 |  | *p*-value | 1.079e-07 |
| **Putamen** | Age (years) | −0.01466 | 0.0026 | −5.629 | 8.82e-08 |
| **right** | Gender (male) | 0.40684 | 0.09104 | 4.469 | 1.55e-05 |
|  | COVID-19 Mild | 0.10656 | 0.07842 | 1.359 | 0.1763 |
|  | COVID-19 Severe | −0.12023 | 0.08043 | −1.495 | 0.1371 |
|  | Height | 0.00266 | 0.00451 | 0.589 | 0.5569 |
|  | BMI | −0.00076 | 0.00509 | −0.149 | 0.8814 |
|  | *Multiple R-squared* | 0.3647 |  | *p*-value | 1.041e-12 |
| **Putamen** | Age (years) | −0.01538 | 0.00269 | −5.712 | 5.93e-08 |
| **left** | Gender (male) | 0.4343 | 0.09414 | 4.613 | 8.51e-06 |
|  | COVID-19 Mild | 0.10859 | 0.08109 | 1.339 | 0.1825 |
|  | COVID-19 Severe | −0.1375 | 0.08316 | −1.653 | 0.1004 |
|  | Height | 0.00157 | 0.00467 | 0.336 | 0.7376 |
|  | BMI | −0.00224 | 0.00526 | −0.426 | 0.6705 |
|  | *Multiple R-squared* | 0.368 |  | *p*-value | 7.221e-13 |
| **Pallidum** | Age (years) | −0.00234 | 0.0009 | −2.593 | 0.0105 |
| **right** | Gender (male) | 0.09729 | 0.03153 | 3.085 | 0.0024 |
|  | COVID-19 Mild | 0.04087 | 0.02716 | 1.505 | 0.1346 |
|  | COVID-19 Severe | −0.03631 | 0.02786 | −1.303 | 0.1945 |
|  | Height | 0.00287 | 0.00156 | 1.833 | 0.0688 |
|  | BMI | 0.00073 | 0.00176 | 0.413 | 0.6803 |
|  | *Multiple R-squared* | 0.2707 |  | *p*-value | 1.605e-08 |
| **Pallidum** | Age (years) | −0.0016 | 0.00089 | −1.797 | 0.0743 |
| **left** | Gender (male) | 0.09074 | 0.03122 | 2.907 | 0.0042 |
|  | COVID-19 Mild | 0.0569 | 0.02689 | 2.116 | 0.036 |
|  | COVID-19 Severe | −0.03032 | 0.02758 | −1.1 | 0.2733 |
|  | Height | 0.00278 | 0.00155 | 1.796 | 0.0745 |
|  | BMI | 0.00085 | 0.00174 | 0.488 | 0.6262 |
|  | *Multiple R-squared* | 0.2576 |  | *p*-value | 5.465e-08 |
| **Thalamus** | Age (years) | −0.02283 | 0.00425 | −5.375 | 2.91e-07 |
| **right** | Gender (male) | 0.3445 | 0.14851 | 2.32 | 0.0217 |
|  | COVID-19 Mild | 0.20392 | 0.12792 | 1.594 | 0.113 |
|  | COVID-19 Severe | −0.33729 | 0.13119 | −2.571 | 0.0111 |
|  | Height | 0.0214 | 0.00736 | 2.906 | 0.0042 |
|  | BMI | −0.00289 | 0.0083 | −0.348 | 0.7284 |
|  | *Multiple R-squared* | 0.3903 |  | *p*-value | 5.652e-14 |
| **Thalamus** | Age (years) | −0.02651 | 0.00573 | −4.628 | 8.01e-06 |
| **left** | Gender (male) | 0.47448 | 0.20027 | 2.369 | 0.0191 |
|  | COVID-19 Mild | −0.0148 | 0.1725 | −0.086 | 0.9318 |
|  | COVID-19 Severe | −0.32297 | 0.17692 | −1.825 | 0.0699 |
|  | Height | 0.0231 | 0.00993 | 2.326 | 0.0214 |
|  | BMI | −0.00357 | 0.01119 | −0.319 | 0.7499 |
|  | *Multiple R-squared* | 0.293 |  | *p*-value | 1.87e-09 |
| **Brainstem** | Age (years) | 0.0099 | 0.04076 | 0.243 | 0.8083 |
|  | Gender (male) | 1.54783 | 1.4248 | 1.086 | 0.2791 |
|  | COVID-19 Mild | −1.4237 | 1.22724 | −1.16 | 0.2479 |
|  | COVID-19 Severe | −2.84074 | 1.25867 | −2.257 | 0.0255 |
|  | Height | 0.15566 | 0.07063 | 2.204 | 0.0291 |
|  | BMI | 0.05938 | 0.07964 | 0.746 | 0.4571 |
|  | *Multiple R-squared* | 0.1434 |  | *p*-value | 0.0007309 |
| **Mesencephalon** | Age (years) | −0.00243 | 0.04802 | −0.051 | 0.9597 |
|  | Gender (male) | 0.53691 | 1.67889 | 0.32 | 0.7496 |
|  | COVID-19 Mild | −1.64283 | 1.4461 | −1.136 | 0.2578 |
|  | COVID-19 Severe | −1.97987 | 1.48314 | −1.335 | 0.184 |
|  | Height | 0.10739 | 0.08323 | 1.29 | 0.199 |
|  | BMI | 0.05471 | 0.09384 | 0.583 | 0.5607 |
|  | *Multiple R-squared* | 0.04618 |  | *p*-value | 0.3125 |
| **Pons** | Age (years) | 0.00169 | 0.04359 | 0.039 | 0.9692 |
|  | Gender (male) | 0.82918 | 1.52389 | 0.544 | 0.5872 |
|  | COVID-19 Mild | −1.46115 | 1.31259 | −1.113 | 0.2674 |
|  | COVID-19 Severe | −2.15063 | 1.34621 | −1.598 | 0.1123 |
|  | Height | 0.13281 | 0.07554 | 1.758 | 0.0808 |
|  | BMI | 0.07379 | 0.08518 | 0.866 | 0.3877 |
|  | *Multiple R-squared* | 0.08015 |  | *p*-value | 0.05113 |
| **Cerebellar** | Age (years) | −0.31019 | 0.05941 | −5.221 | 5.92e-07 |
| **grey matter** | Gender (male) | 7.87562 | 2.07686 | 3.792 | 0.0002 |
|  | COVID-19 Mild | 2.29969 | 1.78889 | 1.286 | 0.2006 |
|  | COVID-19 Severe | −2.36223 | 1.83471 | −1.288 | 0.1999 |
|  | Height | 0.23536 | 0.10296 | 2.286 | 0.0237 |
|  | BMI | 0.10044 | 0.11609 | 0.865 | 0.3883 |
|  | *Multiple R-squared* | 0.3879 |  | *p*-value | 7.511e-14 |
| **Left ventricle** | Age (years) | 0.22006 | 0.03381 | 6.508 | 1.11e-09 |
|  | Gender (male) | 1.21149 | 1.18213 | 1.025 | 0.3071 |
|  | COVID-19 Mild | −1.14525 | 1.01822 | −1.125 | 0.2625 |
|  | COVID-19 Severe | −1.70621 | 1.0443 | −1.634 | 0.1044 |
|  | Height | 0.16847 | 0.0586 | 2.875 | 0.0046 |
|  | BMI | −0.00399 | 0.06608 | −0.06 | 0.9519 |
|  | *Multiple R-squared* | 0.3437 |  | *p*-value | 1.039e-11 |
| **Right ventricle** | Age (years) | 0.21154 | 0.03095 | 6.834 | 2.01e-10 |
|  | Gender (male) | 1.51601 | 1.08212 | 1.401 | 0.1633 |
|  | COVID-19 Mild | −0.48534 | 0.93208 | −0.521 | 0.6033 |
|  | COVID-19 Severe | −0.79224 | 0.95595 | −0.829 | 0.4086 |
|  | Height | 0.11354 | 0.05364 | 2.117 | 0.036 |
|  | BMI | −0.05279 | 0.06049 | −0.873 | 0.3842 |
|  | *Multiple R-squared* | 0.335 |  | *p*-value | 2.603e-11 |
| **Third ventricle** | Age (years) | 0.01468 | 0.0021 | 7.001 | 8.24e-11 |
|  | Gender (male) | 0.15622 | 0.07331 | 2.131 | 0.0347 |
|  | COVID-19 Mild | 0.09088 | 0.06314 | 1.439 | 0.1522 |
|  | COVID-19 Severe | 0.04829 | 0.06476 | 0.746 | 0.457 |
|  | Height | 0.00233 | 0.00363 | 0.642 | 0.5218 |
|  | BMI | −.00256 | 0.0041 | −0.625 | 0.5331 |
|  | *Multiple R-squared* | 0.3241 |  | *p*-value | 8.152e-11 |
| **Fourth** | Age (years) | −0.00078 | 0.00233 | −.334 | 0.7389 |
| **ventricle** | Gender (male) | 0.17258 | 0.08128 | 2.123 | 0.0354 |
|  | COVID-19 Mild | 0.04612 | 0.07001 | 0.659 | 0.5111 |
|  | COVID-19 Severe | −0.04521 | 0.07181 | −0.63 | 0.5299 |
|  | Height | 0.00835 | 0.00403 | 2.073 | 0.0399 |
|  | BMI | −0.00115 | 0.00454 | −0.253 | 0.8007 |
|  | *Multiple R-squared* | 0.1716 |  | *p*-value | 8.518e-05 |
